# A modular synthetic approach for band-gap engineering of armchair graphene nanoribbons

**DOI:** 10.1038/s41467-018-03747-2

**Published:** 2018-04-27

**Authors:** Gang Li, Ki-Young Yoon, Xinjue Zhong, Jianchun Wang, Rui Zhang, Jeffrey R. Guest, Jianguo Wen, X.-Y. Zhu, Guangbin Dong

**Affiliations:** 1 Department of Chemistry, University of Chicago, Chicago, IL 60637 USA; 2 Department of Chemistry, University of Texas at Austin, Texas, 78712 USA; 3 Department of Chemistry, Columbia University, New York, 10027 USA; 4 Center for Nanoscale Materials, Argonne National Laboratory, Argonne, IL 60439 USA

## Abstract

Despite the great promise of armchair graphene nanoribbons (aGNRs) as high-performance semiconductors, practical band-gap engineering of aGNRs remains an unmet challenge. Given that width and edge structures are the two key factors for modulating band-gaps of aGNRs, a reliable synthetic method that allows control of both factors would be highly desirable. Here we report a simple modular strategy for efficient preparation of *N* = 6 aGNR, the narrowest member in the *N* = 3*p* (*p*: natural number) aGNR family, and two unsymmetrically edge-functionalized GNRs that contain benzothiadiazole and benzotriazole moieties. The trend of band-gap transitions among these GNRs parallels those in donor–acceptor alternating conjugated polymers. In addition, post-functionalization of the unsymmetrical heterocyclic edge via C–H borylation permits further band-gap tuning. Therefore, this method opens the door for convenient band-gap engineering of aGNRs through modifying the heteroarenes on the edge.

## Abstract (web summary)

Effective band-gap engineering of armchair graphene nanoribbons calls for control over both width and edge structure. Here, the authors report a modular synthesis of narrow *N* = 6 armchair graphene nanoribbons whose edges can be unsymmetrically modified with heteroarenes, introducing a simple way to tune band gap.

## Introduction

**Fig. 1:**
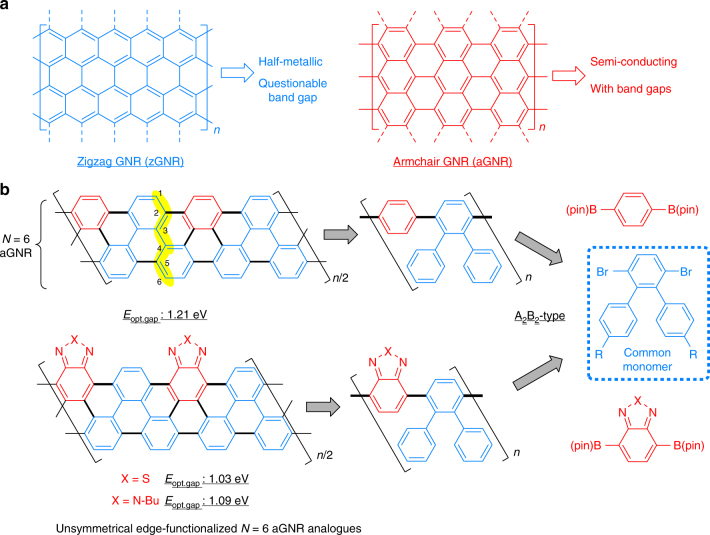
Graphene nanoribbons (GNRs). **a** Zigzag and armchair graphene nanoribbons (aGNRs). **b** A modular approach for aGNR synthesis. *N*: the number of dimer lines. *n*: the number of repeating units. pin: pinacol group. *E*_opt.gap_: optical band-gap energy

Graphene nanoribbons (GNRs) have recently emerged as attractive organic materials for applications in new generations of electronic devices (Fig. 1a)^1–9^. Grouped by their edge structures, zigzag, and armchair GNRs are the two types commonly studied. While zigzag GNRs (zGNRs) possesses intrinsically metallic properties^5, 6^, armchair GNRs (aGNRs) that are <10 nm wide are considered novel organic semiconductors. They not only have non-zero band-gaps due to quantum confinement but also possess much higher theoretical charge carrier mobility (>100 cm^2^ V^−1^ s^−1^) than regular conjugate polymers^7, 8^. Thus, aGNRs hold exceptional potential for use in electronic devices, e.g., field-effect transistors^1^. To date, aGNRs have been prepared via either top-down or bottom-up approaches. While the top-down approaches^10^, using graphene^11^, carbon nanotubes^12, 13^, or graphite^14^ as the starting materials, are straightforward, it is challenging to prepare narrow GNRs (<10 nm) with structural precision and edge functionalities^1–9^. The bottom-up approaches that employ small molecular precursors and polymerization techniques instead have shown great promise for controlling the width and edge structures of GNRs^1^. For example, a surface-based protocol has been used to prepare atomically precise *N* = 5^15, 16^, 7^17, 18^, and *N* = 13^18, 19^ aGNRs from organic monomers on Au(111) or Ag(111) single crystals; nevertheless, this metal-surface-based strategy requires high-reaction temperatures (>300 °C), which is generally not suitable for preparing GNRs with various functional groups or on a large scale^4^. Alternatively, the solution-phase bottom-up synthesis of GNRs features high scalability, improved processability, and flexibility for introducing different functional groups^20–27^. Seminal work by Müllen and co-workers first demonstrated the feasibility of solution-phase synthesis of *N* = 9^20^ and *N* = 18^21^ aGNRs, though the use of complex monomers in these syntheses limits the lengths and practicality of the materials. Recently, we disclosed a triaryl-monomer-based strategy for the synthesis of *N* = 9 aGNRs with improved molecular weights via an AB-type polymerization^28^. Concurrently, an elegant approach involving alkyne benzannulation was reported by Chalifoux and co-workers, which provides an innovative path to generate soluble *N* = 5 aGNRs with high efficiency^29^. More recently, the Wu group reported the synthesis of oligomeric rylene ribbons with interesting diradical characters^30^.

Since width (the *N*-value) and edge structures are two key factors for band-gap engineering of aGNR materials, a flexible method that enables control of both factors would be highly desirable. Here we describe the development of a modular approach for the preparation of the narrower *N* = 6 aGNRs and their edge conjugately functionalized analogues, namely benzothiadiazole and benzotriazole-derived ribbons, via alternating co-polymerization of a triaryl monomer and a 1,4-diborated aryl monomer (Fig. 1b). As the narrowest member within the *N* = 3*p* aGNR family (*p*: natural number, *N* = 3 aGNR is a typical conjugated polymer of poly(*para*-phenylene)), *N* = 6 aGNR is predicted to exhibit significantly different charge density distribution from the *N* = 9 one; moreover, neither pristine nor doped *N* = 6 aGNR materials have been selectively produced via surface or solution-based methods. Hence, first, this work offers a convenient access to these novel nanomaterials. Second, benzothiadiazole^31^ and benzotriazole moieties^32^ are commonly used as electron-withdrawing units^33^ in donor–acceptor conjugate polymers to modulate band structures; however, GNRs derived from these important heteroarenes were previously unknown. Thus, this work demonstrates a distinct way to engineer the band-gap of aGNRs through modifying the heteroarenes on the edge. Third, the strategy developed here has addressed an unmet challenge for preparing GNRs with unsymmetrical edges, which not only leads to new materials with unsymmetrical electron-density distribution but also provides an opportunity for further bandgap tuning via post-functionalizations.

## Results

### Synthesis of model nanographenes G1–G3

**Fig. 2:**
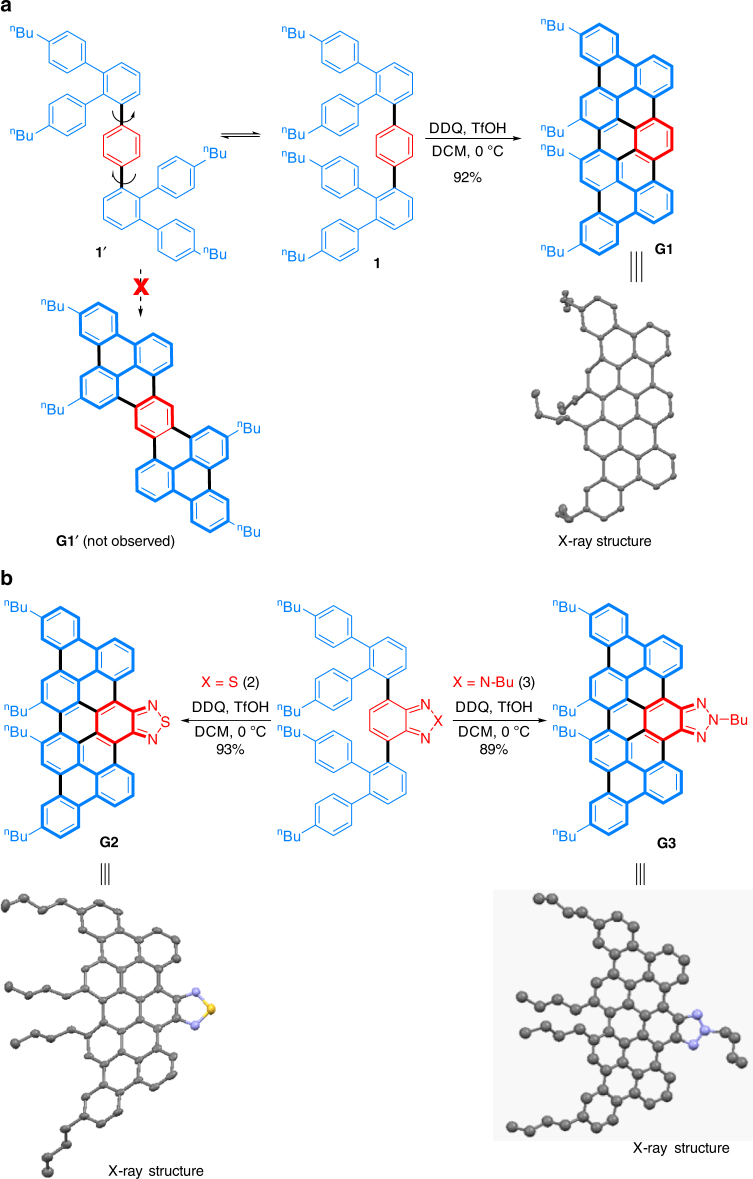
Synthesis of model nanographenes. **a** Regioselective synthesis of pristine nanographene **G1** as a model for *N* = 6 aGNRs. **b** Synthesis of edge-functionalized nanographenes (**G2** and **G3**). DDQ: 2,3-dichloro-5,6-dicyano-1,4-benzoquinone. TfOH: trifluoromethanesulfonic acid

Regarding the synthesis of *N* = 6 aGNRs (Fig. 1b), when a single-aryl co-monomer (e.g., 1,4-diborated benzene) is used, controlling the orientation of the aryl groups during the cyclodehydrogenation step becomes a key concern as the phenylene moieties can almost rotate freely. Thus, preparation of the corresponding nanographenes was carried out as a model study (Fig. 2). Precursor **1**, a defined segment of the polymer precursor for *N* = 6 aGNRs, was conveniently prepared via Suzuki coupling from commercially available 1,4-phenyldiboronic acid pinacol ester (**M1**) and the triaryl mono bromide (Supplementary Methods). Due to the free rotation of the axial C–C bonds, either the ribbon-like compound **G1** or the bis-diamond-like compound **G1′** could potentially be formed. To our delight, slow addition of TfOH into a solution of **1** and DDQ in DCM at 0 °C led to the formation of ribbon **G1** in a high yield (Fig. 2a). While similar observation has been made in other simpler systems^25, 34, 35^, the exact reason for such high regioselectivity remains unclear. It is noteworthy that possible side products, such as OTf-substituted or oxidative aryl–aryl coupling products, were not detected in the crude reaction mixture (Supplementary Fig. 1). In addition, the benzothiadiazole- and benzotriazole-derived analogues (**G2** and **G3**) were efficiently prepared by a similar route (Fig. 2b). All the model nanographenes were unambiguously characterized via ^1^H/^13^C NMR, FTIR spectroscopy, MALDI-TOF MS, and X-ray crystallography.

### Synthesis of GNR G4–G6

Encouraged by the model study, synthesis of *N* = 6 aGNR polymer precursors was pursued (Table 1). Using Pd(PtBu_3_)_2_/K_3_PO_4_ as the catalyst-based combination^28^, Suzuki polymerizations between bispinacol borate **M1**, **M2**, or **M3** and triaryl dibromide **M4** provided the desired poly(*para*-phenylenes) **P1**–**P3** in excellent yields and relatively high-molecular weights (entries 1, 3, and 5). Shorter polymers were prepared using a higher concentration of the base and/or a shorter reaction time (entries 2, 4, and 6). These materials were all successfully characterized by SEC and MALDI-TOF MS analysis due to their excellent solubility. The end groups were found to be mainly phenyl groups for **P1** and **P2**, and hydrogens for **P3** (Supplementary Fig. 5-7).

**Table 1:** Selected polymerization study to prepare GNR precursors


Entry	Monomer	*k*	Time (h)	Yield^a^ (%)	*M*_*n*_^b^ (kDa)	*M*_*w*_^b^ (kDa)	*Đ* ^b^
1	**M1**	3	24	93	22.5	44.9	2.09
				[73	28.9	49.4	1.71]^c^
2	**M1**	5	12	96	15.8	28.1	1.71
				[68	19.6	28.1	1.43]^c^
3	**M1**	3	24	97	36.8	80.2	2.18
				[95	37.8	80.1	2.12]^c^
4	**M1**	5	12	94	12.2	21.4	1.76
				[70	17.4	25.4	1.46]^c^
5	**M1**	3	24	95	22.6	40.5	1.79
				[82	27.6	43.4	1.57]^c^
6	**M1**	3	8	82	17.5	34.9	2.00
				[66	21.6	37.4	1.73]^c^

^a^ Isolated yield

^b^ Determined by THF SEC calibrated using polystyrene standards

^c^ After Soxhlet extraction under reflux of acetone

**Fig. 3:**
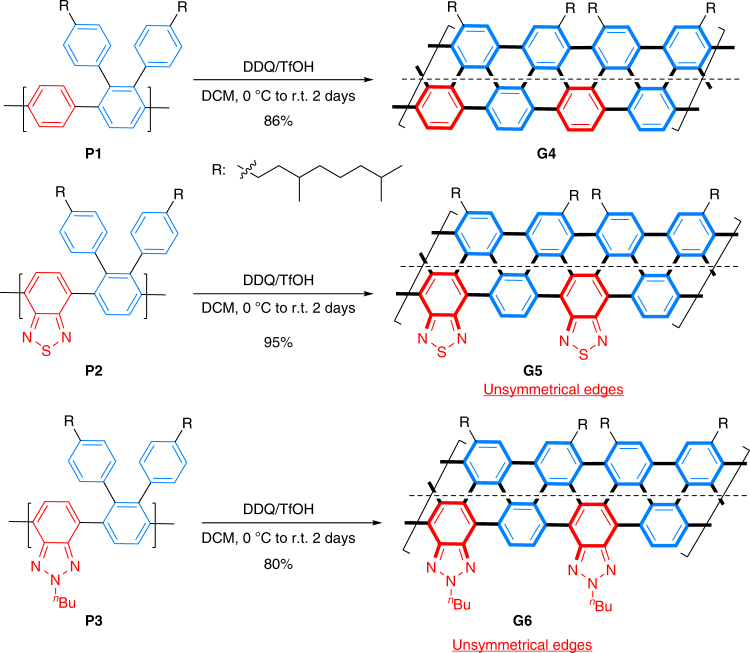
Synthesis of graphene nanoribbons. Syntheses of *N* = 6 aGNR and its edge-functionalized analogues

To obtain aGNRs with similar lengths, polymer precursors **P1**–**P3** with close molecular weights (prepared from entries 2, 4, and 6, respectively, after Soxhlet extraction) were cyclodehydrogenated using a similar DDQ/TfOH protocol (Fig. 3). (Note that longer polymer precursors can also be cyclodehydrogenated to aGNRs (over 86 nm) via the same protocols.) The *N* = 6 pristine (**G4**), benzothiadiazole-derived (**G5**) and benzotriazole-derived aGNRs (**G6**) were isolated as black powders after Soxhlet extraction. While they are only marginally soluble in common organic solvents, **G4–G6** can be well dispersed in THF, chlorobenzene, and *o*-dichlorobenzene. Thus, they have been characterized by FTIR/Raman/UV–Vis–NIR spectroscopy, XPS, MALDI-TOF MS, atomic force microscopy (AFM), and scanning tunneling microscopy (STM).

### Characterization of GNR G4–G6

**Fig. 4:**
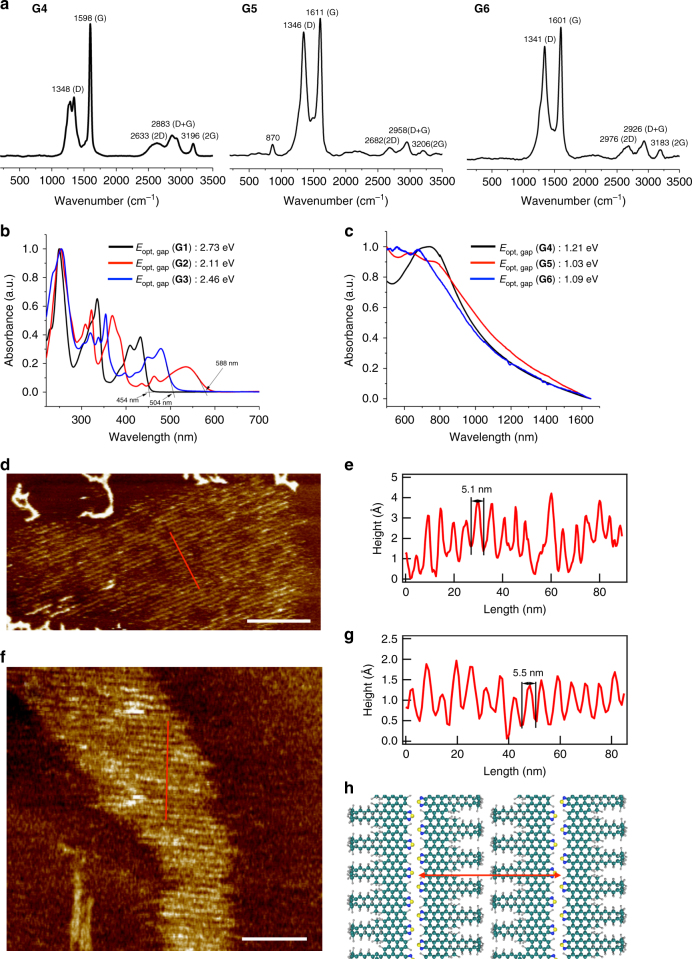
Characterization of nanographenes and GNRs. **a** Raman spectra of **G4–G6**. **b** UV–vis spectra of **G1–G3** in THF solution. **c** UV–vis-NIR spectra of **G4–G6** in THF suspension. **d** AFM image of **G5** on HOPG, scale bar: 70 nm. **e** A cross-sectional profile of the image (along the red line in **d**). **f** AFM image of **G6** on HOPG, scale bar: 50 nm. **g** A cross-sectional profile of the image (along the red line in **f**). **h** Molecular model of **G5**. Green, carbon; blue, nitrogen; yellow, sulfur; gray, hydrogen. *E*_opt, gap_: optical band-gap energy

FTIR analysis on **G4**–**G6** showed that, compared to their polymer precursors, the weak signals from free rotation of phenyl groups around 4052 cm^−1^ and a triad of peaks (3082, 3052, 3025 cm^−1^ for **P1**; 3083, 3049, 3025 cm^−1^ for **P2**; 3083, 3051, 3026 cm^−1^ for **P3**) from the aryl C–H stretching vibrations were diminished (Supplementary Fig. 9), indicating successful cyclodehydrogenation. Raman spectroscopy of **G4**–**G6** showed two intense peaks around 1340 and 1600 cm^−1^, assigned to D and G bands of graphitic materials, respectively (Fig. 4a). Edge-functionalized **G5** and **G6** exhibited ratios of the D band intensity to G band intensity (*I*_D_/*I*_G_) that were larger than observed for **G4**, likely due to their extended edge areas (Supplementary Fig. 10)^36^. Three second-order bands (2D, D + G and 2 G) were also detected for all aGNRs. The bimodal D band and the significantly broadened 2D band in **G4** (as compared with the *N* = 9 aGNR prepared previously^28^) suggested stronger aggregation via a π–π stacking interaction, probably owing to the alkyl chains unsymmetrically placed on one side edge (vide infra)^37^. A weak but sharp peak at 870 cm^−1^ was observed for **G5**, which is attributed to the N–S bond vibrations in the benzothiadiazole moiety^38, 39^.

UV–Vis–NIR spectroscopy and cyclic voltammetry were used to measure band-gaps (E_gap_s) of these nanographenes and GNRs (**G1**–**G6**). Nanographenes, **G1**–**G3**, only absorbed light in the UV and visible regions showing optical band-gaps of 2.73, 2.11, and 2.46 eV, respectively (Fig. 4b), which is consistent with the electrochemical band-gaps (2.77, 2.12, and 2.74 eV, respectively) measured by the cyclic voltammetry (Supplementary Fig. 14 and Supplementary Table 2). Not surprisingly, a narrower band-gap was observed with benzothiadiazole-derived **G2**, as benzothiadiazole is known to be a better electron acceptor than benzotriazole^33^. In contrast, polymeric GNRs **G4**–**G6** exhibit broad absorptions in the UV, visible, and even near IR (NIR) regions with blunt absorption onsets (Fig. 4c). Using the Tauc Method^40^, the optical band-gap of **G4** was determined as 1.21 eV (Supplementary Fig. 12a), which matches reasonably well with the theoretical value (1.11 eV)^5^. The benzotriazole-derived **G6** slightly reduced the band-gap to 1.09 eV, and the benzothiadiazole-derived **G5** shows a narrower optical band-gap at 1.03 eV (Supplementary Fig. 12), suggesting that the benzothiadiazole moiety was more effective in narrowing the band-gaps of GNRs. The electrochemical band-gaps of **G4–G6** are 1.46, 1.25, and 1.31 eV, respectively (Supplementary Table 2), following the same order as their optical band-gaps. Notably, the trend of band-gap transitions among these GNRs (**G4–G6**) parallels those in donor–acceptor alternating conjugated polymers (see Supplementary Fig. 13 as an example)^31–33^, which is expected to provide important implications for band-gap engineering of GNRs.

Detailed information on the structure of these GNRs was obtained by AFM. Figure 4d shows an AFM image of **G5** deposited on highly oriented pyrolytic graphite (HOPG). GNRs self-assembled into small 2D domains consisting of highly ordered stripes. The average periodicity of these stripes is 4.8 ± 0.4 nm (±: the standard deviation, the number of replicates (*r*) = 27), as shown by a line profile (Fig. 4e) across the domain in the zoom-in AFM image. The interstripe distance is approximately twice the width of **G5**, indicating the formation of a head-to-head, tail-to-tail sub-structure as depicted in Fig. 4h. Formation of such a dimeric sub-structure is reasonable, as the aliphatic chains are only located on one side of the ribbon. Similarly, **G6** forms highly ordered stripes with the average periodicity of 5.5 ± 0.4 nm (*r* = 33) on HOPG as shown in Fig. 4f, g. The slightly larger average periodicity of **G6** is presumably due to the butyl side-chains on the benzotriazole moieties. Note that the AFM images show that aGNRs line up along the same stripe, therefore the apparent length of each stripe can extend to a few hundred nanometers. To estimate the lengths of these aGNRs, we measured the lengths of individual GNRs with distinct ends. The statistical results obtained from AFM images are 34 ± 14 nm (*r* = 111) for **G5** and 48 ± 11 nm (*r* = 69) for **G6**, which are slightly higher than the estimated values from *M*_*n*_s of the corresponding polymer precursors (22 nm for **G5** and 27 nm for **G6**, respectively). These values also match with the lengths estimated from the STM images (~26 nm for **G4**, 26–38 nm **G5**, 20–47 nm for **G6**, respectively, Supplementary Fig. 18).

In order to reduce undesired aggregations and increase solubility of the pristine *N* = 6 aGNR **G4**, a short polymer precursor (*M*_*n*_ = 6.5 kDa, *Đ* *=* 1.32) with much bulkier side chains (nonyltetradecyl groups) was employed to prepare aGNR **G4′** (Supplementary Fig. 16b) in a similar fashion as **G4**—the AFM image of the pristine aGNR **G4** suggests strong aggregation of the ribbons (Supplementary Fig. 15), which makes it difficult to obtain detailed structural information. Interestingly, Raman spectrum of **G4′** contains two additional low-frequency peaks at 300 and 463 cm^−1^ (Supplementary Fig. 10a), which were not detected in the spectrum of **G4**. We attribute the strong peak at 463 cm^−1^ to the radial breathing-like mode (RBLM)^41–43^, in excellent agreement with the theoretically calculated value of 465.7 cm^−1^ for the pristine *N* = 6 aGNR^41^. In addition, compared with **G4**, **G4′** shows a sharper D band and much higher solubility, indicating that pristine aGNR **G4′** is less aggregated due to bulkier side chains^37^. An AFM image of aGNR **G4′** on HOPG shown in Supplementary Fig. 16a illustrates a similar structure with striped domains. A line profile across the boundary shows a height of 0.35 nm and an interstripe separation of 5.3 ± 0.3 nm (*r* = 37) (Supplementary Fig. 16c). The height is in good agreement with the interlayer distance of graphite, indicating the formation of organized monolayers. The periodicity of stripes is about twice as long as the width of **G4′** (Supplementary Fig. 16d), suggesting a similar head-to-head, tail-to-tail pattern as **G5** and **G6**.

### Unique features about the unsymmetrical heterocyclic edges

**Fig. 5:**
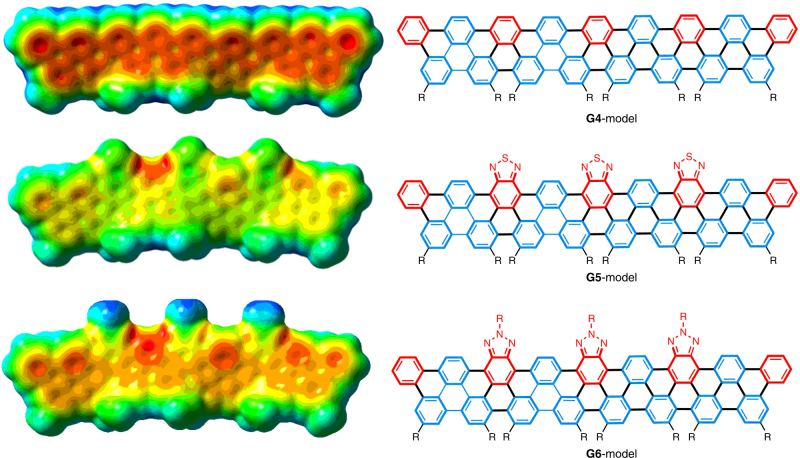
DFT calculation. Electron density of **G4**–**G6**-**models** (trimer models). Areas of higher electron density are colored red, and areas of lower electron density are colored blue

To better understand the electronic properties of the edge-functionalized GNRs in comparison with pristine ones, the electron density (ground-state geometries) of model oligomeric **G4**–**G6** were calculated by density functional theory (DFT) using the B3LYP functional and 6–31 G(d) basis set (Fig. 5)^44^. As expected, the electron-density distribution of the pristine **G4-model** shares a typical pattern with regular GNRs that exhibit high-electron density along the inner part of the polymer^15–19^. In contrast, the π-electron density of the **G5-** and **G6-models** significantly polarizes the molecules away from the polymer backbone and is largely located on the peripheral edge that contains heteroarenes, which is likely caused by the unsymmetrical edges of the doped ribbons, as well as the strong electron-withdrawing property of the benzothiadiazole and benzotriazole moieties.

**Fig. 6:**
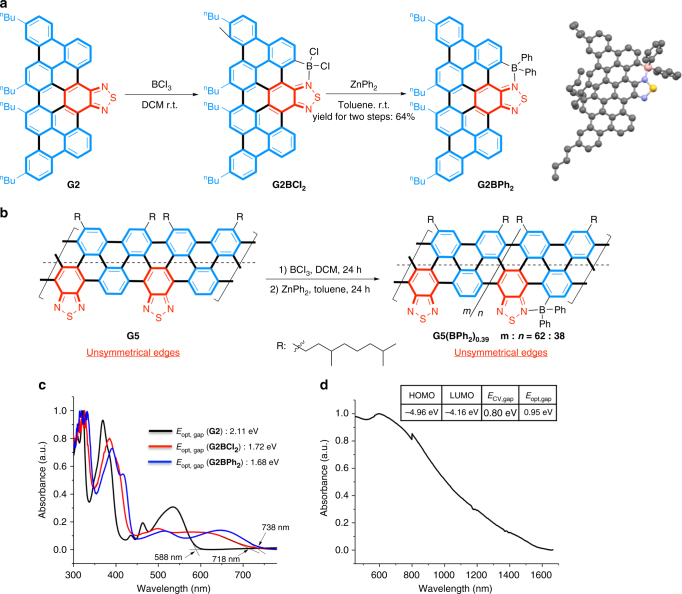
Post-functionalization through unsymmetrical heterocyclic edges. **a** C–H borylation of **G2** (and its crystal structure) and. **b** C–H borylation of **G5**. **c** UV–vis spectra of **G2**, **G2BCl**_**2**_, and **G2BPh**_**2**_ in THF. **d** UV–vis–NIR spectrum of **G5(BPh**_**2**_**)**_**0.38**_ in THF suspension and HOMO/LUMO levels obtained by cyclic voltammogram of **G5(BPh**_**2**_**)**_**0.38**_ (inset table)

One distinct merit of the GNRs with unsymmetrical edges arises from the Lewis basicity of the heteroarenes, which offers a unique way to manipulate the band-gap and energy level of the frontier molecular orbitals of the material. For example, the *sp*^2^-hybrized nitrogen in benzothiadiazole is known to direct electrophilic C–H borylation at an adjacent arene^45, 46^. We envisaged that such an approach could be employed to enable post-functionalization of benzothiadiazole-containing **G2** and **G5** through additional “boron-doping”, which should consequently reduce the band-gap and LUMO energy of the material. Indeed, C–H borylation of nanographene **G2** was successful upon simple treatment with BCl_3_; subsequently addition of ZnPh_2_ afforded more stable **G2BPh**_**2**_ (Fig. 6a), which was fully characterized by ^1^H/^13^C NMR, FTIR spectroscopy, X-ray crystallography and MALDI MS (Supplementary Fig. 4c). In contrast, **G1**, the nanographene without heterocyclic edges, only formed an intermolecular charge transfer complex with BCl_3,_ and this non-covalently bonded complex easily dissociated under vacuo or in the presence of a Lewis base (Supplementary Fig. 19). Similarly, borylation of GNR **G5** was conducted with a similar protocol (Fig. 6b). ICP-MS showed that the boron/sulfur (B:S) ratio in the resulting product [**G5(BPh**_**2**_**)**_***n***_] was 0.38:1.00, indicating that the borylation efficacy was about 38% (B:S = 1:1 when 100%). Unsurprisingly, the Raman spectrum of **G5(BPh**_**2**_**)**_**0.38**_ exhibited higher *I*_D_/*I*_G_ than **G5** (Supplementary Fig. 10), supporting the presence of enlarged edge regions caused by the formation of boracycles^36^.

UV–vis–NIR analysis on **G2BPh**_**2**_ shows a significantly red-shifted absorption onset compared to **G2**, because the electron-deficient boron moiety further enlarged conjugation width and intramolecular charge transfer (Fig. 6c). The optical and electrochemical band-gap of **G2BPh**_**2**_ was 1.68 eV and 1.81 eV, respectively (Supplementary Table 2). Likewise, G**5(BPh**_**2**_**)**_**0.38**_ possessed an optical band-gap of 0.95 eV (via the Tauc plot) and an electrochemical band-gap of 0.80 eV, which is narrower than those of **G5** (Fig. 6d and Supplementary Fig. 12). This result illustrates that the unsymmetrical edge enables convenient post-functionalization, thereby allowing further band-gap engineering.

In summary, a modular approach to solution-phase synthesis of *N* = 6 aGNRs has been developed, offering an efficient and practical entry to both pristine and edge-functionalized materials. The strategy holds the advantage of flexibility of choosing monomer components, as well as the simple, predictable and reliable synthetic routes. It is expected that a diverse range of edge-functionalized aGNR analogues would be rapidly prepared using this modular approach, which should ease band-gap engineering of aGNR-type materials. In addition, the unsymmetrical heterocycle edges of these GNRs open the door for additional band-gap engineering via simple post-functionalization, e.g., directed C–H borylation. It can be envisioned that by changing the electronic properties of the aryl groups on the boron, additional fine-tuning of bandgaps would become possible. The work on this topic is underway in our laboratories.

## Methods

### Synthesis

Experimental details and characterization data (^1^H NMR, ^13^C NMR, HRMS, etc.) for all molecules can be found in Supplementary Methods.

### Characterization and imaging of nanographenes and graphene nanoribbons

Experimental details for spectroscopic analyses and microscopic tools can be found in Supplementary Methods.

### Data availability

Crystallographic data in this study were deposited at the Cambridge Crystallographic Data Centre with the accession code (CCDC 1524981 (**G1**), 1524982 (**G2**), 1530954 (**G3**), and 1822453 (**G2BPh**_**2**_)). The authors declare that all other data supporting the findings of this study are available from the article and its Supplementary Information files or available from the authors upon reasonable request.

## Electronic supplementary material

Supplementary Information(PDF 5458 kb)

Peer Review File(PDF 245 kb)
