# Supplementary material for: A modular synthetic approach for band-gap engineering of armchair graphene nanoribbons
Source: Nat Commun. 2018 Apr 27;9:1687. doi: 10.1038/s41467-018-03747-2 (PMC5924368; doi:10.1038/s41467-018-03747-2)
Supplement: Supplementary file 1 — Supplementary Information(PDF 5458 kb) [file 41467_2018_3747_MOESM1_ESM.pdf]

A Modular Synthetic Approach for Band-Gap Engineering of Armchair Graphene Nanoribbons

Li *et al.*

Table of Contents

1. Supplementary Figures

NMR Spectra of the crude reaction mixture for the cyclodehydrogenation step

SEC traces

MALDI TOF analysis

FTIR spectra

Supplementary Raman spectra and analysis

X-ray photoelectron spectroscopy analysis

Supplementary UV/vis/NIR spectra and analysis

Cyclic voltammograms and analysis

Additional AFM characterization and STM images

Control experiment of the post-borylation of G1

2. Supplementary Tables

Polymerization table

Bandgap table

3. Supplementary Methods

General

Synthesis of nanographenes

Synthesis of polymer precursors

Synthesis of graphene nanoribbons

X-ray data

4. Spectra (Link)

5. References (Link)

Supplementary Figures

a)

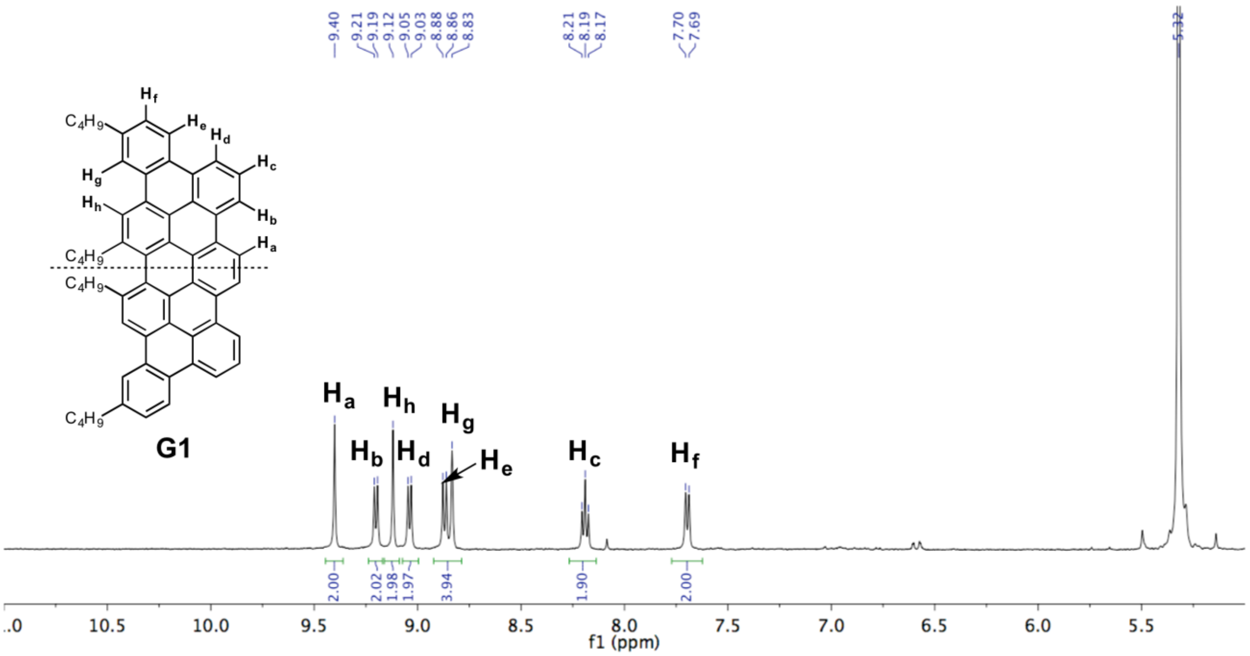

b)

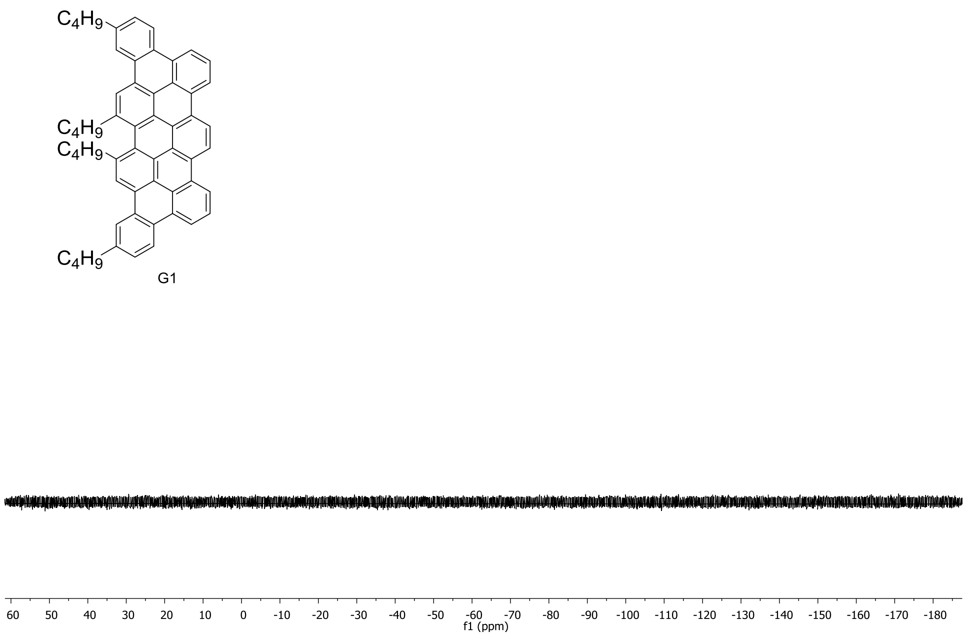

Supplementary Figure 1. NMR Spectra of the crude reaction mixture for the cyclodehydrogenation step (a) ^{1}H NMR spectrum of the *crude* reaction mixture of **G1**. (b) ^{19}F NMR spectrum of the *crude* reaction mixture of **G1**.

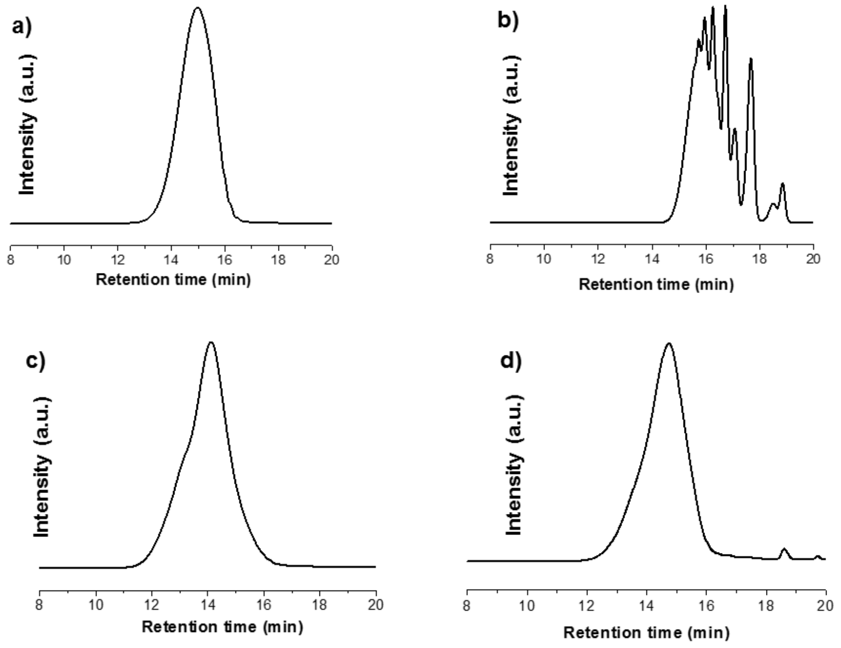

Supplementary Figure 2. Representative SEC profiles of polymer precursors in Supplementary Table 1 a) P1 (entry 4), **b) P1'** (entry 5), **c) P2** (entry 9) and **d) P3** (entry 15) (THF, 1.0 mL/min, UV detector: 260 nm, Polystyrene Standard).

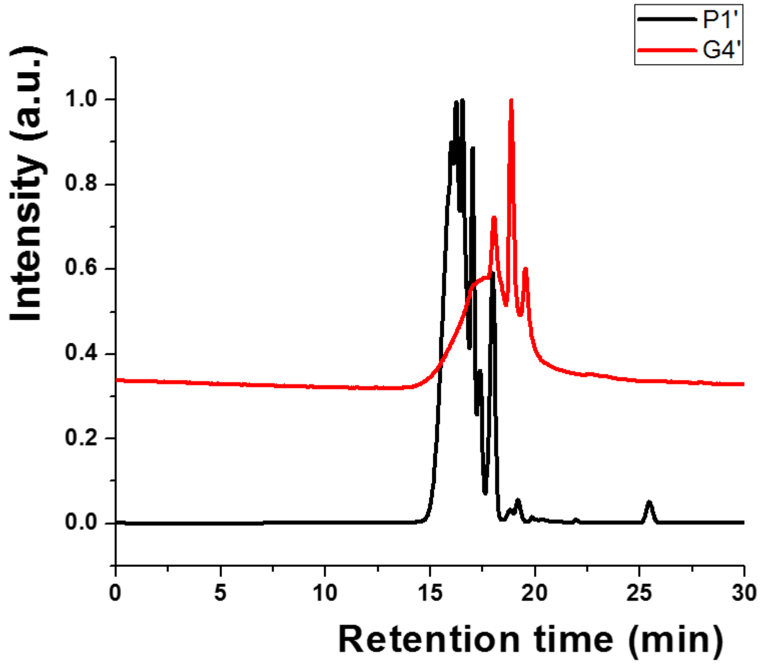

Supplementary Figure 3. SEC traces of **P1'** (blank, entry 5 in Supplementary Table 1) and **G4'**(red). The SEC traces of **P1'** and **G4'** show similar retention time (M_n: 8.5 KDa, Đ: 1.3 for **P1'** and M_n: 8.1 KDa, Đ: 2.9 for **G4'**), indicating that the inter-chain cross-linking is unlikely.

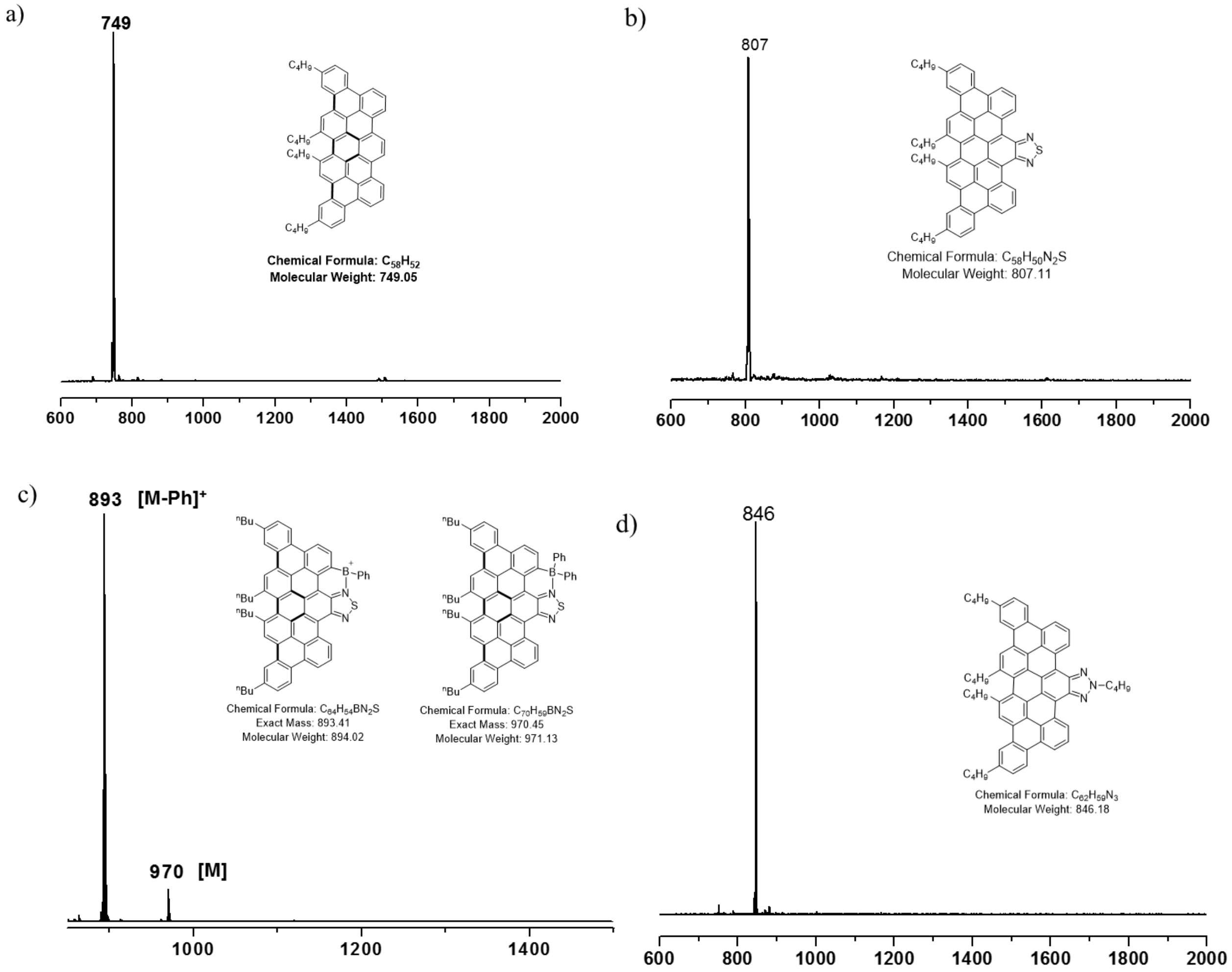

Supplementary Figure 4. MALDI TOF MS spectra of a) **G1**, b) **G2**, c) **G2BPh$_2$**, and d) **G3** (Reflection mode, matrix: DCTB).

The MALDI TOF analyses of polymer precursors show intervals of 584 for **P1**, 643 for **P2** and 682 for **P3**, which are in agreement with corresponding exact mass of one repeating unit 584.45 for **P1**, 643.01 for **P2** and 682.05 for **P3**. Phenyl end groups were detected as high intensive peaks for **P1** and **P2**, indicating that both bromobenzene and Phenyl boronic acid serve as end cappers, whereas hydrogen end groups were detected in the case of **P3**.

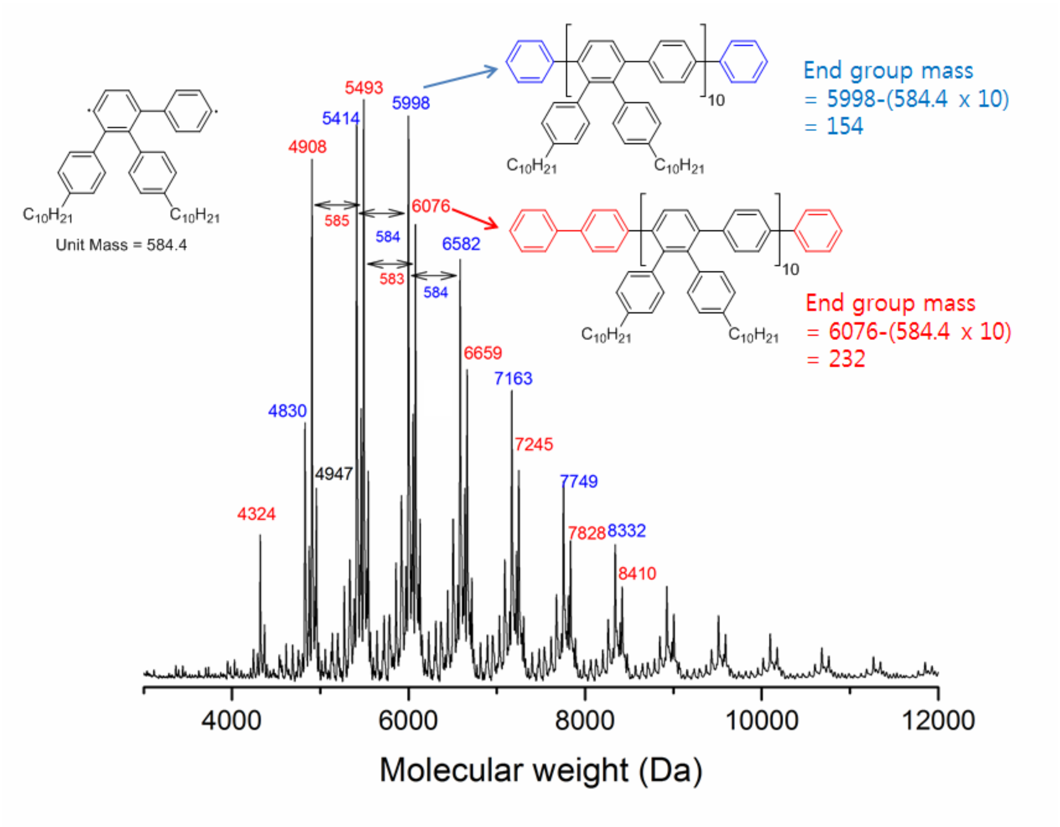

Supplementary Figure 5. MALDI TOF mass spectrum of **P1** (linear mode, matrix: DCTB).

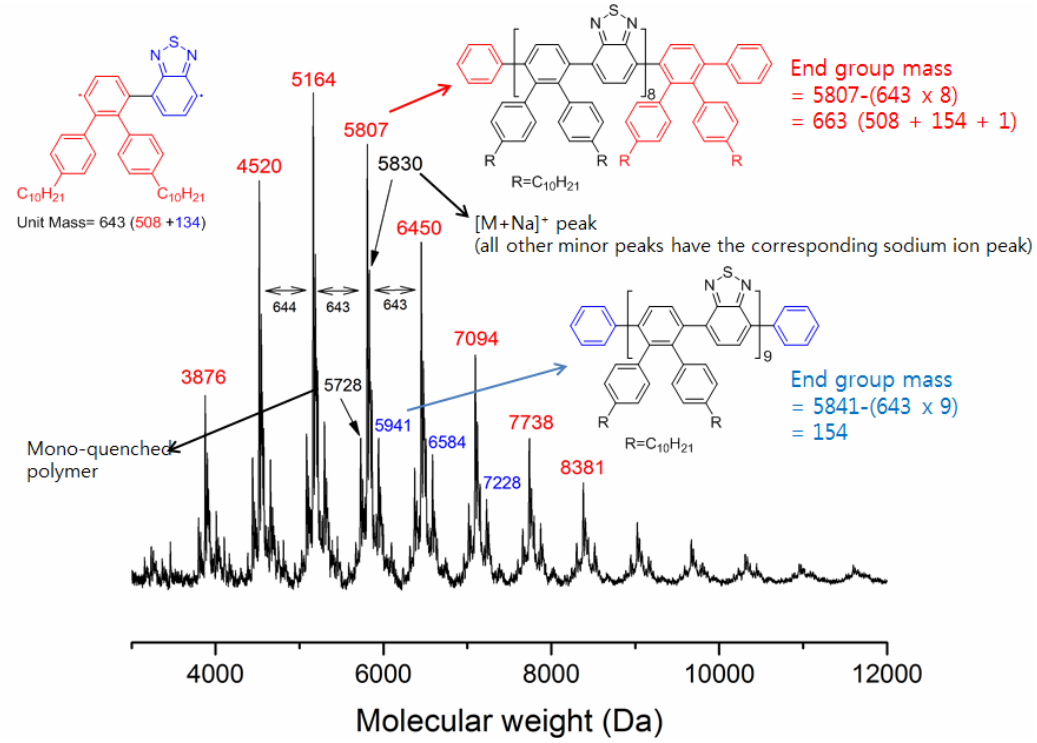

Supplementary Figure 6. MALDI TOF mass spectrum of **P2** (linear mode, matrix: DCTB).

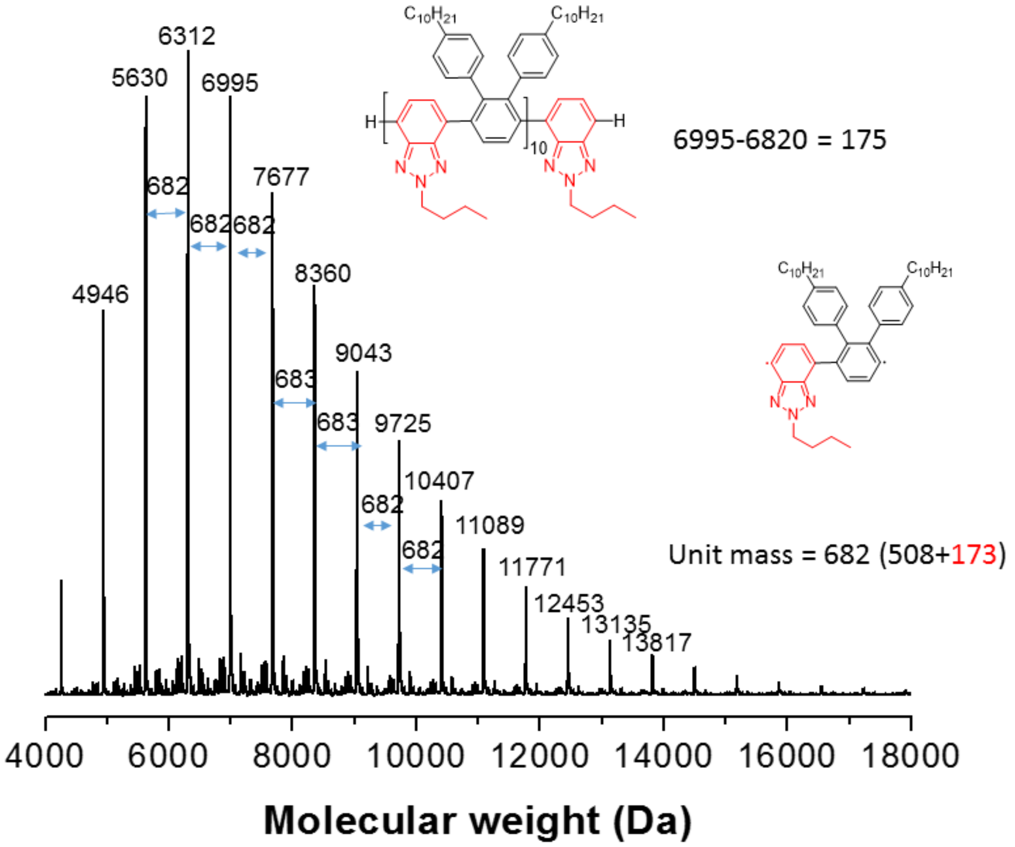

Supplementary Figure 7. MALDI TOF mass spectrum of **P3** (linear mode, matrix: DCTB).

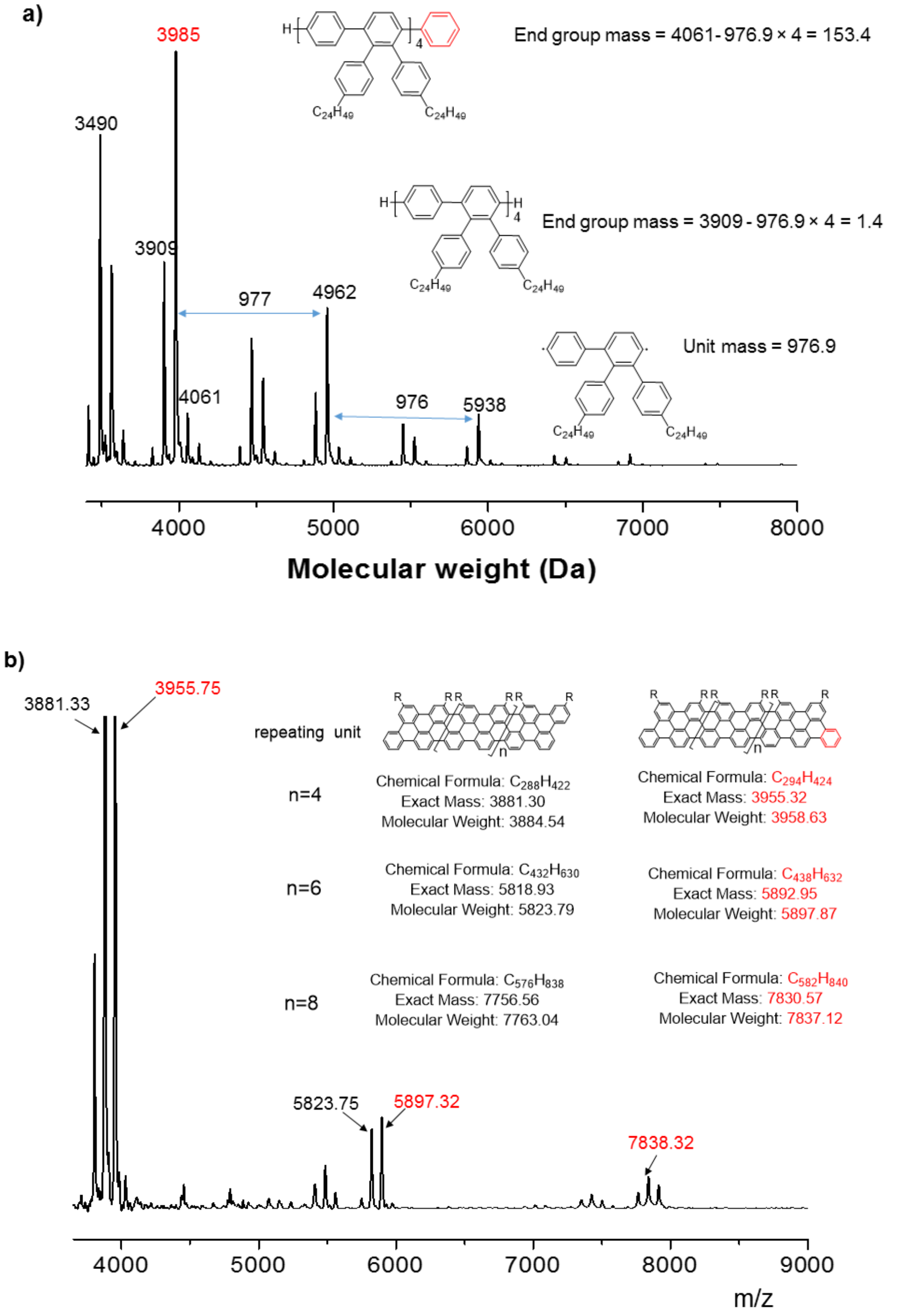

Supplementary Figure 8. MALDI-TOF mass spectra of a) **P1'** and b) **G4'**. (linear mode, matrix: DCTB). The mass values are in agreement with the expected ones, confirming the successful formation of N = 6 aGNR.

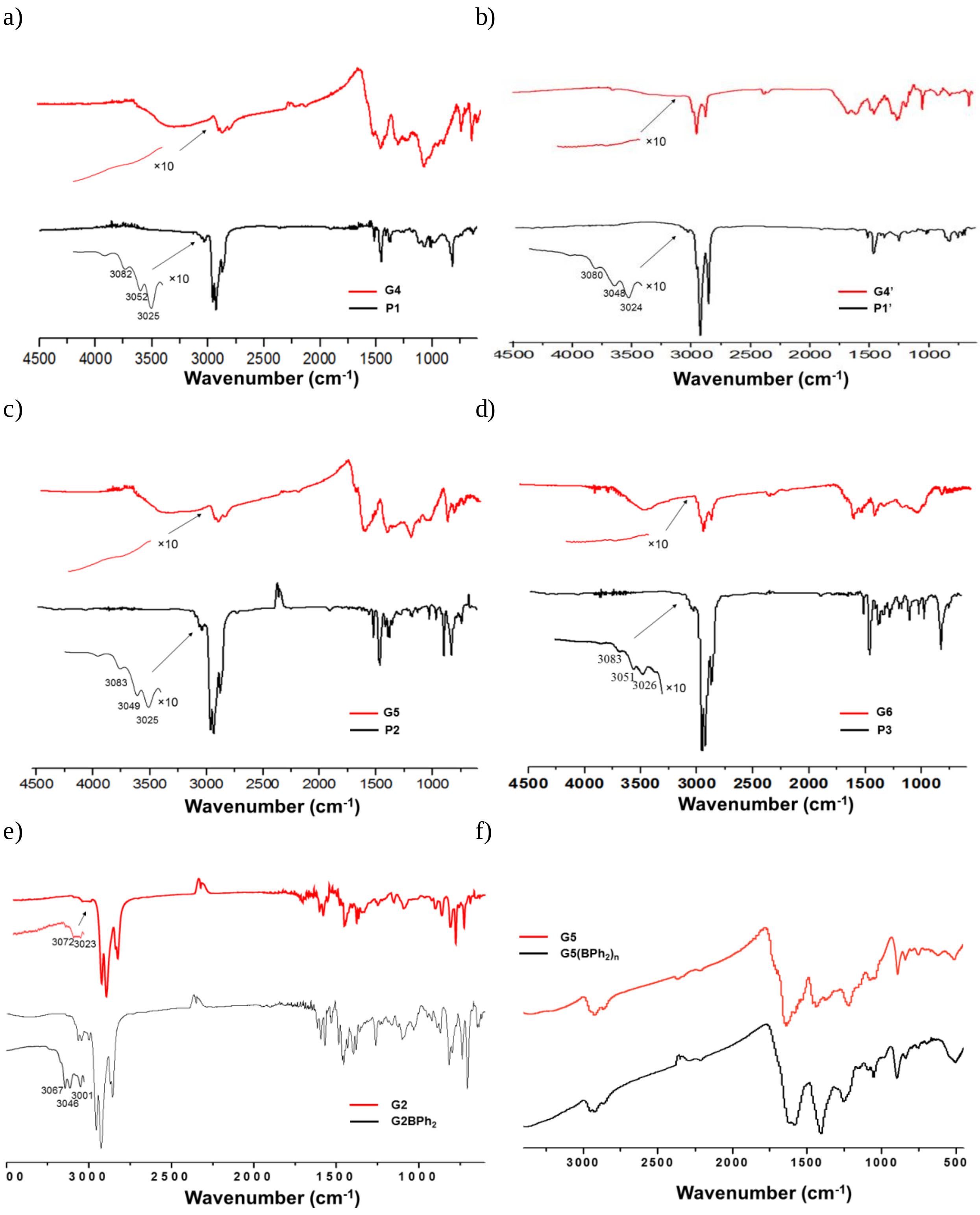

Supplementary Figure 9. FTIR spectra of **a) G4** (red) and **P1** (black), **b) G4'** (red) and **P1'** (black), **c) G5** (red) and **P2** (black), **d) G6** (red) and **P3** (black line), **e) G2** (red) and **G2BPh$_2$** (black), and **f) G5** (red) and **G5(BPh$_2$)$_{0.38}$** (black).

a) **G4'**

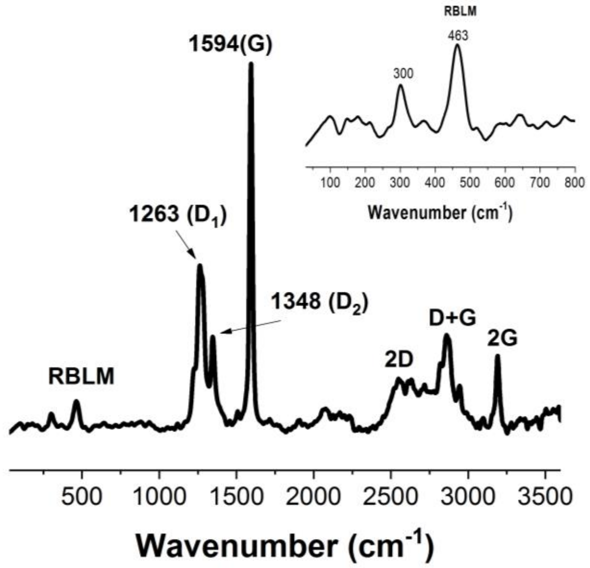

b) **G5(BPh$_2$)$_{0.38}$**

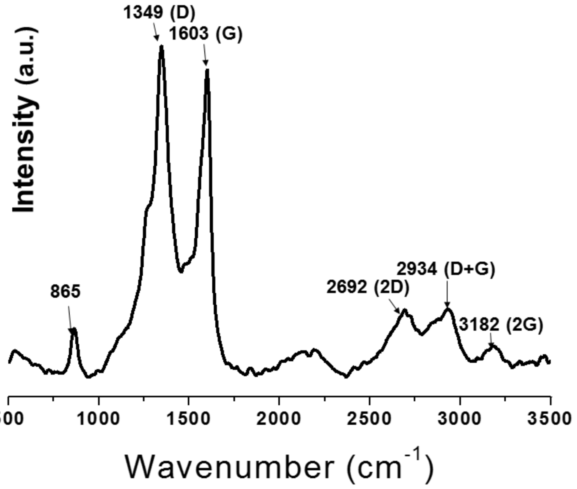

c)

	I_D/I_G
**G4**	0.44
**G4'**	0.45
**G5**	0.90
**G6**	0.85
**G5(BPh$_2$)$_{0.38}$**	1.09

Supplementary Figure 10. Raman spectra of **a) G4'**(inset: the magnified low-frequency region), **b) G5(BPh$_2$)$_{0.38}$**, and **c)** I_D/I_G ratios for all synthesized aGNRs.

[Discussion]: **G4'** minimized aggregation due to bulkier side-chains. Thus, the sharp D_1 band as well as the radial-breathing-like mode, coming from the edge of the single-layer graphene nanoribbon, was observed.

The intensity ratio between G band and disorder/defect-induced D band is for the "defect" density of graphene. Since the edge is considered one of the "defects" of graphene, GNRs with different edge structures therefore have different I_D/I_G. We observed the trend of **G4~G4'<G5~G6<G5(BPh$_2$)$_{0.38}$**, in an agreement of the expectation, based on the literature,[1] that the GNR with a larger area of edge results in higher I_D/I_G.

a)

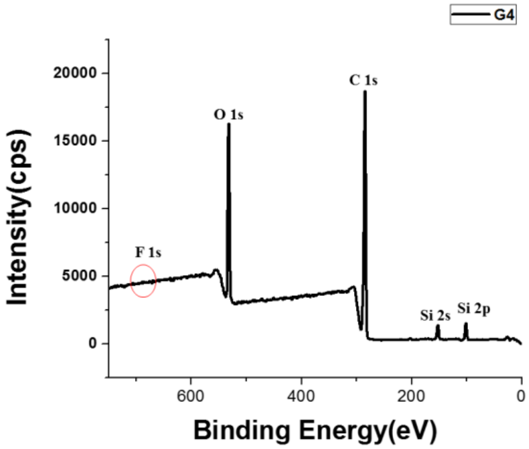

b)

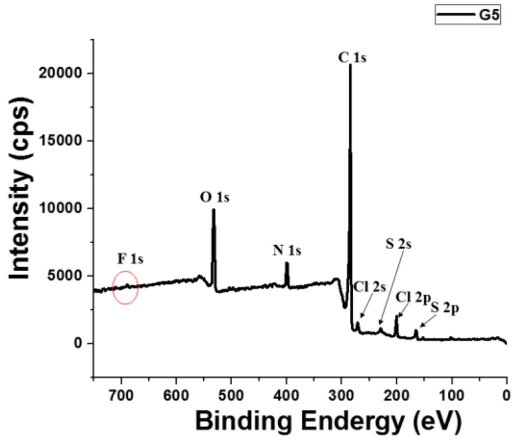

c)

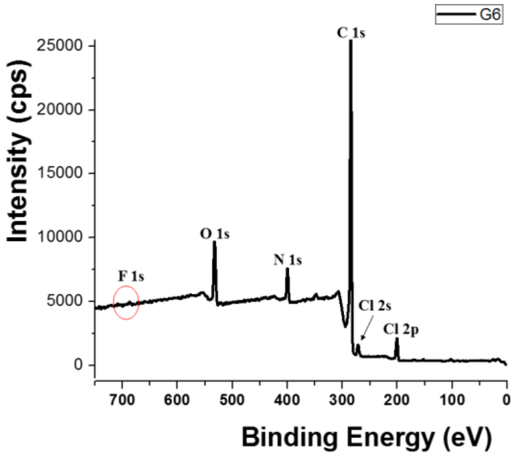

Supplementary Figure 11. XPS spectra of (a) **G4**, (b) **G5**, and (c) **G6**. XPS analysis ruled out the significant incorporation of fluorine atoms to GNRs by TsOH during the cyclodehydrogenation.

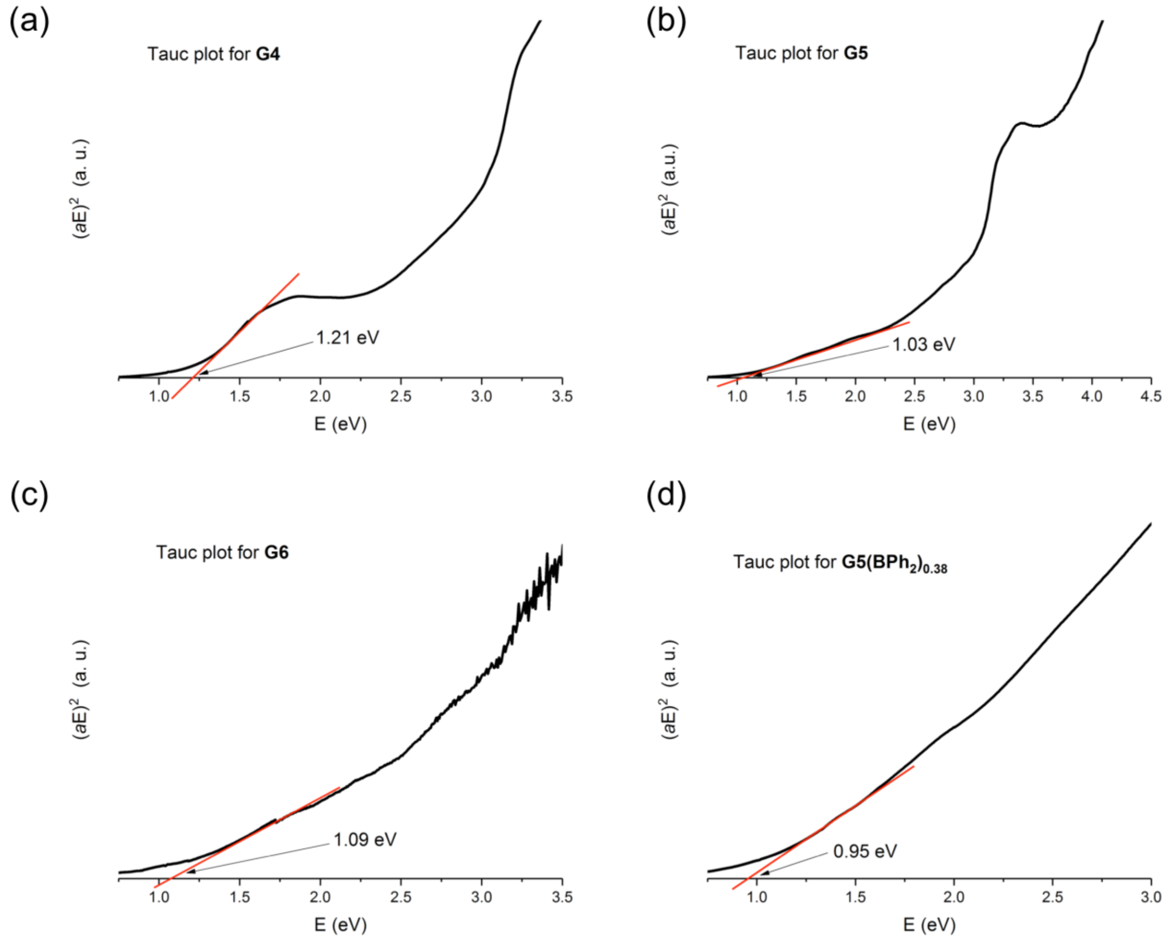

	G4	G5	G6	G5(BPh$_2$)$_{0.38}$
optical E_{gap} (eV)	1.21	1.03	1.09	0.95

Supplementary Figure 12. Tauc plots of (a) **G4**, (b) **G5**, (c) **G6**, and (d) **G5(BPh$_2$)$_{0.38}$**.

Since the absorption onsets of these aGNRs were unclear from their UV/vis/NIR spectra, it would be ambiguous to determine the optical band-gaps directly. Thus, we calculated the optical band-gaps of aGNRs with the help of the Tauc Method, the equation of which is $[(\alpha h\upsilon)^2=A(h\upsilon-E_{gap})]$,[2] as it has been previously applied in the band-gap measurement of GNRs.[3]

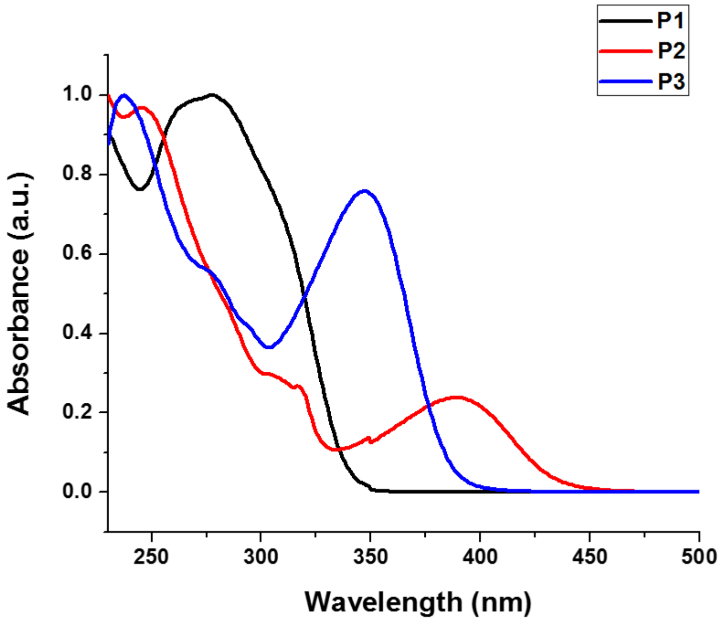

Supplementary Figure 13. UV-vis spectra of polymer precursors **P1** (black) and **P2** (red) and **P3** (blue) in THF.

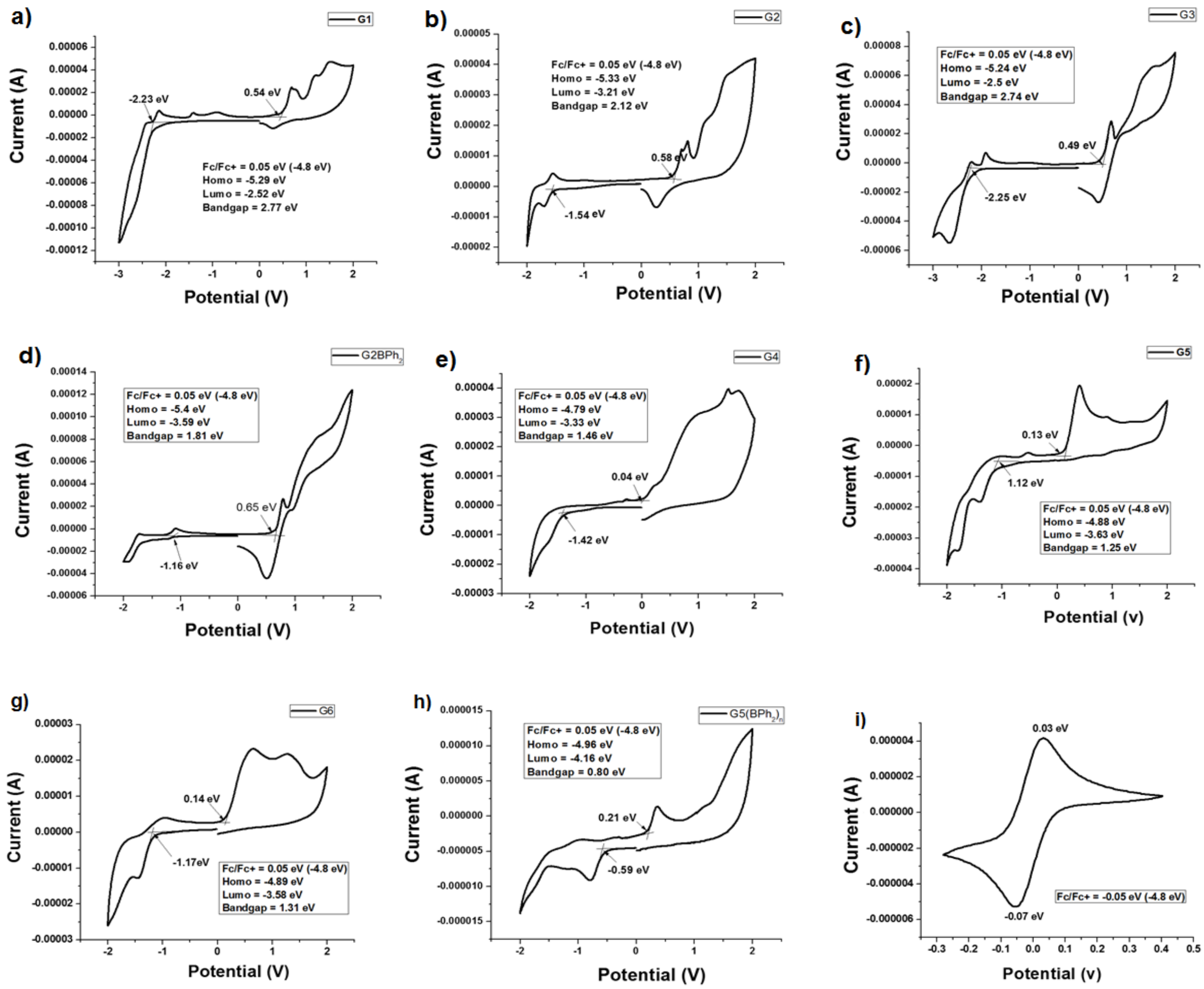

Supplementary Figure 14. Cyclic voltammograms of **a) G1**, **b) G2**, **c) G3**, **d) G2BPh$_2$**, **e) G4**, **f) G5**, **g) G6**, **h) G5(BPh$_2$)$_{0.38}$**, and **i)** ferrocene. HOMO/LUMO were estimated from the oxidation potential of ferrocene (Fc$^+$/Fc= -4.8 eV).

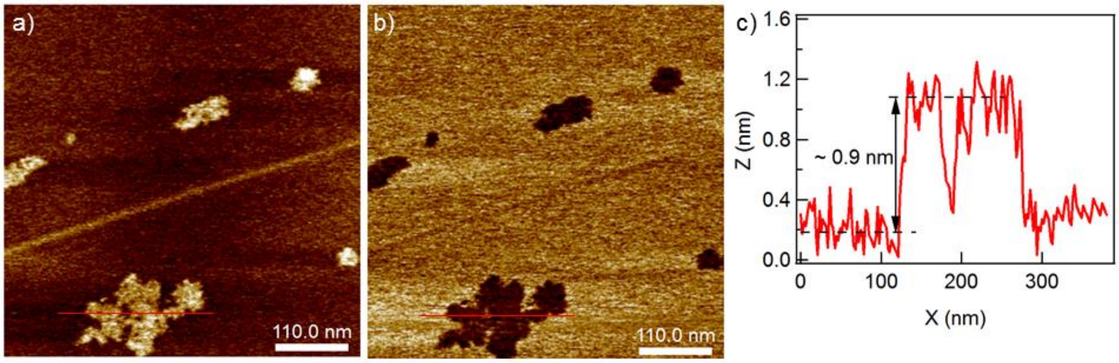

Supplementary Figure 15. a) AFM height image and b) AFM adhesion image of **G4** on HOPG. c) the cross-sectional profile (along the red line in panel a).

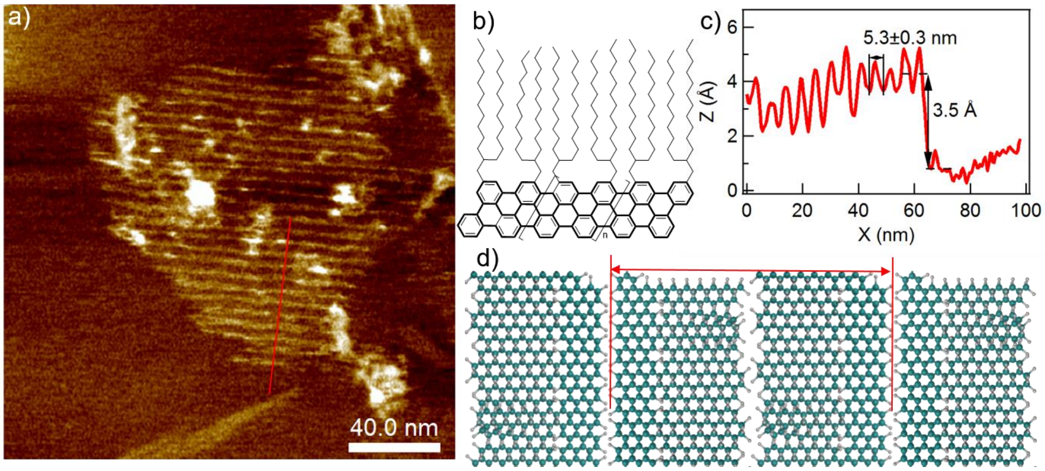

Supplementary Figure 16. a) AFM image of **G4’** on HOPG. **b)** Molecular structure of **G4’**. **c)** the cross-sectional profile (along the red line in panel **a)**. **d)** Molecular model of **G4’**. Green, carbon; grey, hydrogen.

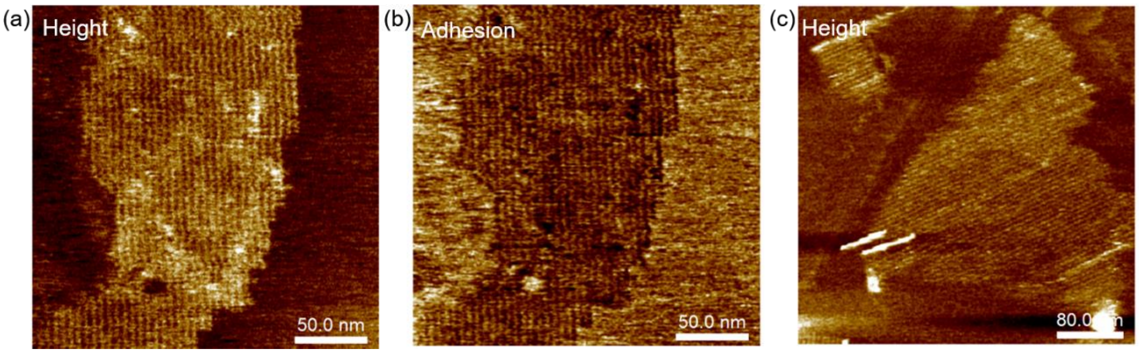

Supplementary Figure 17. a) AFM height image and b) AFM adhesion image of **G4’** on HOPG. c) AFM height image of **G6** on HOPG.

G4 aggregates into islands with various sizes, as shown in Supplementary Figure 15a. A line profile (along the red line in Supplementary Figure 15a) illustrates a height of 0.9 nm across the terraces, which is higher than the height of single-layer graphene on HOPG.

The clear contrast between GNRs and HOPG is seen in the adhesion image (Supplementary Figure 15b & 17b), indicating that what we observe is not HOPG itself. Supplementary Figure 17c shows a large-scale height image of **G6** containing two domains with different sizes. In the large domain, the longitudinal dimension changes across the stripes, demonstrating that stripes consist of different numbers of GNRs with various lengths.

STM characterization of GNR G4, G5 and G6

In contrast to the well-ordered patterns on HOPG, isolated GNRs were found absorbing on Au (111) surface. Length of GNRs is ~ 26 nm, in good agreement with the calculated average value of 28 nm based on M_n. Supplementary Figure 18b shows a STM image of GNR **G5** with various lengths ranging from 26 nm to 38nm. As depicted in Supplementary Figure 18c, several GNR **G6** arrange themselves in parallel on Au and the long GNR found in the image is ~47 nm. Since all measurements were operated at room temperature, it has an uncertainly of ± 10% in the measured lengths due to thermal drift.

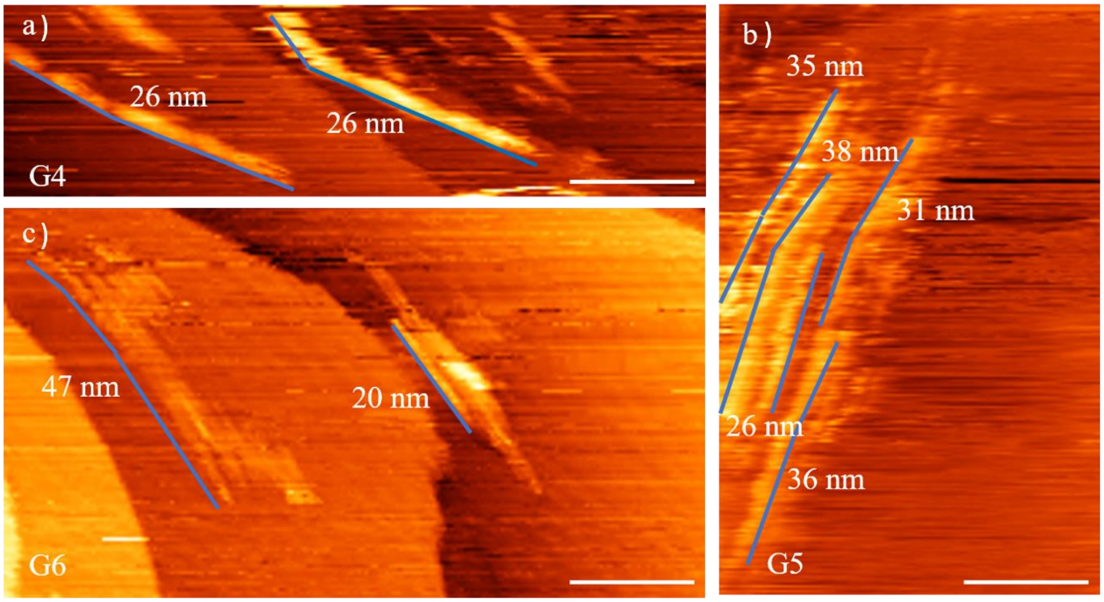

Supplementary Figure 18. a) STM image of **G4** on Au (111), image size: 80 x 22 nm. (I = 40 pA, V = 1.5 V). b) STM image of **G5** on Au (111), image size: 60 x 95 nm. (I = 40 pA, V = 1.5 V). c) STM image of **G6** on Au (111), image size: 133 x 82 nm. (I = 40 pA, V = 1.5 V).

Control experiment of the post-borylation of G1 with BCl_3.

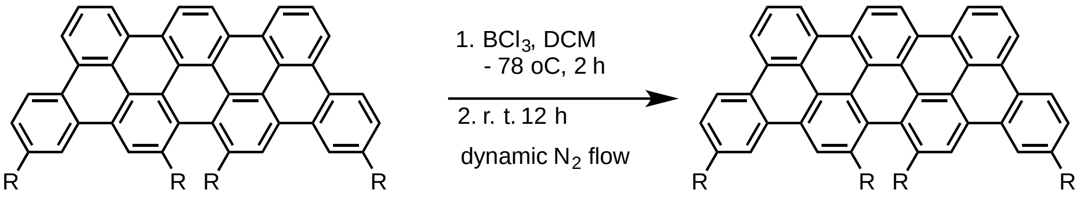

R= ^{n}Bu

G1

a)

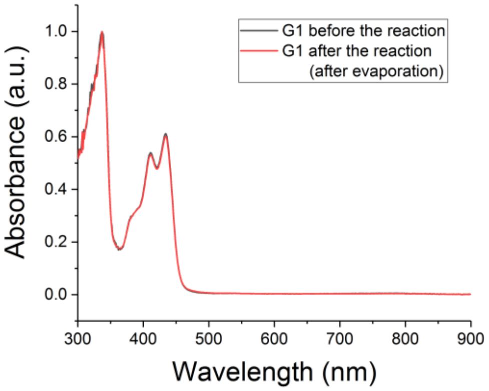

b)

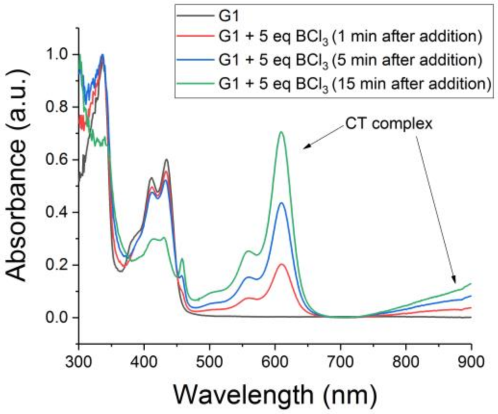

c)

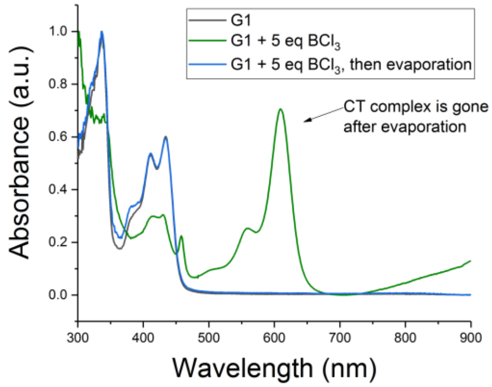

d)

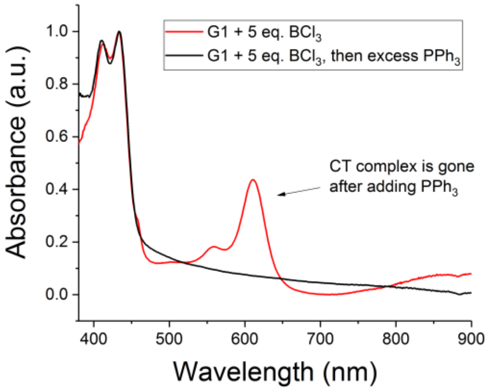

Supplementary Figure 19. UV-vis study of the reaction between **G1** and BCl_3. Regarding the reaction between **G1** and BCl_3, no change was observed from the UV/vis spectra after workup and purification (see part a). This shows that covalently linked complexes were not formed. What happens in solution during the reaction is that the intermolecular CT complex of **G1** (donor) with BCl_3 (acceptor) was formed (see part b), which is readily dissociated by the vacuum evaporation (see part c) or the addition of Lewis bases (see part d).

Supplementary Tables

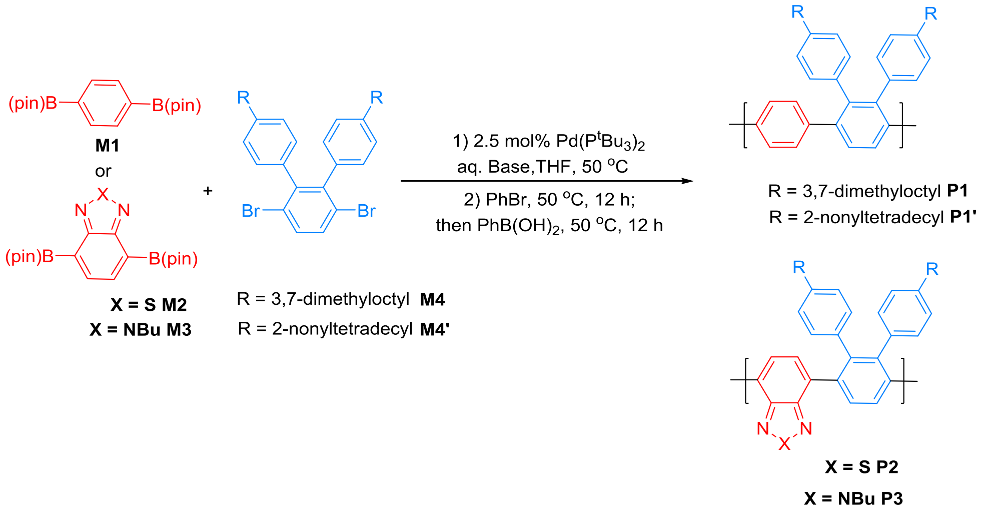

Supplementary Table 1. Polymerization table

Entry	Monomer	Base	Time (h)	Solv.	Yield[a] (%)	Mn[b] (kDa)	Mw[b] (kDa)	Đ[b]	DP[c]	L(nm)[d]
1	**M1+M4**	3 M K_3PO_4	24	THF	96	21.5	44.9	2.09		
2					[73	28.9	49.4	1.71	50	42][e]
3	**M1+M4**	5 M K_3PO_4	12	THF	96	15.8	27.2	1.71		
4					[68	19.6	28.1	1.43	34	28][e]
5[f]	**M1+M4'**	3 M K_3PO_4	24	THF	74	6.5	8.5	1.32	7	6
6	**M2+M4**	3 M K_3PO_4	24	THF	97	36.8	80.2	2.18		
7					[95	37.8	80.1	2.12	59	49][e]
8	**M2+M4**	5 M K_3PO_4	12	THF	94	12.2	21.4	1.76		
9					[70	17.4	25.4	1.46	27	22][e]
10	**M2+M4**	3 M K_3PO_4	24	DCM	98	66.1	203.7	3.08	103	86
11	**M3+M4**	3 M K_3PO_4	24	THF	95	22.6	40.5	1.79		
12					[82	27.6	43.4	1.57	41	34][e]
13	**M3+M4**	5 M K_3PO_4	12	THF	87	21.5	46.0	2.14	32	27
14	**M3+M4**	3 M K_2CO_3	8	THF	82	17.5	34.9	2.00		
15					[66	21.6	37.4	1.73	32	27][e]

[a] Isolated yield; [b] Measured by THF SEC with PS standard; [c] Degree of the polymerization based on M_n of the polymer precursor. [d] The expected length of the corresponding aGNR based on M_n of the polymer precursor. [e] After Soxhlet extraction with boiling acetone; [f] 5 mol% Pd($^tBu_3P)_2$.

Supplementary Table 2. Optical and electrochemical band-gaps of nanographenes and aGNRs

	G1	***G2***	***G3***	***G2BPh$_2$***	***G4***	***G5***	***G6***	***G5(BPh$_2$)$_n$***
Homo (eV)	-5.29	-5.33	-5.24	-5.4	-4.79	-4.88	-4.89	-4.96
Lumo (eV)	-2.52	-3.21	-2.5	-3.59	-3.33	-3.63	-3.58	-4.16
CV Bandgap (eV)	2.77	2.12	2.74	1.81	1.46	1.25	1.31	0.80
UV Opt. gap(eV)	2.73	2.11	2.46	1.68	1.21	1.03	1.09	0.95

Supplementary Methods

General

All solvents, unless specifically mentioned, were dried by filtration through a Pure-Solv MD-5 Solvent Purification System (Innovative Technology). Tetrahydrofuran (THF) was distilled freshly over sodium-benzophenone and freeze-pump-thaw for four times before use. Dichloromethane (DCM) was distilled freshly over CaH_2 and freeze-pump-thaw for four times before use. ^{1}H NMR and ^{13}C NMR were recorded with a Varian Gemini (400 MHz, ^{1}H at 400 MHz, ^{13}C at 101 MHz) or Bruker Model DMX 500(500 MHz, ^{1}H at 500 MHz, ^{13}C at 126 MHz). Chemical shifts are reported in parts per million (ppm, δ), downfield from tetramethylsilane (TMS, δ=0.00 ppm) and are referenced to residual solvent ($CDCl_3$, δ = 7.26 ppm (^{1}H) and 77.00 ppm (^{13}C).

High-resolution mass spectra (HRMS) were obtained on a Karatos MS9 and are reported as m/z (relative intensity). Matrix-assisted laser desorption/ionization time-of-flight (MALDI-TOF) mass spectra were measured on a AB Voyager-DE PRO instrument or Bruker Ultraflextreme MALDI-Tof-Tof instrument in Reflection mode or linear mode, using trans-2-[3-(4-tert-butylphenyl)-2-methyl-2-propenylidene] malononitrile (DCTB) as matrix. The samples were digested with (68% v/v) nitric acid and analyzed for Boron and Sulfur concentrations by inductively coupled plasma-mass spectrometer (ICP-MS).

Size exclusion chromatography (SEC) for polymer molecular weight analysis (based on polystyrene standard) was carried out with Agilent 1260 Infinity system (VWD UV detector) and two 300 x 7.5 mm ResiPore GPC columns eluted with THF (HPLC grade, Fischer). Flow rate was 1.0 mL/min and the temperature of column was maintained at 35 ^{o}C.

Infrared spectra were recorded on a Nicolet 380 FTIR using neat thin film technique. Measurements with a scan number of 128 were recorded for each sample and the background was subtracted.

UV-vis-NIR spectra were measured on a Agilent Cary 5000 spectrophotometer. The baseline was corrected by subtracting a measurement of the cuvette filled with pure solvent used for the measurement. The samples were measured in THF solution/suspension.

Raman spectra were collected on powder samples using Horiba LabRAM HR Evolution NIR confocal Raman microscope. Excitation laser is at 532 nm. The power is below 1 mW.

AFM measurements were carried out with Bruker Dimension FastScan AFM in ambient conditions using peakforce tapping mode. To form films of GNRs, ~0.6 mg of GNR powder was dispersed in 10 mL of toluene, followed by heat and sonication cycles. The suspension was then centrifuged and the supernatant was sprayed on a freshly cleaved surface of HOPG at 50 ^{o}C while evaporation. All AFM images were analyzed using NanoScope Analysis.

STM measurements were acquired with a constant current mode in an Omicron ultrahigh-vacuum (UHV) STM at room temperature. Tungsten tips were prepared by electrochemical etching with 2 M NaOH solution and cleaned with running water. Ribbons were sprayed on a freshly cleaved surface of Au(111) by drop-casting the supernatant of GNR in THF/toluene. The sample bias (V_{bias}) and tunneling current (I_{set}) are indicated in figure caption.

Cyclic voltammetry (CV) measurements were carried out at room temperature on n AUTOLAB/PG-STAT12 system using a three-electrode system in a conventional one compartment three-electrode cell in propylene carbonate of tetra-*n*-butylammonium hexafluorophosphate solution. Thin film was prepared by drop-casting an aliquot of suspension in THF onto 1 mm diameter platinum electrode. Cyclic voltammogram was recorded using the **GNR**-coated working electrode and a reference electrode of $Ag/AgNO_3$ in acetonitrile (0.1 M) with a platinum wire as a counter electrode at a scan rate of 0.15 Vs^{-1}. The absolute energy level was calculated by using ferrocene/ferrocenium as an internal standard.

DFT calculation was conducted as following: the geometry was optimized in the gas phase with density functional theory (DFT) using B3LYP hybrid functional with basis set 6–31G(d). Quantum-chemical calculation was performed with the Gaussian09 package.[4] The

electron density map was visualized with the GaussView software, and the FMOs were visualized with the Avagadro software. Vibrational frequency calculations were performed to check that the stable structures had no imaginary frequency. For **G4 – G6 model**, all side chain groups were replaced with methyl groups in the calculations.

Synthesis of nanographenes

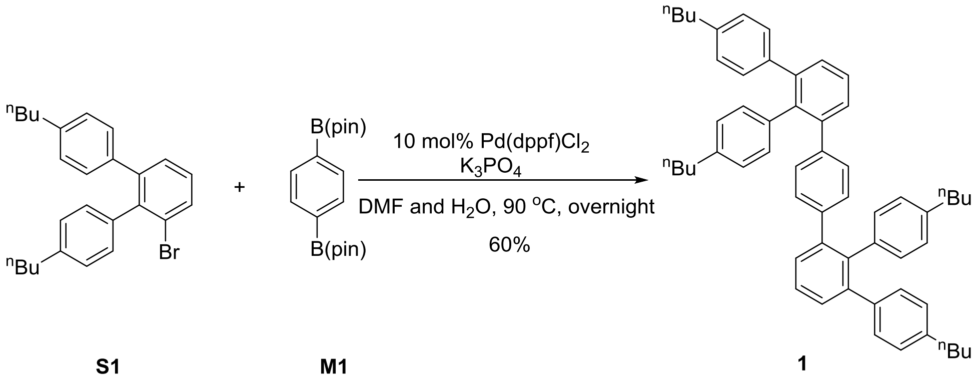

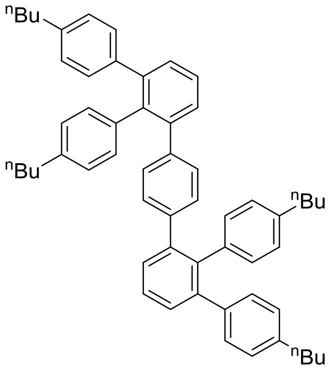

(**1**). **M1** (66 mg, 0.2 mmol), and **S1**[5] (210 mg, 0.5 mmol,), $K_3PO_4{\cdot}H_2O$ (230 mg, 1 mmol, 5 equiv.) and [1,1′-bis(diphenylphosphino) ferrocene] dichloropalladium(II) complex with dichloromethane (16 mg) were mixed in a solution of DMF (3 mL) and water (0.6 mL). The mixture was degassed by freeze-pump-thaw three times. The orange mixture was heated to 90 °C and stirred for overnight. After cooling to room temperature, 50 mL of EtOAc and 50 mL of water were added. The aqueous phase was extracted by EtOAc (3 × 50 mL). The combined organic phase was washed with brine and water, dried over anhydrous Magnesium sulfate. After the solvent was removed by rotary evaporation, the residue was purified by silica gel chromatography using Hexanes/DCM (10/1) as eluent, affording the compound **1** as a white solid (91 mg, 60%). ^{1}H NMR (400 MHz, $CDCl_3$) δ 7.41- 7.35 (m, 6H, Ar-*H*), 6.94 (s, 8H, Ar-*H*), 6.78 (d, J = 8Hz, 4H, Ar-*H*), 6.65 (d, J = 8Hz, 4H, Ar-*H*), 2.52 (m, 8H, C*H*$_2$), 1.52 (m, 8H, C*H*$_2$), 1.33-1.11 (m, 8H, C*H*$_2$), 0.90 (t, 6H, C*H*$_3$), 0.88 (t, 6H, C*H*$_3$) ppm. ^{13}C NMR (101 MHz, $CDCl_3$) δ 141.9, 141.7, 140.5, 140.1, 139.7, 139.3, 139.2, 136.7, 131.5, 129.7, 129.4, 129.2, 129.1, 127.5, 127.1, 127.0, 35.2, 35.2, 33.5, 22.3, 21.9, 14.0 ppm.

m.p. 163.3-164.8 °C. HRMS (CI) for $C_{58}H_{63}$ [M]: 759.4930. Found: 759.4911. R_f: 0.25 (Hexane/DCM = 4/1)

FTIR (KBr, cm^{-1}): 4048, 3084, 3055, 3028, 2955, 2924, 2856, 1513, 1451, 1405, 852, 834, 793, 758.

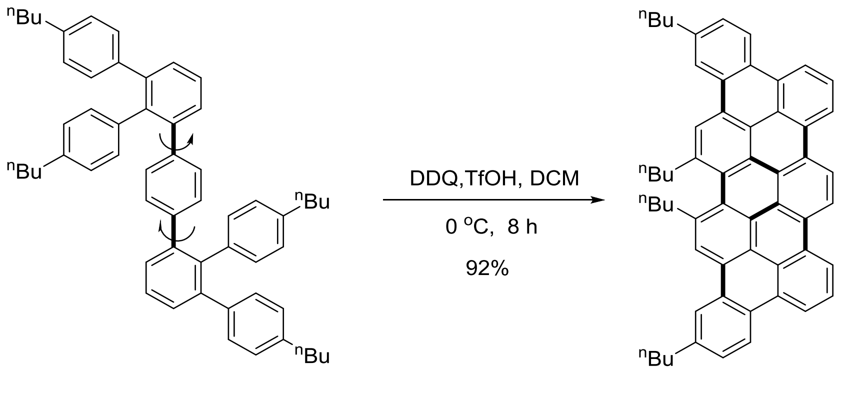

1 **G1**

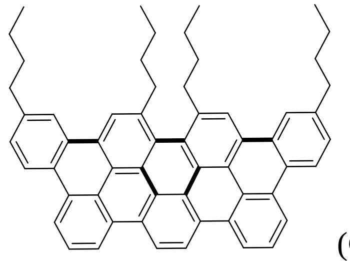

(**G1**). **1** (32 mg, 0.04 mmol) and DDQ (92 mg, 0.4 mmol) were dissolved in 10 mL of dry dichloromethane under argon atmosphere. The colorless solution was cooled down to 0 °C and 0.5 mL of CF_3SO_3H was added dropwise. The resulting black solution was stirred for 8 hours. After that, the reaction was quenched by saturated aqueous $NaHCO_3$ solution. The aqueous phase was extracted by dichloromethane (3 × 50 mL). The organic phase was collected and washed with water and brine and dried by anhydrous Na_2SO_4. After removal of solvent by rotary evaporation and drying under vacuum, **G1** was obtained as a yellow solid in 92 % yield (27.5 mg). ^{1}H NMR (500 MHz, $CDCl_3$) δ 9.31 (s, 2H), 9.12 (d, J = 10 Hz, 2H), 9.08 (s, 2H), 8.99 (d, J = 10 Hz, 2H), 8.85 (d, J = 10Hz, 2H), 8.81 (s, 2H), 8.13 (t, 2H), 7.68 (d, J = 5Hz, 2H), 3.82-3.70 (m, 4H), 3.06 (m, 4H), 1.97-1.91 (m, 4H), 1.63-1.58 (m, 4H), 1.48 (m, 2H), 1.16 (m, 2H), 1.08 (t, 6H), 0.84 (m, 4H), 0.50 (t, 6H). ^{13}C NMR (101 MHz, $CDCl_3$) δ 142.4, 139.3, 130.2, 129.7, 129.7, 128.3, 128.2, 127.9, 127.7, 126.4, 126.0, 125.5, 124.4, 124.1, 123.8, 123.4, 121.8, 121.6, 121.3, 121.1, 121.0, 36.2, 35.7, 35.5, 34.0, 22.7, 22.1, 14.1, 13.6.

m.p. > 300 °C. HRMS (CI) for $C_{58}H_{52}$ [M]: 748.4069. Found: 748.4088.

FTIR (KBr, cm^{-1}): 3078, 3028, 2955, 2927, 2857, 1616, 1593, 1464, 1481, 1393, 1376, 1259, 804, 775, 727.

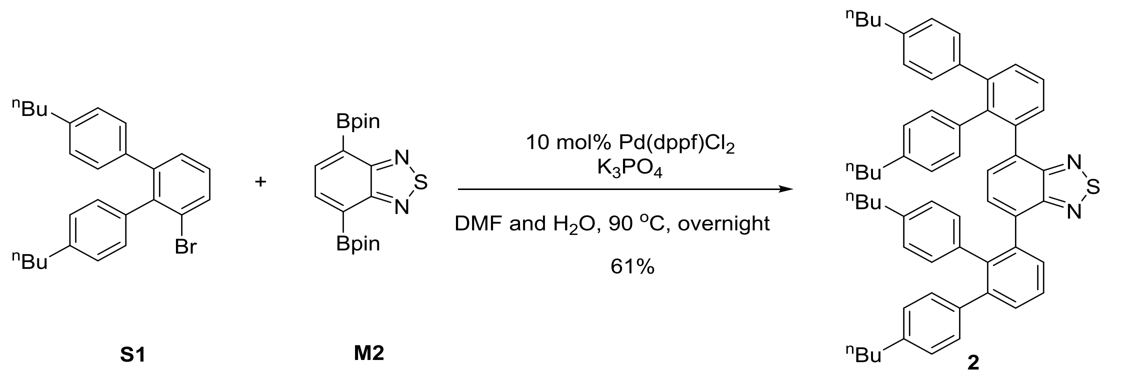

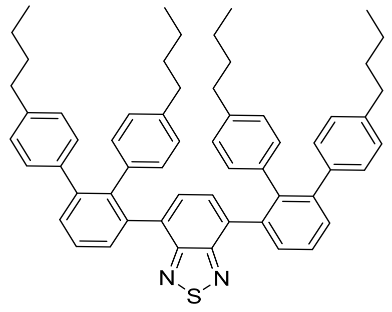

(**2**). **M2** (86.3 mg, 0.2 mmol), **S1** (210 mg, 0.5 mmol,), $K_3PO_4 \cdot H_2O$ (230 mg, 1 mmol, 5 equiv.) and [1,1′-bis(diphenylphosphino) ferrocene] dichloropalladium(II) complex with dichloromethane (16 mg) were mixed in a solution of DMF (3 mL) and water (0.6 mL). The mixture was degassed by freeze-pump-thaw three times. The orange mixture was heated to 90 ^{o}C and stirred for overnight (14 hours). After cooling to room temperature, 50 mL of EtOAc and 50 mL of water was added. The aqueous phase was extracted by EtOAc (3 × 50 mL). The combined organic phase was washed with brine and water, dried over anhydrous magnesium sulfate. After the solvents were removed by rotary evaporation, the residue was purified by silica gel chromatography using (Et_2O: Hexanes = 1:8) as eluent, affording the title compound **2** as a yellow gel in 61% yield (100 mg). ^{1}H NMR (400 MHz, $CDCl_3$) δ 7.51 (m, 6H, Ar-*H*), 6.98 (m, 10H, Ar-*H*), 6.65 (s, 8H, Ar-*H*), 2.54 (m, 4H, C*H₂*), 2.41 (m, 4H, C*H₂*), 1.58-1.13 (m, 16H, C*H₂*), 0.91(t, 6H, C*H₃*), 0.84(t, 6H, C*H₃*) ppm. ^{13}C NMR (101 MHz, $CDCl_3$) δ 153.9, 142.1, 140.6, 140.1, 140.1, 139.1, 137.4, 136.7, 133.6, 130.9, 130.5, 130.0, 129.8, 129.7, 127.5, 126.9, 126.9, 35.2, 35.1, 33.5, 33.40, 22.3, 21.8, 13.9, 13.9 ppm. HRMS (CI) for $C_{58}H_{61}N_2S$ [M]: 817.4555. Found: 817.4541. Rf: 0.5 (Et_2O: Hexane = 1:4)
FTIR (KBr, cm^{-1}): 3054, 3024, 2994, 2955, 2926, 2855, 1512, 1453, 1435, 1405, 1113, 891, 835, 796, 761.

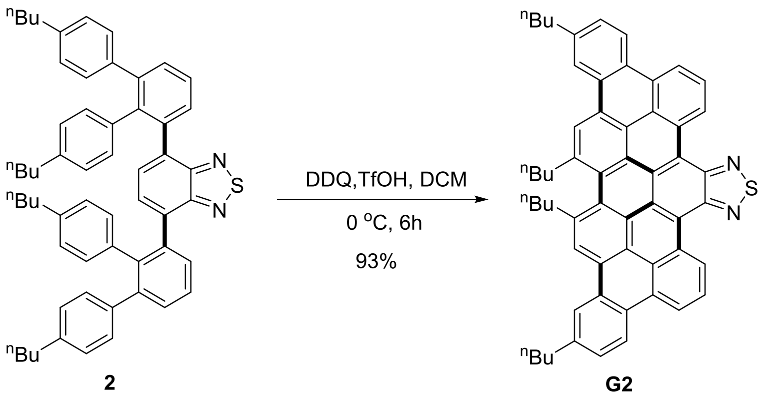

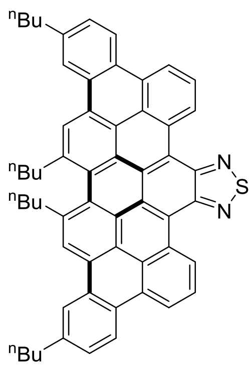

(**G2**). **2** (20 mg, 0.024 mmol) and DDQ (110 mg, 0.5 mmol) were dissolved in 10 mL of dry dichloromethane under nitrogen. The colorless solution was cooled down to 0 °C and 0.5 mL of CF_3SO_3H was added dropwise. The resulting black solution was stirred for 6 hours. After that, the reaction was quenched by saturated aqueous $NaHCO_3$ solution. The solution was extracted by dichloromethane (3 × 50 mL). The organic phase was collected and washed with water and brine, and dried by anhydrous Na_2SO_4. After removal of solvent by rotary evaporation and drying under vacuum, red solid was obtained in 93% yield (18 mg). ^{1}H NMR (400 MHz, $CDCl_3$) δ 10.50 (m, 1H), 9.11 (s, 1H), 8.77 (m, 2H), 8.68 (d, 1H), 7.96 (t, 1H), 7.62 (m, 1H), 3.65 (m, 2H), 3.05 (t, 2H), 1.94(m, 2H), 1.62 (m, 3H), 1.26 (m, 2H), 1.10 (t, 3H), 0.98-0.86 (m, 3H), 0.58 (t, 3H). ^{13}C NMR (101 MHz, $CDCl_3$) δ 152.3, 142.3, 139.2, 129.6, 128.7, 128.4, 128.2, 127.8, 127.7, 127.3, 127.1, 126.6, 125.7, 124.1, 123.7, 123.1, 122.9, 121.6, 121.1, 119.9, 36.2, 35.4, 35.1, 34.1, 22.7, 22.3, 14.2, 13.7.

mp > 300 °C. HRMS (CI) for $C_{58}H_{50}N_2S$ [M]: 806.3695. Found: 806.3683.

FTIR (KBr, cm^{-1}): 3075, 3024, 2955, 2926, 2856, 1616, 1594, 1389, 866, 816, 782, 729.

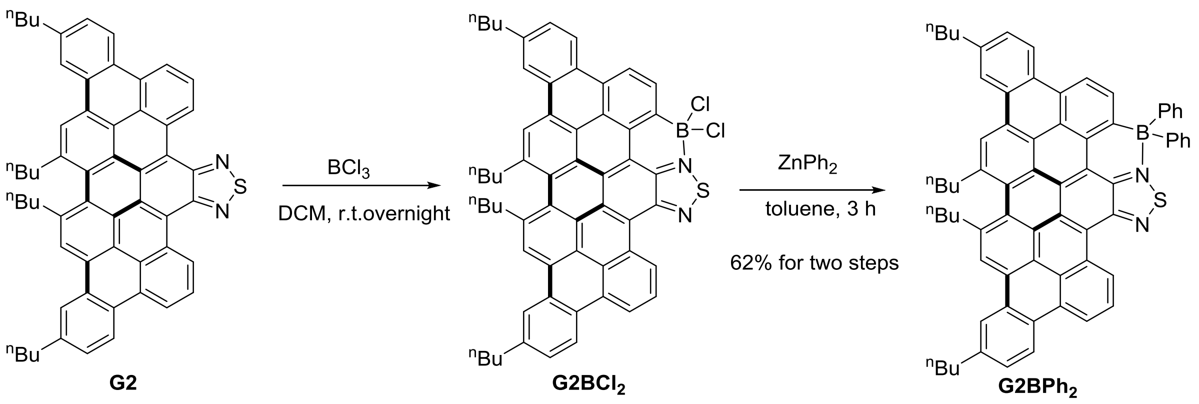

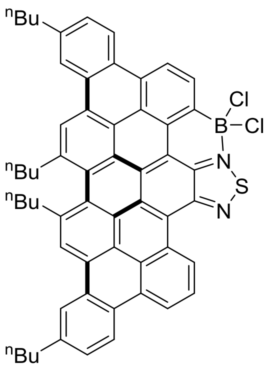

(**G2BCl$_2$**) A BCl_3 solution in pentane (0.12 mL, 0.12 mmol, 1M) was added to a red solution of **G2** (11 mg, 0.016 mmol) in DCM (4 mL) in a Schlenk tube at a -78 ^{0}C. The color of the solution changed to dark red. The reaction mixture was stirred at room temperature overnight under a dynamic flow of nitrogen, and then the solvent and excess BCl_3 was removed under reduced pressure to yield a dark solid. MALDI-tof mass for $C_{58}H_{49}BCl_2N_2S$ $[M]^+$: 886.31. Found: 885.89; for $C_{58}H_{49}BClN_2S$ $[M-Cl]^+$: 851.34. Found: 851.70.

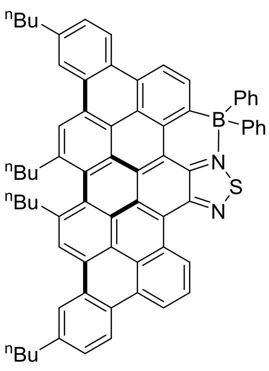

(**G2BPh$_2$**) $ZnPh_2$ (20 mg, 0.09 mmol) and 3 mL dry toluene were added into the Schlenk tube. The color of the solution changed to dark green. The resulting solution was stirred at room temperature for 3 hours, and then the solution was filtered through silica gel using DCM and hexane (2/3) as eluent. The solvents were removed under reduced pressure to

afford **G2BPh$_2$** as a dark solid (9.6 mg, 62% yield for two steps). ^{1}H NMR (500 MHz, CD_2Cl_2) δ 10.36 (d, J = 5Hz, 1H), 9.12 (d, J = 10Hz, 2H), 9.04 (d, J = 10Hz, 1H), 8.88 (d, J = 10Hz, 1H), 8.77 (s, 1H), 8.73 (s, 1H), 8.69 (d, J = 10Hz, 1H), 8.52 (d, J = 5Hz, 1H), 8.35 (d, J = 10Hz, 1H), 7.95 (t, 1H), 7.73 (d, J = 5Hz, 1H), 7.69 (d, J = 10Hz, 2H), 7.52 (d, J = 5Hz, 1H), 7.45 (t, 2H), 7.38 (t, 1H), 7.24 (d, J = 10Hz, 2H), 7.13 (t, 2H), 7.05 (t, 1H), 3.65 (m, 4H), 3.07 (t, 2H), 2.99 (t, 2H), 1.91 (m, 4H), 1.60 (m, 4H), 1.3 (m, 4H), 1.11 (t, 9H), 0.89 (m, 4H), 0.67 (t, 3H), 0.52 (t, 3H). ^{13}C NMR (126 MHz, CD_2Cl_2) δ 151.42, 147.03, 142.83, 142.28, 140.33, 139.78, 134.67, 133.12, 132.46, 129.51, 129.37, 129.17, 129.09, 128.81, 128.66, 128.61, 128.19, 128.06, 128.03, 127.88, 127.79, 127.74, 127.41, 127.07, 127.02, 126.85, 126.36, 126.31, 126.26, 125.54, 124.94, 124.32, 124.28, 124.23, 124.01, 123.53, 123.38, 123.21, 123.04, 122.19, 122.06, 121.57, 118.62, 36.13, 36.04, 35.40, 35.20, 35.09, 34.81, 33.99, 33.88, 29.68, 22.68, 22.31, 21.96, 13.86, 13.50, 13.34, 0.75.

MALDI-tof mass for $C_{70}H_{59}BN_2S$ [M]: 970.45. Found: 969.93. and for $C_{64}H_{54}BN_2S$ $[M-Ph]^+$: 893.41. Found: 893.81. R_f: 0.7 (hexane : DCM = 2 : 3)

FTIR (cm^{-1}): 3067, 3046, 3001, 2956, 2926, 2856, 1615, 1594, 1568, 1530, 1486, 1455, 1431, 1397, 1379, 1261, 1101, 1031, 869, 816, 797, 737, 705.

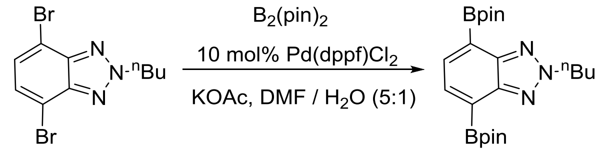

S5 M3

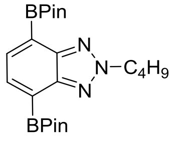

(**M3**) **S5** (1.32 g, 4 mmol), 4,4,4',4',5,5,5',5'-octamethyl-2,2'-Bi-(1,3,2-dioxaborolane (2.34 g, 9.2 mmol), potassium acetate (2.35 g, 24 mmol) and [1,1′-bis(diphenylphosphino)ferrocene]dichloropalladium(II) complex with dichloromethane (327 mg) were mixed in 20 mL of 1,4-dioxane. The mixture was degassed by freeze-pump-thaw three times and then heated to 80 °C overnight. After cooling to room temperature, 50 mL of water and 50 mL of ethyl acetate was added. The aqueous phase was extracted by ether (3 × 50 mL). The combined organic phase washed with brine and water, dried over anhydrous magnesium sulfate.

After the solvents were removed by rotary evaporation, the residue was purified by silica gel chromatography using hexanes and ethyl acetate (4/1) as eluent, affording **M3** as a white solid in 78 % yield (1.33 g). δ ^{1}H NMR (400 MHz, $CDCl_3$) δ 7.86 (s, 2H), 4.82 (m, 2H), 2.12 (m, 2H), 1.43(m, 2H), 1.42 (s, 24H), 0.99 (m, 3H). ^{13}C NMR (101 MHz, $CDCl_3$) δ 146.3, 134.1, 84.1, 56.4, 32.0, 24.9, 20.0, 13.7.

MP: 153 - 154 °C. HR-MS (ESI) for $C_{22}H_{35}B_2N_3O_4$ [M]: 425.2886. Found: 425.2929. R_f: 0.5 (hexane : Ether = 4 : 1)

FTIR (cm^{-1}): 3052, 3030, 2979, 2934, 2874, 1521, 1457, 1388, 1372, 1340, 1285, 1186, 1166, 1140, 1009, 992, 958, 858, 802, 711, 700, 668.

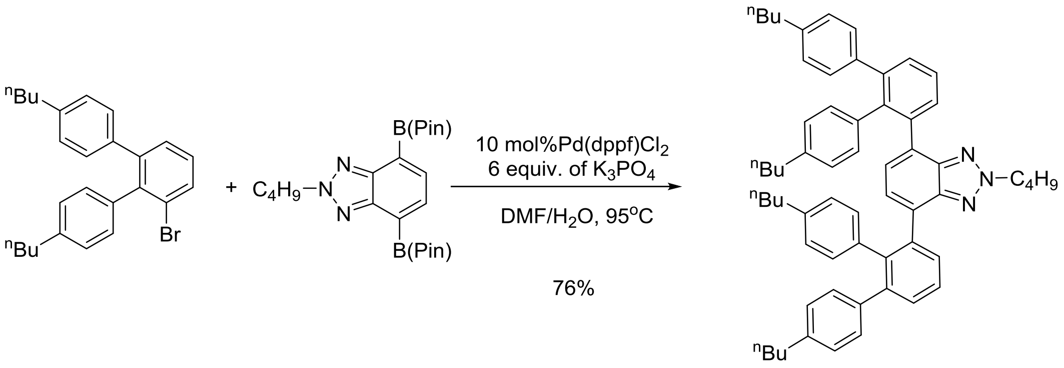

S1 **M3** **3**

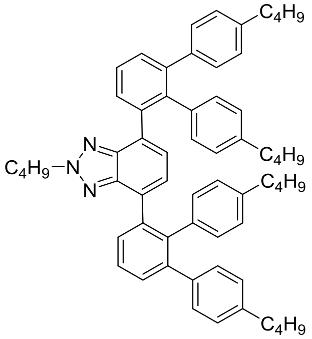

(**3**) **M3** (85.4 mg, 0.2 mmol), and **S1** (210 mg, 0.5 mmol), $K_3PO_4{\cdot}H_2O$ (278 mg, 1.2 mmol) and [1,1′-bis(diphenylphosphino) ferrocene]dichloropalladium(II) complex with dichloromethane (21 mg) were mixed in a solution of DMF (3 mL) and water (0.6 mL). The mixture was degassed by freeze-pump-thaw three times. The orange mixture was heated to 95 °C and stirred for overnight (14 hours). After cooling to room temperature, 50 mL of EtOAc and 50 mL of water was added. The aqueous phase was extracted by ethyl acetate (3 × 50 mL). The combined organic phase was washed with brine and water, dried over anhydrous magnesium sulfate. After the solvents were removed by rotary evaporation, the residue was purified by silica

gel chromatography using ether/Hexanes (1/4) as eluent, affording **3** as a yellow gel in 76% yield (0.130 g). ^{1}H NMR (500 MHz, $CDCl_3$) δ 7.56 (m, 2H), 7.49 (d, 4H), 6.97 (m, 8H), 6.74 (s, 2H), 6.68 (m, 8H), 4.53 (t, 2H, C*H$_2$*), 2.56 (t, 4H, CH_2), 2.43 (t, 4H, CH_2), 1.90 (m, 2H), 1.56 (m, 4H, CH_2), 1.46 (m, 4H, CH_2), 1.32 (m, 6H, CH_2), 1.22 (m, 4H, CH_2), 0.90 (m, 15H, CH_3). ^{13}C NMR (126 MHz, $CDCl_3$) δ 143.5, 142.1, 140.5, 140.0, 139.9, 139.4, 137.8, 137.0, 131.1, 130.5, 130.1, 129.8, 127.5, 127.1, 126.9, 126.8, 56.2, 35.2, 35.1, 33.5, 33.4, 32.0, 22.3, 21.9, 21.8, 19.8, 13.9, 13.6.

HR-MS (ESI) for $C_{62}H_{69}N_3$[M]: 855.5491. Found: 855.5497. R_f: 0.2 (hexane : DCM = 2 : 1).
FTIR (cm^{-1}): 4334, 4057, 3127, 3050, 3022, 2956, 2929, 2871, 2858, 1579, 1514, 1456, 1406, 1378, 1186, 1115, 1021, 1005, 837, 805.

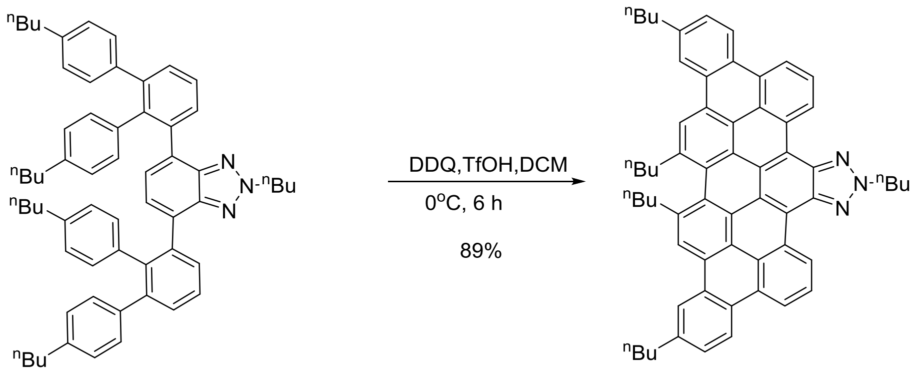

3 **G3**

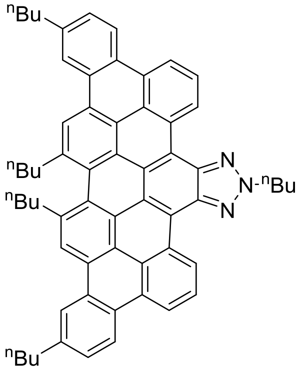

G3

3 (20 mg, 0.024mmol) and DDQ (74 mg, 0.32 mmol) were dissolved in 10 mL of dry dichloromethane under nitrogen atmosphere. The yellow solution was cooled down to 0 °C and 0.5 mL of CF_3SO_3H was then added dropwise. After that, the resulting black solution was stirred for 6 hours. The reaction was quenched by saturated aqueous $NaHCO_3$ solution. The

solution was extracted by dichloromethane (3 × 20 mL). The organic phase was collected and washed with water and brine, dried by anhydrous $MgSO_4$. After removal of solvent by rotary evaporation and drying under vacuum, yellow solid was obtained in 89% yield (18 mg). ^{1}H NMR (500 MHz, $CDCl_3$) δ 10.62 (s, 2H), 9.11(s, 2H), 9.05(s, 2H), 8.87 (m, 4H), 8.28 (t, 2H), 7.68 (m, 2H), 5.14 (m, 2H), 3.72 (m, 4H), 3.06 (t, 4H), 2.42 (m, 2H), 1.96 (m, 4H), 1.62 (m, 4H), 1.28 (m, 4H), 1.13(m, 11H), 0.90 (m, 4H), 0.52 (t, 6H). ^{13}C NMR (126 MHz, $CDCl_3$) δ 142.2, 141.8, 139.2, 130.1, 129.1, 128.7, 128.4, 128.3, 127.8, 127.4, 126.8, 126.6, 126.3, 124.8, 124.1, 123.7, 123.4, 122.0, 121.4, 121.1, 119.5, 56.8, 36.2, 35.5, 35.3, 34.0, 32.4, 31.6, 29.7, 22.7, 22.7, 22.1, 20.09, 14.1, 13.6.

HR-MS (ESI) for $C_{62}H_{59}N_3$[M]: 845.4709. Found: 845.4685. R_f: 0.5 (Hexane : DCM = 2:1)

FTIR (cm^{-1}): 3111, 3076, 3029, 2956, 2927, 2857, 1617, 1593, 1458, 1389, 1378, 1102, 868, 811, 783, 732.

Synthesis of polymer precursors

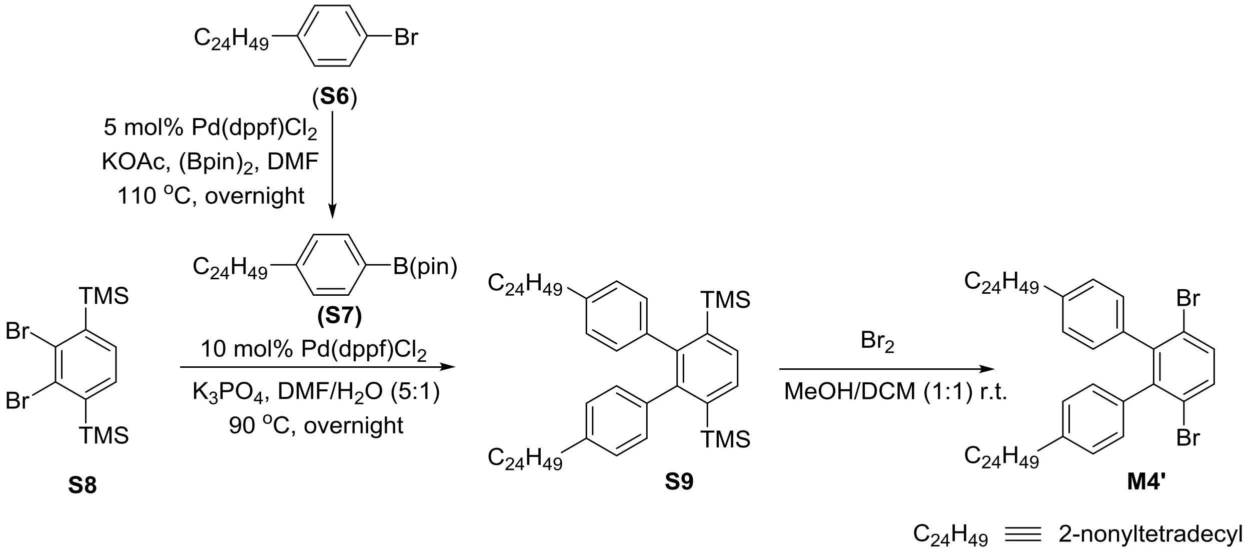

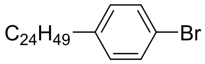 **(S6)**. To a suspension of Mg (2.16 g, 90 mmol) and a catalytic amount of I_2(10 mg) in 15 mL of dry THF in a 100 mL of two-necked RBF equipped with a condenser was added 11-(bromomethyl)tricosane[6] (12.51 g, 30 mmol) under nitrogen atmosphere. After the mixture was stirred at room temperature for a few minutes, the mixture was refluxed for 2 hours. To a solution of $ZnBr_2$ (6.73 g, 30 mmol) in 40 mL of dry THF in a two-necked flask in an ice/water bath, the Grignard reagent was added dropwise under nitrogen atmosphere. After stirring for 30 minutes, 1-bromo-4-iodobenzene (7.08 g, 25 mmol) and Pd(dppf)Cl_2 (491 mg, 0.6 mmol) were added to the mixture and stirred for 10 minutes. After that, the yellow mixture was heated up to 75 °C for overnight. Upon cooling to room temperature, 50 mL of diethyl ether and 50 mL of 1 M HCl (aq) was added to the reaction mixture. The aqueous phase was extracted by ether (3 × 50 mL). The combined organic phase was washed with brine and water, dried over anhydrous magnesium sulfate. After the solvents were removed by rotary evaporation, the residue was purified by silica gel chromatography with hexane as eluent. The compound **S6** was obtained as a colorless oil (7.34 g, 60 mmol). ^{1}H NMR (400 MHz, $CDCl_3$) δ 7.40 (d, J = 8Hz, 2H, Ar-*H*), 7.03 (d, J = 8Hz, 2H, Ar-*H*), 2.50 (d, J = 4 Hz, 2H, CH_2), 1.60 (m, 1H, C*H*), 1.29 (m, 40H, CH_2), 0.89 (t, 6H, CH_3). ^{13}C NMR (126 MHz, $CDCl_3$) δ 140.8, 131.1, 130.9, 119.2, 39.9, 39.6, 33.1, 31.9, 31.9, 29.9, 29.7, 29.7, 29.7, 29.6, 29.4, 29.4, 26.5, 22.7, 14.1.

HR-MS (ESI) for $C_{30}H_{53}Br$ [M]: 492.3331. Found: 492.3193. R_f: 0.9 (hexane)

FTIR (KBr, cm^{-1}): 3080, 3027, 2956, 2926, 2852, 1489, 1468, 1403, 1373, 1074, 1012, 829, 793, 719.

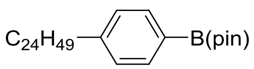

(**S7**). 1-Bromo-4-(2-decyltetradecyl)benzene (8.3 g, 16.8 mmol), 4,4,4',4',5,5,5',5'-octamethyl-2,2'-bi-(1,3,2-dioxaborolane (4.28 g, 16.9 mmol), potassium acetate (4.94 g, 50.4 mmol) and [1,1′-bis(diphenylphospino)ferrocene]dichloropalladium(II) complex with dichloromethane (500 mg, 0.61 mmol) were mixed in 30 mL of DMF. The mixture was degassed via freeze-pump-thaw for three times and then heated to 110 °C for overnight. After cooling to room temperature, 100 mL of water and 100 mL of ethyl acetate was added. The aqueous phase was extracted by ether (3 × 100 mL). The combined organic phase was washed with brine and water, dried over anhydrous magnesium sulfate. After the solvents were removed by rotary evaporation, the residue was purified by silica gel chromatography using DCM/hexanes (1/4) as eluent. **S7** was obtained as a light-yellow oil (5.55 g, 61%). ^{1}H NMR (400 MHz, $CDCl_3$) δ 7.72 (d, J = 8 Hz, 2H, Ar-*H*), 7.16 (d, J = 8 Hz, 2H, Ar-*H*), 2.53 (d, J = 8 Hz, 2H, CH_2), 1.60 (m, 1H, C*H*), 1.35 (s, 12H, CH_3), 1.33-1.21 (m, 40H, CH_2), 0.88 (t, 6H, CH_3). ^{13}C NMR (101 MHz, $CDCl_3$) δ 145.4, 134.6, 128.7, 83.6, 40.8, 39.6, 33.1, 31.9, 30.0, 29.7, 29.7, 29.7, 29.4, 26.5, 24.9, 22.7, 14.1.
HR-MS (ESI) for $C_{36}H_{65}BO_2$[M]: 539.5114. Found:539.4947. R_f: 0.6 (DCM: hexane = 1:4)
FTIR (KBr, cm^{-1}): 3080, 3047, 2956, 2924, 2853, 1612, 1517, 1466, 1399, 1361, 1319, 1272, 1215, 1146, 1090, 1022, 963, 861, 659.

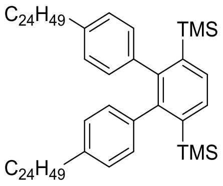

(**S9**). **S8** (247 mg, 0.65 mmol), **S7** (1.06 g, 1.95 mmol), K_3PO_4 (0.827 g, 3.9 mmol) and [1,1′-bis(diphenylphosphino)ferrocene]dichloropalladium(II) complex with DCM (57 mg, 0.07 mmol) was mixed in a solution of DMF (5 ml) and water (1 mL). The mixture was degassed via freeze-pump-thaw for three times, and then heated to 90 °C and stirred for overnight. The black mixture was then cooled to room temperature. 50 mL of EtOAc and 50 mL of water were added. The aqueous phase was extracted by ethyl acetate (3 × 50 mL). The combined organic phase was washed with brine and water, dried over magnesium sulfate. After

the solvents were removed by rotary evaporation, the residue was purified by silica gel chromatography using hexane as eluent, affording **S9** as a colorless oil (0.48 g, 71%). ^{1}H NMR (500 MHz, $CDCl_3$) δ 7.62 (s, 2H, Ar-*H*), 6.82(s, 8H, Ar-*H*), 2.38 (d, 4H, CH_2), 1.86 (m, 2H, CH), 1.26 (m, 80H, CH_2), 0.88 (t, 12H, CH_3), -0.06 (s, 18H, Si-CH_3). ^{13}C NMR (126 MHz, $CDCl_3$) δ 147.5, 140.1, 139.6, 139.4, 132.5, 130.7, 127.5, 40.1, 39.7, 32.9, 31.9, 30.1, 29.7, 29.7, 29.4, 26.6, 22.7, 14.1, 0.5.

HR-MS(ESI) for $C_{72}H_{126}Si_2$[M]: 1046.9398. Found:1046.9119. R_f: 0.8 (hexane)

FTIR (KBr, cm^{-1}): 3053, 3024, 2956, 2924, 2853, 1512, 1466, 1406, 1377, 1247, 1187, 1044, 1018, 838, 757, 721, 688.

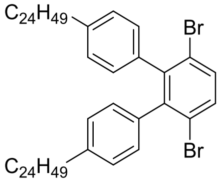

(**M4'**). To a solution of **S7** (0.95 g, 0.91 mmol) in DCM (5 mL) and methanol (5 mL), bromine (0.434 g, 2.73 mmol) was added dropwise. The mixture was stirred at room temperature for 19 hours. The reaction was quenched by the saturated aqueous sodium sulfite solution and DCM (100 mL) was added. The aqueous phase was extracted by DCM (3 × 50 mL). The combined organic phase was washed with water and brine, dried over magnesium sulfate. After the solvents were removed by rotary evaporation, the residue was purified by silica gel chromatography using hexane as eluent, affording **M4'** as a colorless oil (0.82 g, 85%). ^{1}H NMR (400 MHz, $CDCl_3$) δ 7.52 (s, 2H, Ar-*H*), 6.90-6.82 (m, 8H, Ar-*H*), 2.40 (d, J = 8 Hz, 4H, CH_2), 1.54 (m, 2H, C*H*), 1.30-1.20 (m, 80H, CH_2), 0.87 (t, 12H, CH_3). ^{13}C NMR (101 MHz, $CDCl_3$) δ 144.1, 140.5, 137.3, 132.5, 129. 6, 128.1, 123.3, 40.2, 39.4, 33.0, 31.9, 31.6, 30.1, 29.8, 29.7, 29.7, 29.7, 29.7, 29.4, 29.4, 29.4, 26.5, 22.7, 22.7, 14.1.

HR-MS(CI) for $C_{66}H_{108}Br_2$[M]: 1058.6818. Found:1058.6641. R_f: 0.7 (hexane)

FTIR (KBr, cm-1):3086, 3053, 3027, 2956, 2924, 2853, 1515, 1465, 1406, 1146, 1029, 1007, 844, 806, 721.

Polymerization

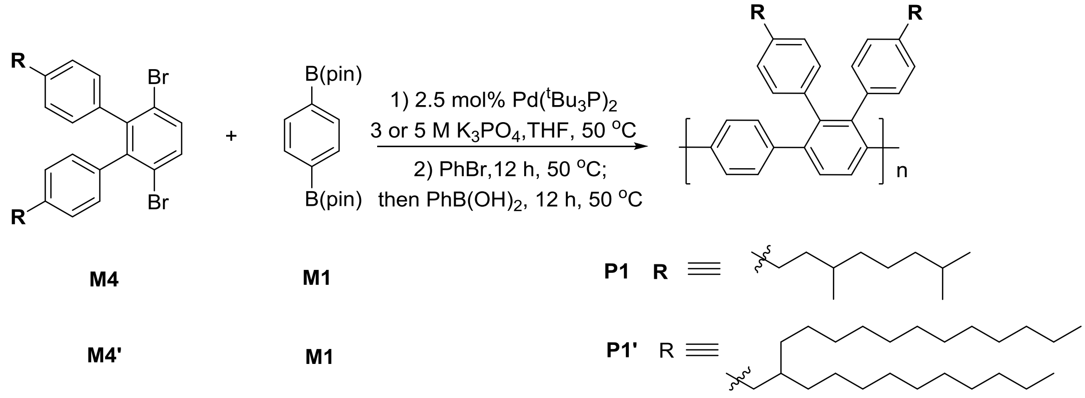

P1: M4 (133 mg, 0.2 mmol) and **M1** (66 mg, 0.2 mmol), Pd(^{t}Bu$_3$P)$_2$ (2.5 mg) and 0.5 mL of (aqueous, 5 M) K_3PO_4 and 0.5 mL of degassed THF were mixed in a vial (8 mL) in a glovebox. The vial was sealed and the solution was stirred at 50 °C for 12 hours. Bromobenzene (1 equiv.) was then added, and the mixture was stirred for 12 hours. Then, phenyborinic acid (1 equiv.) was added, and the resulting mixture was stirred for another 12 hours. Upon cooling to room temperature, water (30 mL) and DCM (30 mL) were added. The aqueous phase was extracted by DCM (3 × 10 mL). The combined organic phase was washed with water. The solution was concentrated by rotary evaporation to 1 - 2 mL, and the resulting solution was added into 30 mL of acidic methanol. After stirred at room temperature for 2 - 3 hours, the polymer was filtered and dried at room temperature overnight to give a white solid (112 mg, 96% yield). After that, the solid was fractionated by soxhlet extraction with boiling acetone for 2 days, and then dried in vacuum at room temperature, affording white solid **P1 (**79.4 mg) in 68% yield. GPC data: Mn: 19.6 kDa; Mw: 28.1 kDa; **Đ:** 1.43. ^{1}H NMR (400 MHz, $CDCl_3$) δ 7.40 (s, 2H, Ar-*H*), 6.84 (s, 4H, Ar-*H*), 6.70 (m, 4H, Ar-*H*), 6.58(m, 4H, Ar-*H*), 2.45 (m, 4H, CH_2), 1.51 (m, 4H, CH_2), 1.27-1.11 (m, 16H, CH_2), 0.85 (m, 18H, CH_3) ppm. ^{13}C NMR (101 MHz, $CDCl_3$) δ 140.6, 140.3, 140.0, 139.7, 137.1, 131.5, 129.0, 128.7, 126.9, 126.8, 39.3, 38.8, 37.1, 33.0, 32.2, 27.9, 24.7, 22.7, 22.6, 19.6 ppm.

FTIR (KBr, cm^{-1}): 4052, 3082, 3051, 3025, 2954, 2925, 2867, 1516, 1450, 1407, 1383, 1019, 1005, 819, 668.

P1': M4' (212 mg, 0.2 mmol) and **M1** (66 mg, 0.2 mmol), Pd(^{t}Bu$_3$P)$_2$ (5 mg), 0.5 mL of (aqueous, 3 M) K_3PO_4 and degassed THF (0.5 mL) were mixed in a vial (8 mL) in glovebox. The vial was sealed and the solution was stirred at 50 °C for 24 hours, followed by the same

workup and purification protocol as **P1**. **P1'** was isolated as colorless gel (144 mg) in 74% yield. GPC data: Mn: 6.5 kDa; Mw: 8.5 kDa; **Đ:** 1.32. ^{1}H NMR (500 MHz, $CDCl_3$) δ = 7.39 (m, 2H), 7.13 (m, 2H), 7.12-6.60 (m, 10H), 2.40 (m, 4H), 1.46 (m, 2H), 1.26 (m, 80H), 0.91 (m, 12H). ^{13}C NMR (126 MHz, $CDCl_3$) δ 140.6, 140.4, 140.3, 139.8, 138.8, 138.7, 137.3, 137.2, 131.5, 131.4, 129.9, 129.1, 127.6, 127.4, 40.3, 40.2, 40.2, 39.7, 39. 6, 33.2, 33.0, 33.0, 32.0, 30.2, 30.1, 30.0, 29.8, 29.7, 29.42, 26.6, 22.7, 14.1.

FTIR (KBr, cm^{-1}): 4334, 4258, 4059, 3086, 3054, 3029, 2928, 2855, 1515, 1467, 1379, 1249, 1027, 859, 821, 755, 719, 701.

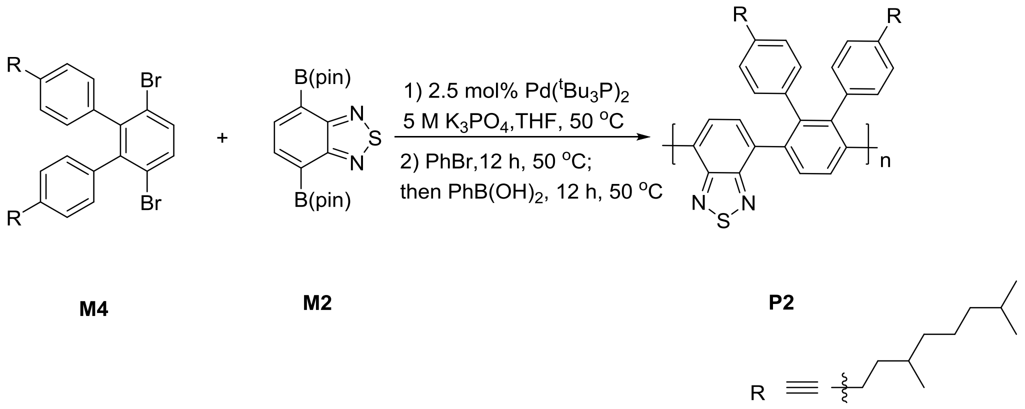

P2: **M4** (133 mg, 0.2 mmol) and **M2** (77.6 mg, 0.2 mmol), Pd(^{t}Bu$_3$P)$_2$ (2.5 mg) and 0.5 mL of (aqueous, 5 M) K_3PO_4 and degassed THF (0.5 mL) were mixed in a vial (8 mL) in glovebox. The vial was sealed and the solution was stirred at 50 °C for 12 hours. Bromobenzene (1 equiv.) was then added, and the mixture was stirred for 12 hours. Then, phenyborinic acid (1 equiv.) was added, and the resulting mixture was stirred for another 12 hours. After cooling to room temperature, 30 mL of water and 30 mL of DCM were added. The aqueous phase was extracted by DCM (3×10 mL). The combined organic phase washed with water. The solution was concentrated by rotary evaporation to 2 mL, and the resulting solution was added into 30 mL of acidic methanol. After stirred at room temperature for 2-3 hours, the polymer was filtered and dried at room temperature for overnight to yield **P2** as a yellow solid (121 mg, 94% yield). After that, the solid was fractionated by soxhlet extraction with boiling acetone for 2 days, and then dried in vacuum at room temperature. **P2** was obtained as a yellow solid (90 mg) in 70% yield. GPC data: Mn: 17.4 kDa; Mw: 25.4 kDa; **Đ:** 1.46. ^{1}H NMR (400 MHz, $CDCl_3$) δ 7.65 (s, 2H, Ar-*H*), 7.00 (s, 2H, Ar-*H*), 6.97 (s, 8H, Ar-*H*), 2.37 (m, 4H, C*H*$_2$), 1.54-1.05 (m, 16H, C*H*$_2$),

0.88-0.80 (m, 18H, CH_3). ^{13}C NMR (101 MHz, $CDCl_3$) δ 154.1, 141.5, 140.3, 136.8, 133.3, 130.9, 130.3, 129.5, 126.6, 39.3, 38.8, 37.1, 32.9, 32.3, 27.9, 24.7, 22.7, 22.6, 19.6.

FTIR (KBr, cm^{-1}): 4052, 3129, 3089, 3048, 3025, 2954, 2925, 2867, 1514, 1455, 1406, 1383, 1366, 1345, 1182, 1169, 1021, 958, 887, 824.

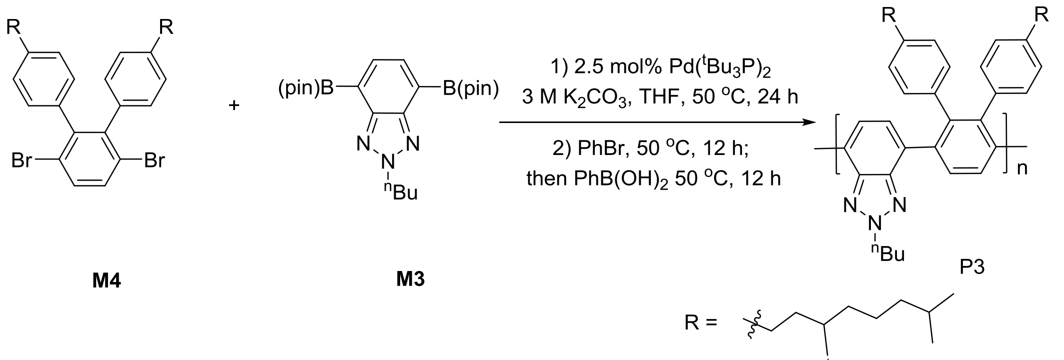

P3: **M3** (85.4 mg, 0.2 mmol) and M4 (133.2 mg, 0.2 mmol) and Pd(^{t}Bu$_3$P)$_2$ (2.5 mg) and 0.5 mL of (aqueous, 3 M) K_2CO_3 and degassed THF (0.5 mL) were mixed in a vial (8 mL) in glovebox. The vial was sealed and the solution was stirred at 50 ^{o}C for 8 hours. Bromobenzene (1 equiv.) was then added, and the mixture was stirred for 12 hours. Then, phenyborinic acid (1 equiv.) was added, and the resulting mixture was stirred for another 12 hours. After cooling to room temperature, 30 mL of water and 30 mL of DCM were added. The aqueous phase was extracted by DCM (3×10 mL). The combined organic phase washed with water. The solution was concentrated by rotary evaporation to 1 - 2 mL, and the resulting solution was added into 30 mL of acidic methanol. After stirred at room temperature for 2 - 3 hours, the polymer was filtered and dried at room temperature for overnight to yield **P3** as a yellow solid (112 mg) in 82% yield. After that, the solid was fractionated by soxhlet extraction with boiling acetone for 2 days, and then dried in vacuum at room temperature. **P3** was obtained as a yellow solid (90 mg) in 66% yield. GPC data: Mn: 21.6 kDa; Mw: 37.4 kDa; **Đ:** 1.73. ^{1}H NMR (500 MHz, $CDCl_3$) δ = 7.68 (s, 2H), 6.72 (s, 2H), 6.65 (m, 8H), 4.57 (s, 2H), 2.41(m, 4H), 1.96 (s, 2H), 1.55-1.46 (m, 5H), 1.34 - 1.22 (m, 12H), 1.17 - 1.09 (m, 5H), 0.98 (t, 3H, CH_3), 0.88(t, 18H, CH_3). ^{13}C NMR (126 MHz, $CDCl_3$) δ = 143.6, 141.2, 139.8, 137.3, 131.2, 130.5, 129.61, 127.3, 126.4, 56.2, 39.4, 38.8, 37.1, 33.1, 32.4, 32.1, 27.9, 24.7, 22.7, 22.6, 19.9, 19.6, 13.6.

FTIR (cm^{-1}): 4055, 3083, 3051, 3026, 2955, 2926, 2868, 1514, 1459, 1376, 1283, 1197, 1170, 1104, 1021, 973, 824.

Synthesis of Graphene Nanoribbons

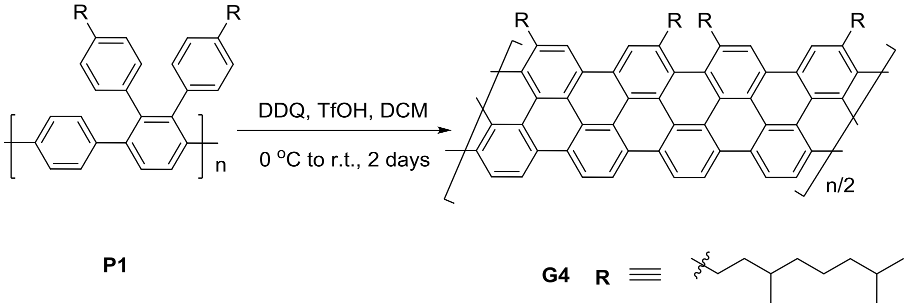

G4: **P1** (17.5 mg) and DDQ (66 mg) were dissolved in 10 mL of dichloromethane under nitrogen atmosphere. The yellow solution was stirred and cooled down in an ice/water bath for 10 min. Then, CF_3SO_3H (0.5 mL) was added, and the black mixture was warmed to room temperature and stirred for two days. The reaction was quenched by saturated aqueous $NaHCO_3$ solution. The black solid was filtered and washed with water and methanol. The solid was further washed by boiling methanol for 12 hours, and then boiling acetone for 12 hours and boiling hexane for 12 hours, and then dried under vacuum at room temperature. **G4** was obtained as a black solid in 86% yield (15 mg).

FTIR (KBr, cm^{-1}): 2950, 2920, 2863, 1633, 1569, 1427, 1209, 889, 804, 747, 622.

G4': **P1'** (14.7 mg) and DDQ (42 mg) were dissolved in 10 mL of dichloromethane under nitrogen atmosphere. The yellow solution was stirred and cooled down in an ice/water bath for 10 min. After that, CF_3SO_3H (0.5 mL) was added, and the black mixture was warmed to room temperature and stirred for two days. The reaction was quenched by saturated aqueous $NaHCO_3$ solution. The aqueous phase was extracted by DCM (3 × 50 mL). The combined organic phase was washed with water, dried over anhydrous magnesium sulfate. After the solvents were removed by rotary evaporation, **G4'** was obtained as a red solid in 88% yield (13 mg).

FTIR (KBr, cm^{-1}): 2957, 2923, 2849, 1643, 1569, 1454, 1262, 1215, 885, 798.

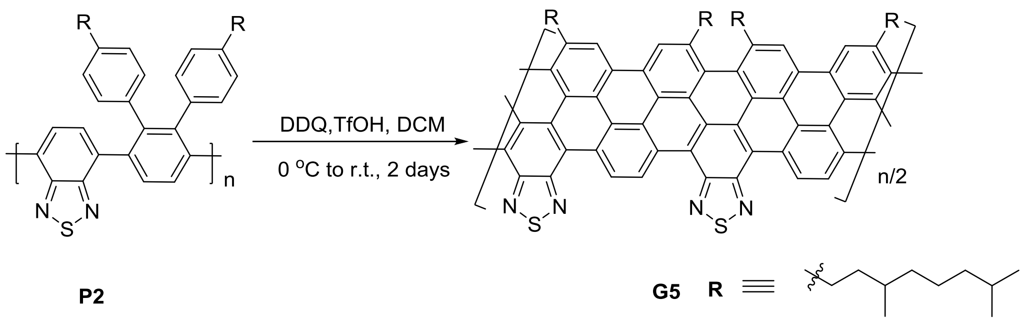

G5: **P2** (18.9 mg) and DDQ (65 mg) were dissolved in 10 mL of dichloromethane under nitrogen atmosphere. The yellow solution was stirred and cooled down in an ice/water bath for 10 min. Then, CF_3SO_3H (0.5 mL) was added, and the black mixture was warmed to room temperature and stirred for two days. The reaction was quenched by saturated aqueous $NaHCO_3$ solution. The black solid was filtered and washed with water and methanol. The solid was further washed with boiling methanol for 12 hours, and then boiling acetone for 12 hours and boiling hexane for 12 hours, and then dried under vacuum at room temperature. **G5** was obtained as a black solid in 95% yield (18 mg).

FTIR (KBr, cm^{-1}): 2950, 2919, 2863, 1636, 1434, 1370, 1215, 889, 835, 750, 619.

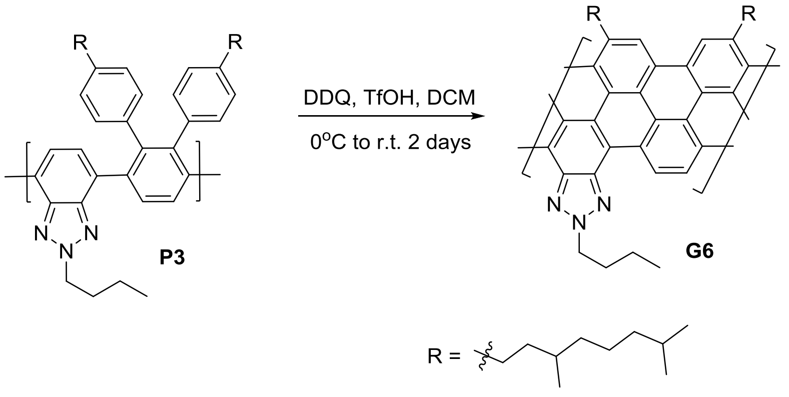

G6: **P3** (13.6 mg) and DDQ (44 mg) were dissolved in 10 mL of dichloromethane under nitrogen atmosphere. The yellow solution was stirred and cooled down in an ice/water bath for 10 min. After that, CF_3SO_3H (0.5 mL) was added, and the black mixture was warmed to room temperature and stirred for two days. Then, the reaction was quenched by saturated aqueous $NaHCO_3$ solution. The black solid was filtered and washed with water and methanol. The solid was further washed with boiling methanol for 12 hours, and then boiling acetone for 12 hours

and boiling hexane for 12 hours, and then dried under vacuum at room temperature. **G6** was obtained as a black solid in 80% yield (11 mg).

FTIR (KBr, cm^{-1}): 2955, 2925, 2867, 1652, 1635, 1578, 1219, 890, 840, 734.

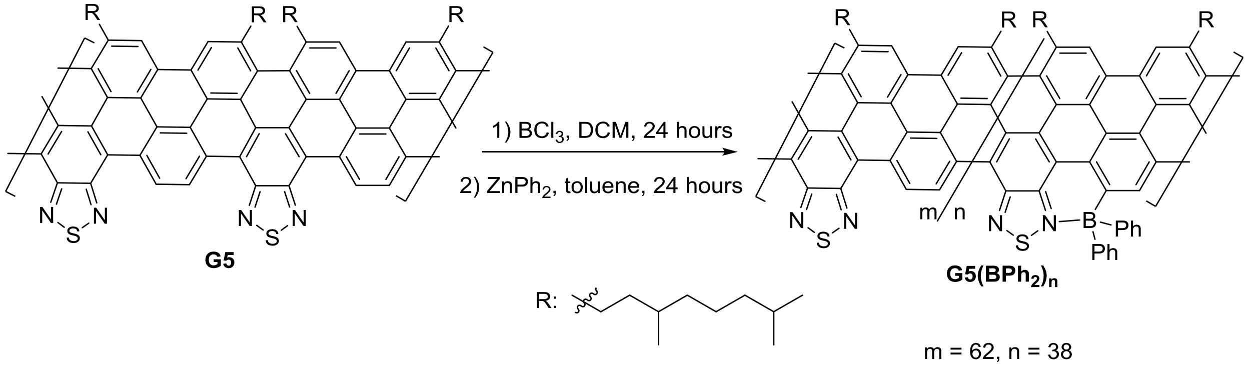

$G5(BPh_2)_n$ A BCl_3 solution in pentane (0.3 mL, 0.3 mmol, 1M) was added dropwise to a suspension of **G5** (5 mg) in DCM (8 mL) in a Schlenk tube at a -78 °C. The reaction mixture was stirred at room temperature for 24 hours under a dynamic flow of nitrogen, and then the solvent and excess BCl_3 was removed under reduced pressure to yield a black solid. The $ZnPh_2$ (20 mg, 0.09 mmol) and 3 mL dry toluene were added. The resulting solution was stirred at room temperature for 24 hours. The solvents were removed under reduced pressure, and the solid was further washed with boiling methanol for 12 hours, and then boiling acetone for 4 hours and boiling hexane for 4 hours, and then dried under vacuum at room temperature. **$G5(BPh_2)_n$** was afforded as a dark powder (4.5 mg).

FTIR (cm^{-1}): 2954, 2921, 2861, 1622, 1583, 1256, 1411, 1093, 1054, 899, 837, 507.

X-ray data

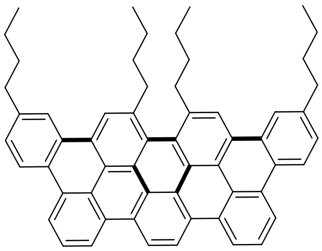

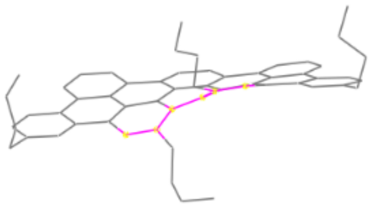

Crystal data and structure refinement for **G1**.

Identification code	**G1**	
Empirical formula	C58 H52	
Formula weight	748.99	
Temperature	293(2) K	
Wavelength	1.54184 Å	
Crystal system	Triclinic	
Space group	P -1	
Unit cell dimensions	a = 8.9025(2) Å	α = 64.971(2)°.
	b = 14.8438(3) Å	β = 80.154(2)°.
	c = 17.2013(5) Å	γ = 75.196(2)°.
Volume	1986.13(9) $Å^3$	
Z	2	
Density (calculated)	1.252 Mg/m^3	
Absorption coefficient	0.528 mm^{-1}	
F(000)	800	
Crystal size	0.500 x 0.200 x 0.100 mm^3	
Theta range for data collection	2.842 to 73.538°.	
Index ranges	-11<=h<=11, -18<=k<=18, -20<=l<=21	
Reflections collected	36741	
Independent reflections	7845 [R(int) = 0.0272]	
Completeness to theta = 67.684°	99.6 %	
Absorption correction	Semi-empirical from equivalents	
Refinement method	Full-matrix least-squares on F^2	
Data / restraints / parameters	7845 / 0 / 545	

Goodness-of-fit on F^2	1.009
Final R indices [I>2sigma(I)]	R1 = 0.0453, wR2 = 0.1243
R indices (all data)	R1 = 0.0482, wR2 = 0.1279
Extinction coefficient	n/a
Largest diff. peak and hole	0.272 and -0.261 e.Å^{-3}

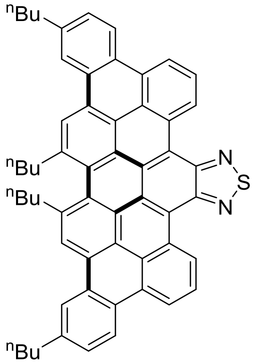

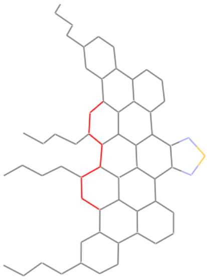

Crystal data and structure refinement for **G2**.

Identification code	**G2**	
Empirical formula	$C_{58}H_{50}N_2S$	
Formula weight	807.06	
Temperature	100(2) K	
Wavelength	1.54184 Å	
Crystal system	Monoclinic	
Space group	P 21/n	
Unit cell dimensions	a = 4.9812(5) Å	α = 90°.
	b = 26.603(2) Å	β = 90.531(8)°.
	c = 30.448(2) Å	γ = 90°.
Volume	4034.6(6) Å^3	
Z	4	
Density (calculated)	1.329 Mg/m^3	
Absorption coefficient	1.047 mm^{-1}	
F(000)	1712	

Crystal size	0.200 x 0.020 x 0.020 mm^3
Theta range for data collection	2.903 to 75.600°.
Index ranges	-6<=h<=5, -32<=k<=32, -22<=l<=37
Reflections collected	14417
Independent reflections	14417 [R(int) = ?]
Completeness to theta = 67.684°	99.7 %
Absorption correction	Semi-empirical from equivalents
Max. and min. transmission	1.00000 and 0.56711
Refinement method	Full-matrix least-squares on F^2
Data / restraints / parameters	14417 / 0 / 555
Goodness-of-fit on F^2	1.007
Final R indices [I>2sigma(I)]	R1 = 0.0999, wR2 = 0.2795
R indices (all data)	R1 = 0.1962, wR2 = 0.3079
Extinction coefficient	n/a
Largest diff. peak and hole	0.496 and -0.588 e.Å^{-3}

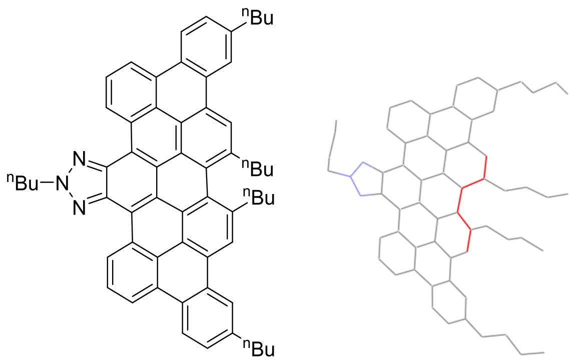

Crystal data and structure refinement for **G3**.

Empirical formula	C62 H59 N3
Formula weight	846.12
Temperature	100(2) K
Wavelength	1.54184 Å
Crystal system	monoclinic

Space group	P 21/n	
Unit cell dimensions	a = 28.6594(8) Å	α = 90°.
	b = 4.86370(10) Å	β = 91.815(2)°.
	c = 31.9917(7) Å	γ = 90°.
Volume	4457.11(18) Å^3	
Z	4	
Density (calculated)	1.261 Mg/m^3	
Absorption coefficient	0.550 mm^{-1}	
F(000)	1808	
Crystal size	0.470 x 0.070 x 0.020 mm^3	
Theta range for data collection	2.764 to 75.642°.	
Index ranges	-25<=h<=35, -5<=k<=5, -39<=l<=35	
Reflections collected	25623	
Independent reflections	8951 [R(int) = 0.0664]	
Completeness to theta = 67.684°	99.8 %	
Absorption correction	Semi-empirical from equivalents	
Max. and min. transmission	1.00 and 0.549	
Refinement method	Full-matrix least-squares on F^2	
Data / restraints / parameters	8951 / 0 / 591	
Goodness-of-fit on F^2	1.027	
Final R indices [I>2sigma(I)]	R1 = 0.0604, wR2 = 0.1549	
R indices (all data)	R1 = 0.0792, wR2 = 0.1735	
Extinction coefficient	n/a	
Largest diff. peak and hole	0.380 and -0.328 e.Å^{-3}	

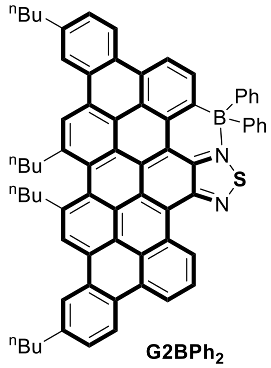

$G2BPh_2$

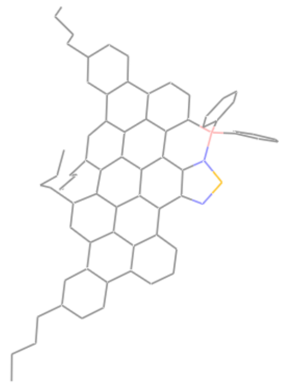

Crystal data and structure refinement for **$G2BPh_2$**.

Identification code	shelx	
Empirical formula	C70 H59 B N2 S	
Formula weight	971.06	
Temperature	100(2) K	
Wavelength	1.54184 Å	
Crystal system	Monoclinic	
Space group	P 21/n	
Unit cell dimensions	a = 9.0402(5) Å	a= 90°.
	b = 39.5093(15) Å	b= 97.574(4)°.
	c = 27.6306(9) Å	g = 90°.
Volume	9782.8(7) Å^3	
Z	8	
Density (calculated)	1.319 Mg/m^3	
Absorption coefficient	0.956 mm^{-1}	
F(000)	4112	
Crystal size	0.200 x 0.110 x 0.050 mm^3	
Theta range for data collection	2.758 to 75.941°.	
Index ranges	-11<=h<=8, -48<=k<=34, -32<=l<=34	
Reflections collected	34054	
Independent reflections	19551 [R(int) = 0.0804]	
Completeness to theta = 67.684°	99.5 %	
Absorption correction	Semi-empirical from equivalents	
Max. and min. transmission	1.00 and 0.639	
Refinement method	Full-matrix least-squares on F^2	
Data / restraints / parameters	19551 / 1193 / 1414	
Goodness-of-fit on F^2	1.082	

Final R indices [I>2sigma(I)]	R1 = 0.1016, wR2 = 0.2519
R indices (all data)	R1 = 0.1804, wR2 = 0.3168
Extinction coefficient	n/a
Largest diff. peak and hole	0.640 and -0.456 e.$Å^{-3}$

Spectra

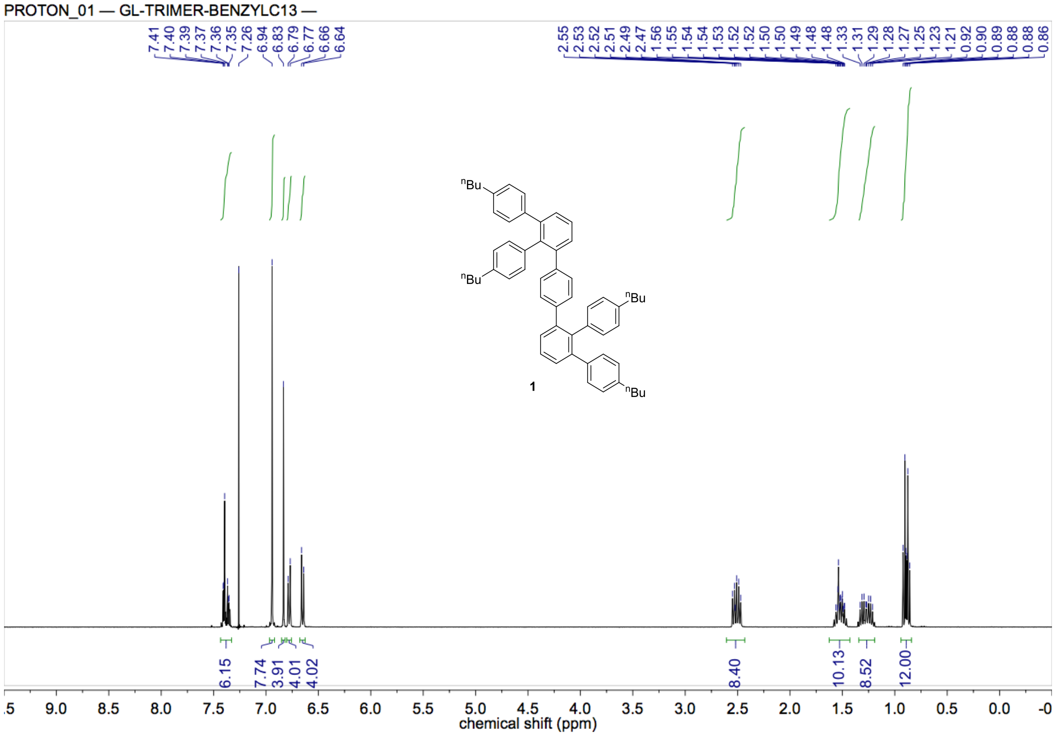

Supplementary Figure 20. ^{1}H NMR spectrum of **1** in $CDCl_3$.

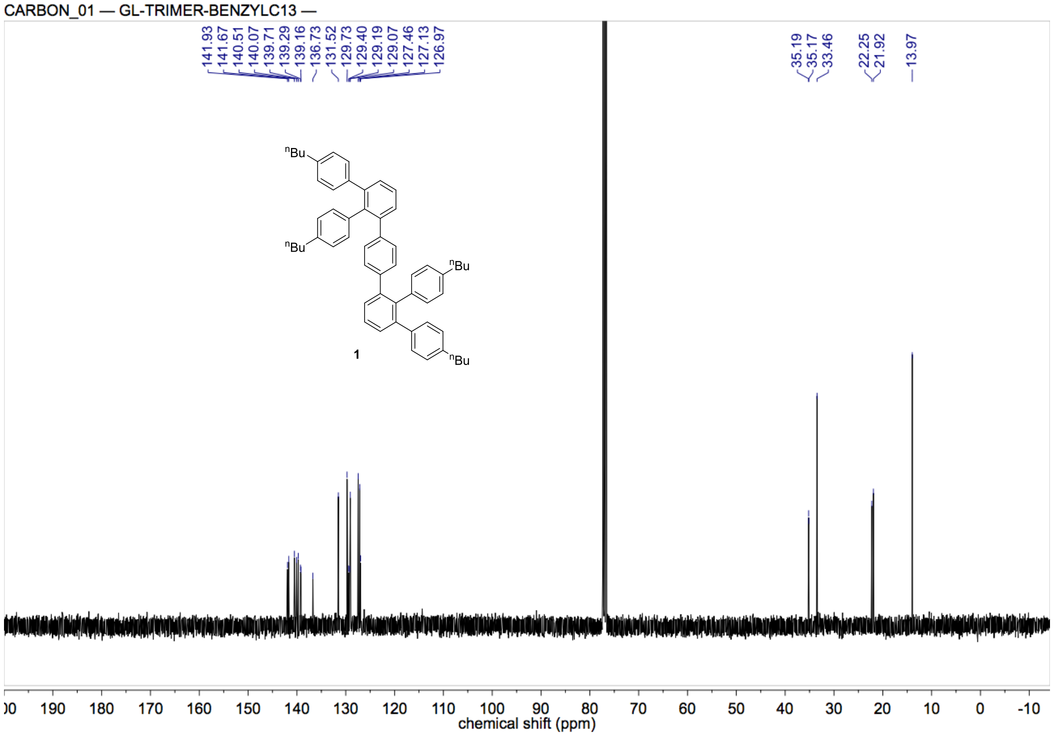

Supplementary Figure 21. ^{13}C NMR spectrum of **1** in $CDCl_3$.

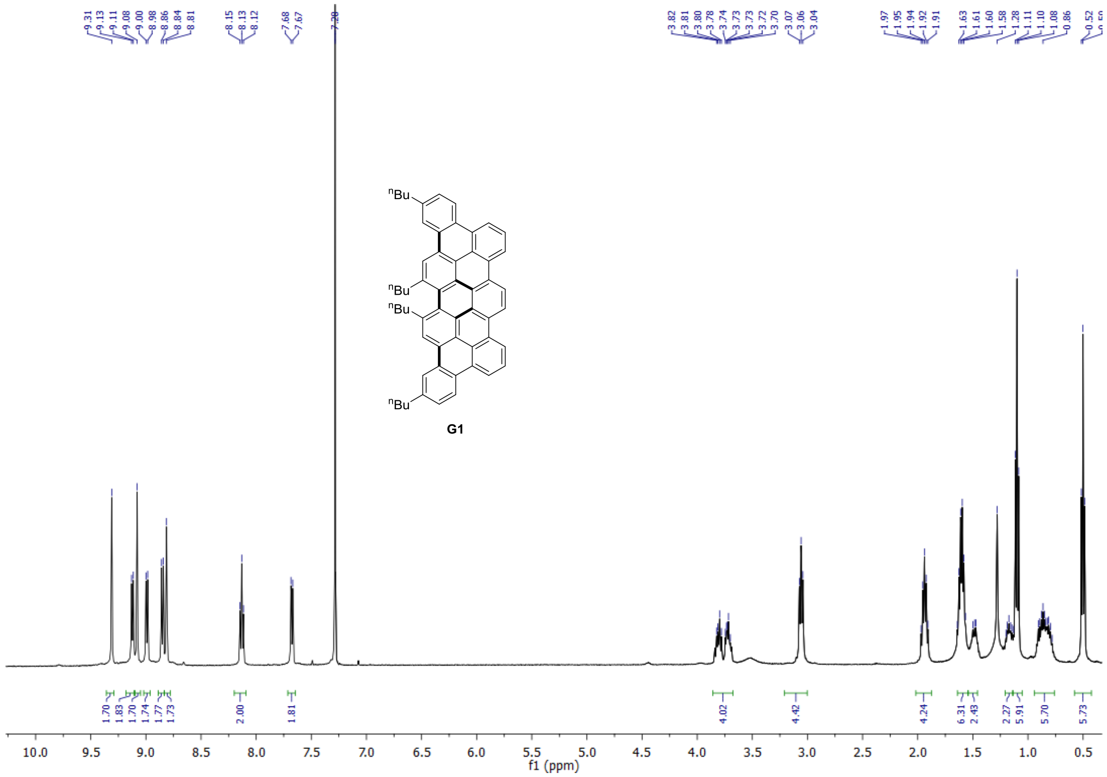

Supplementary Figure 22. ^{1}H NMR spectrum of **G1** in $CDCl_3$.

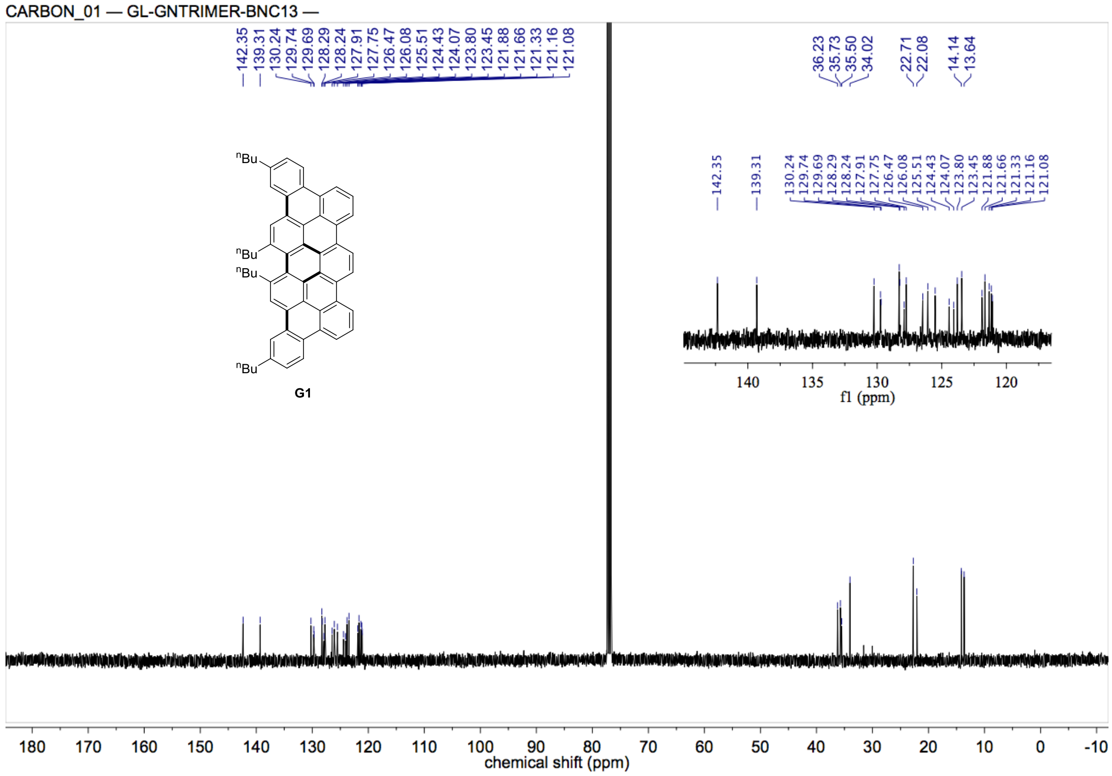

Supplementary Figure 23. ^{13}C NMR spectrum of **G1** in $CDCl_3$.

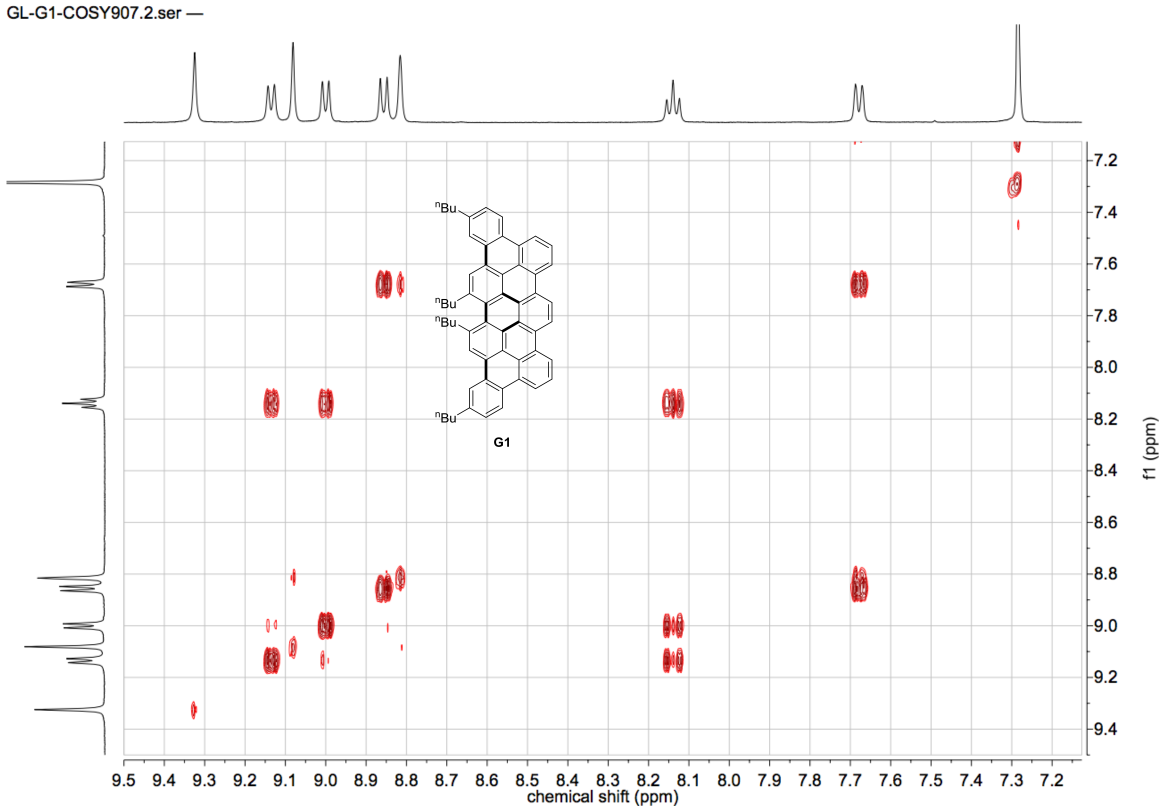

Supplementary Figure 24. Homo-Cosy NMR spectrum of **G1** in $CDCl_3$.

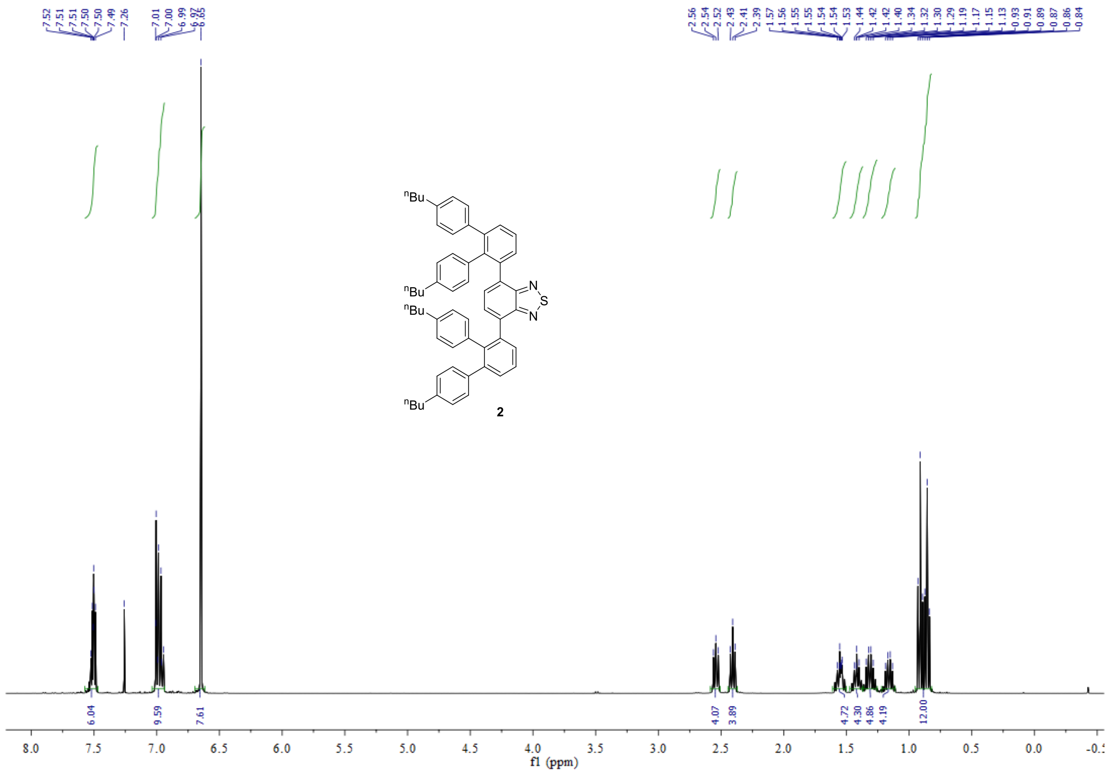

Supplementary Figure 25. ^{1}H NMR spectrum of **2** in $CDCl_3$.

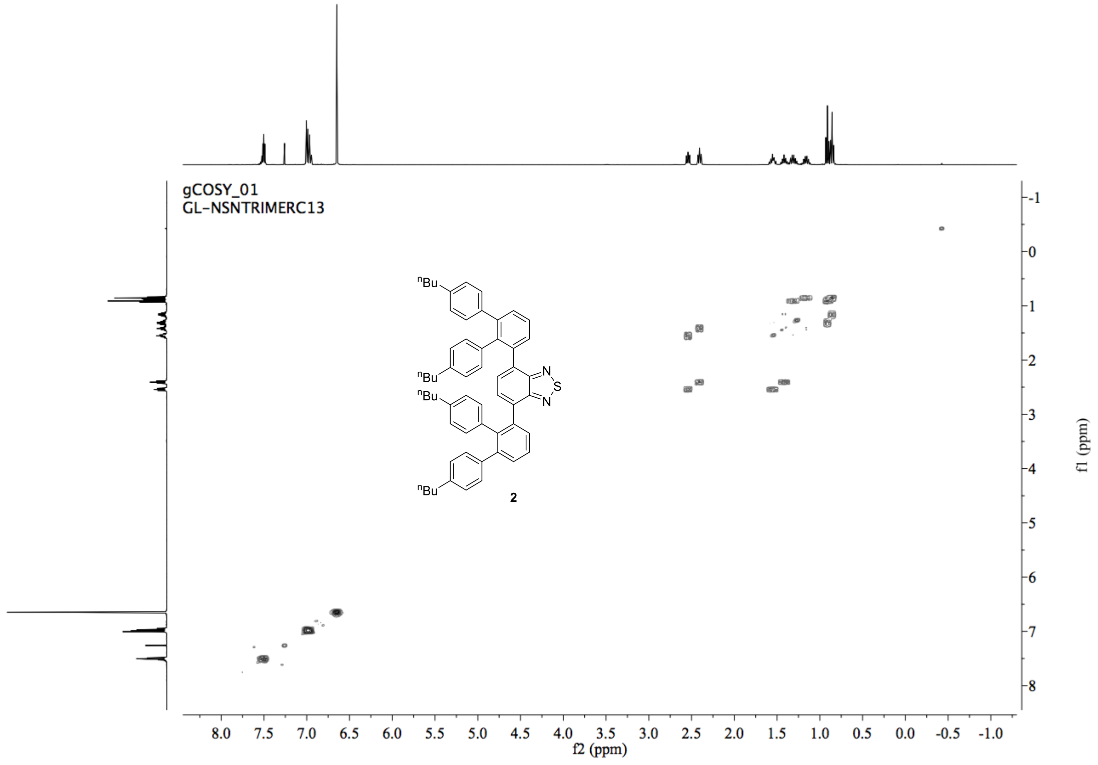

Supplementary Figure 26. Homo-Cosy NMR spectrum of **2** in $CDCl_3$.

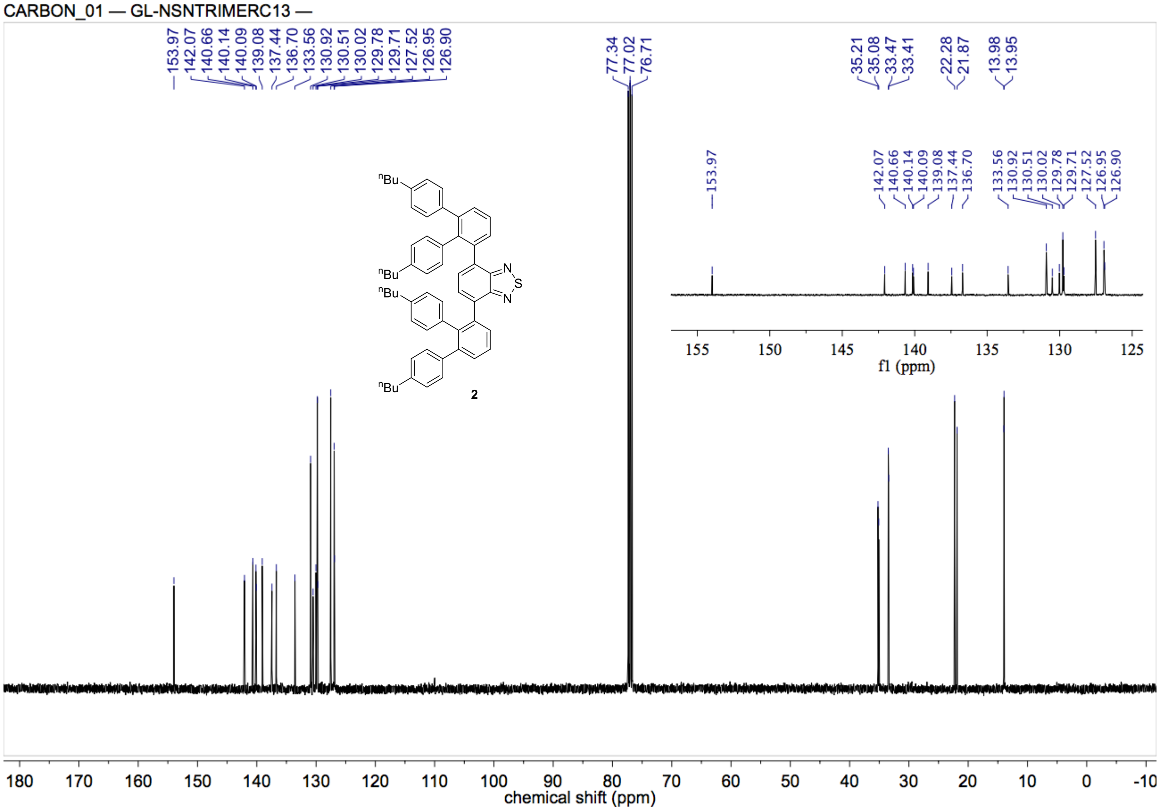

Supplementary Figure 27. ^{13}C NMR spectrum of **2** in $CDCl_3$.

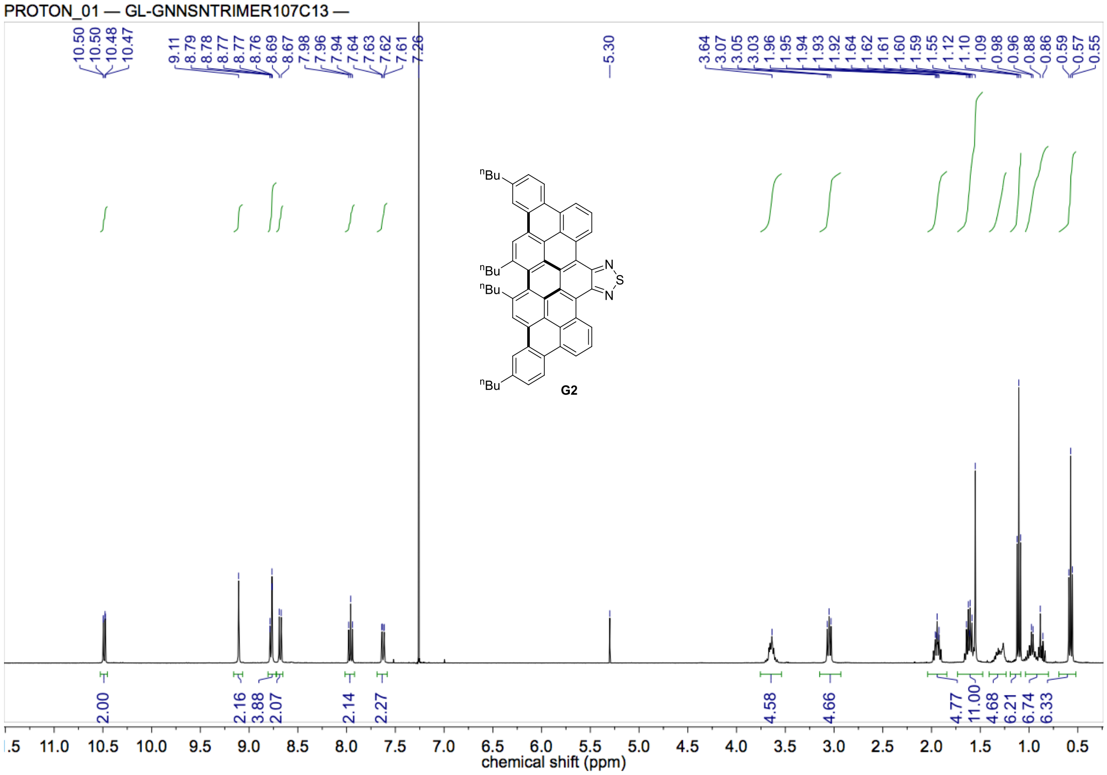

Supplementary Figure 28. ^{1}H NMR spectrum of **G2** in $CDCl_3$.

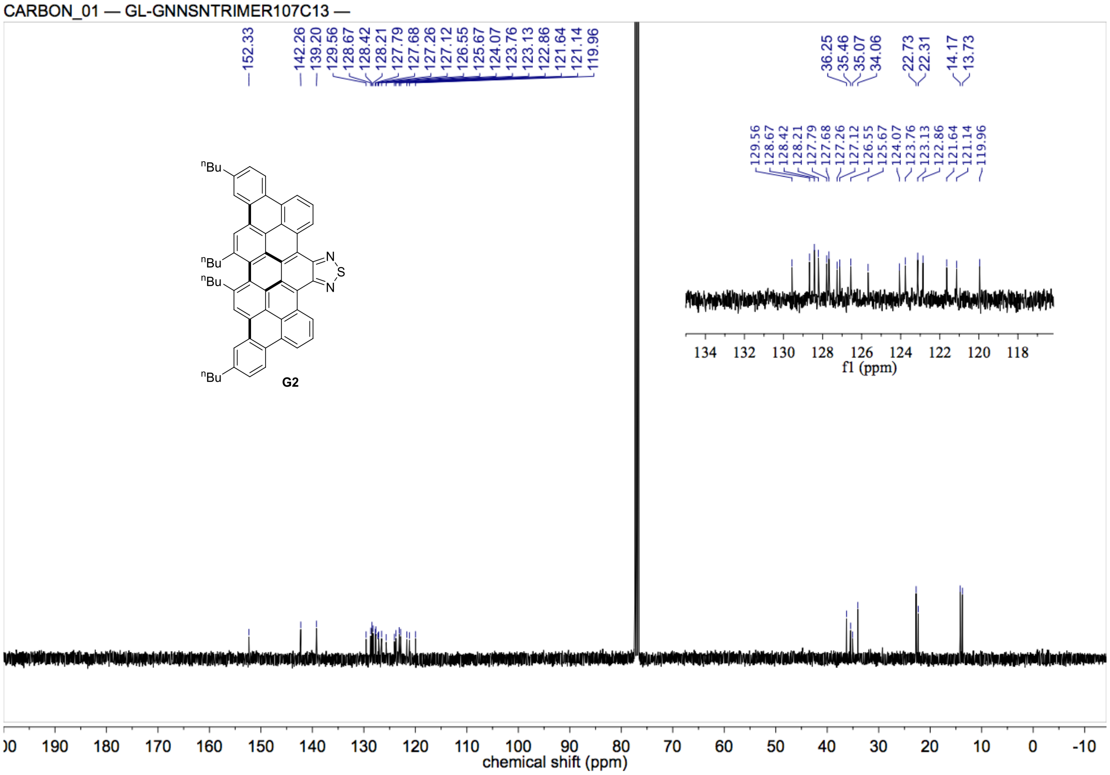

Supplementary Figure 29. ^{13}C NMR spectrum of **G2** in $CDCl_3$.

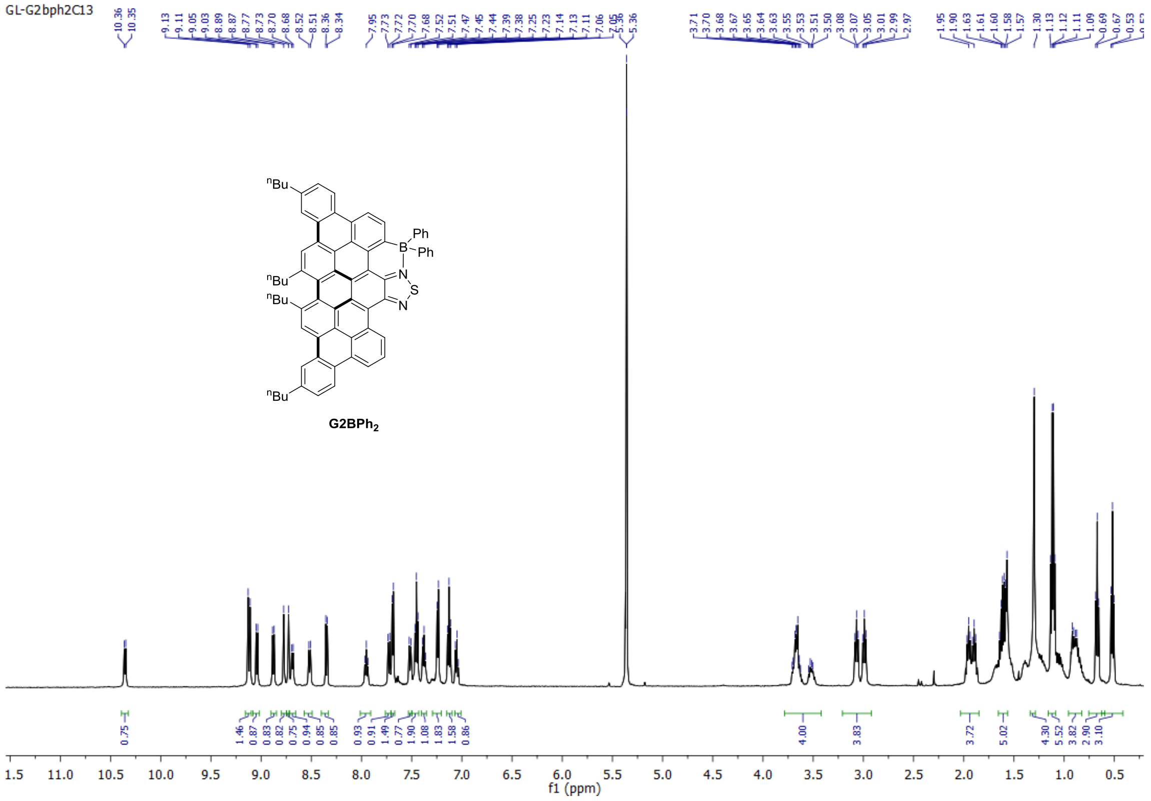

Supplementary Figure 30. ^{1}H NMR spectrum of **G2BPh$_2$** in CD_2Cl_2.

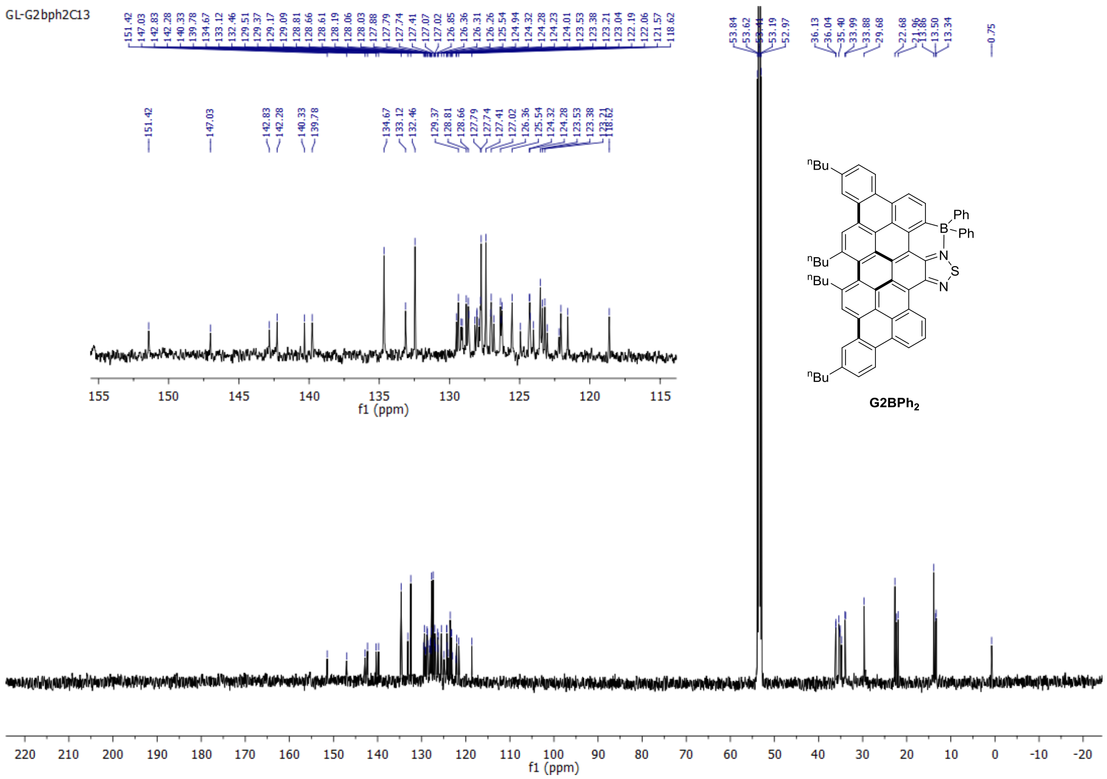

Supplementary Figure 31. ^{13}C NMR spectrum of **G2BPh$_2$** in CD_2Cl_2.

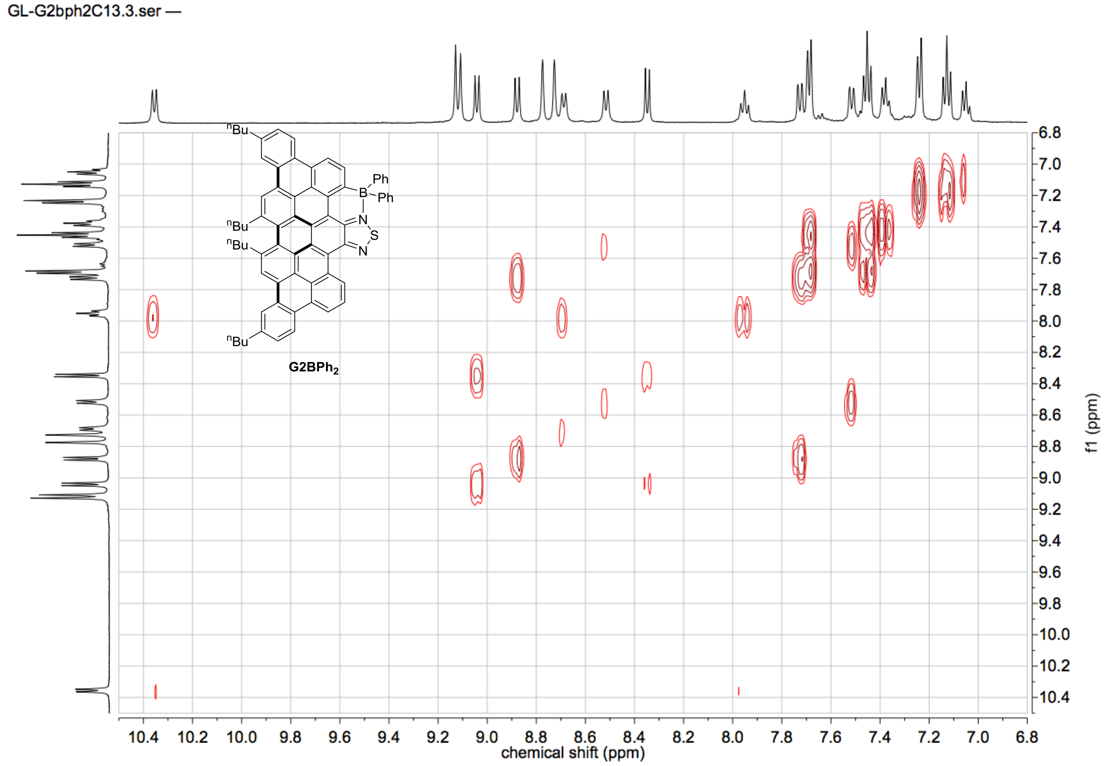

Supplementary Figure 32. Homo-Cosy NMR spectrum of **G2BPh$_2$** in CD_2Cl_2.

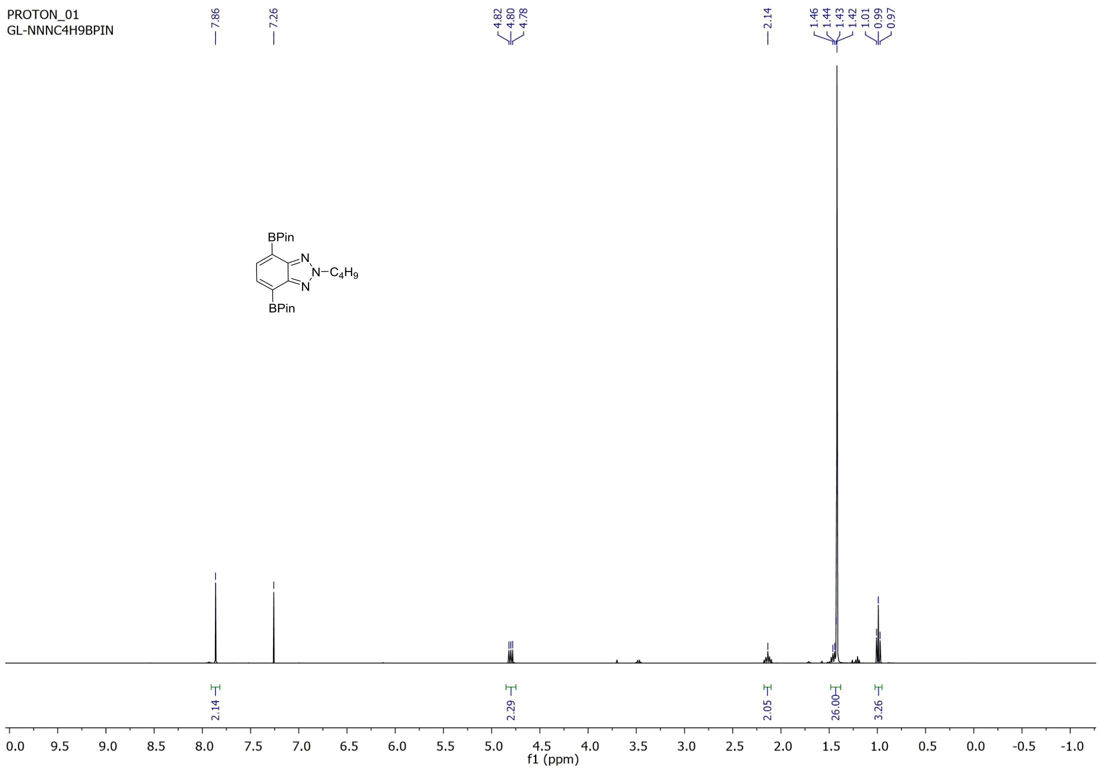

Supplementary Figure 33. ^{1}H NMR spectrum of **M3** in $CDCl_3$.

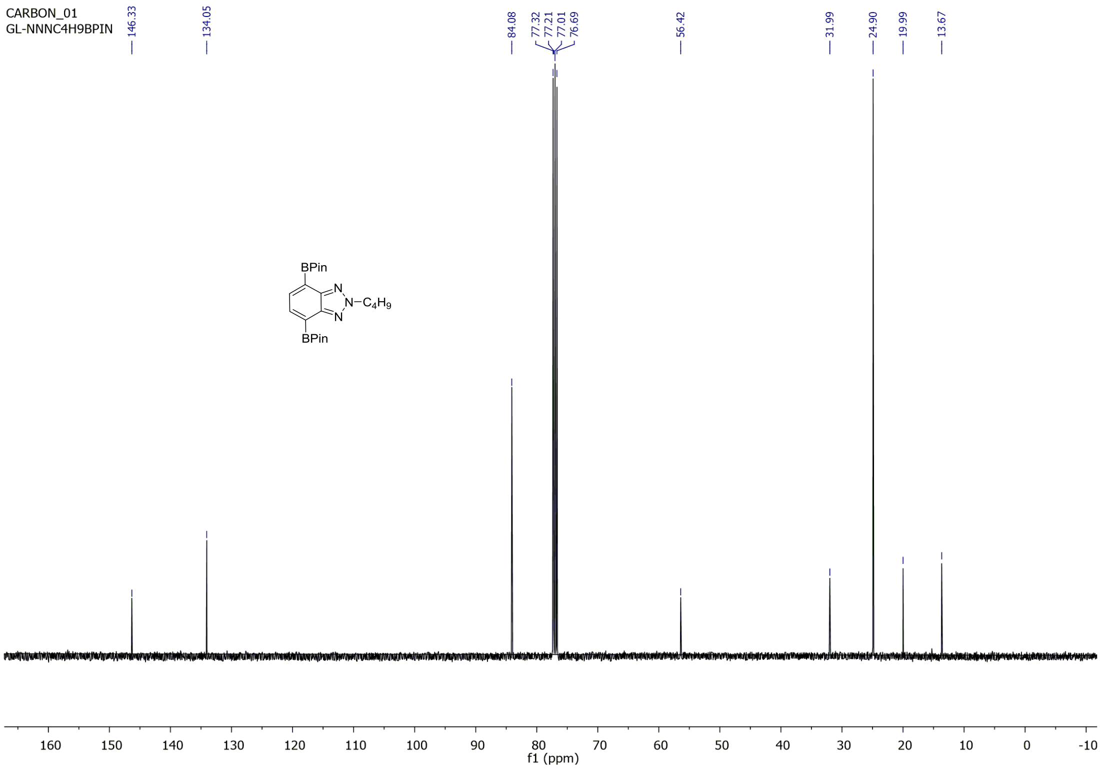

Supplementary Figure 34. ^{13}C NMR spectrum of **M3** in $CDCl_3$.

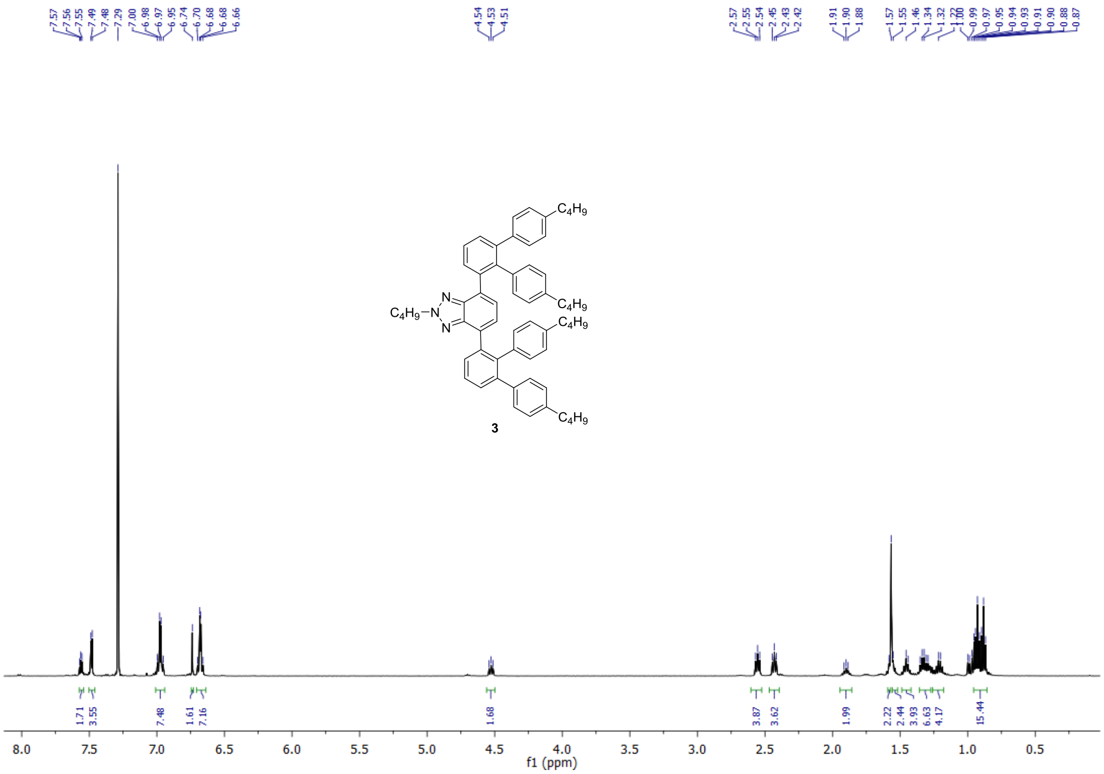

Supplementary Figure 35. ^{1}H NMR spectrum of **3** in $CDCl_3$.

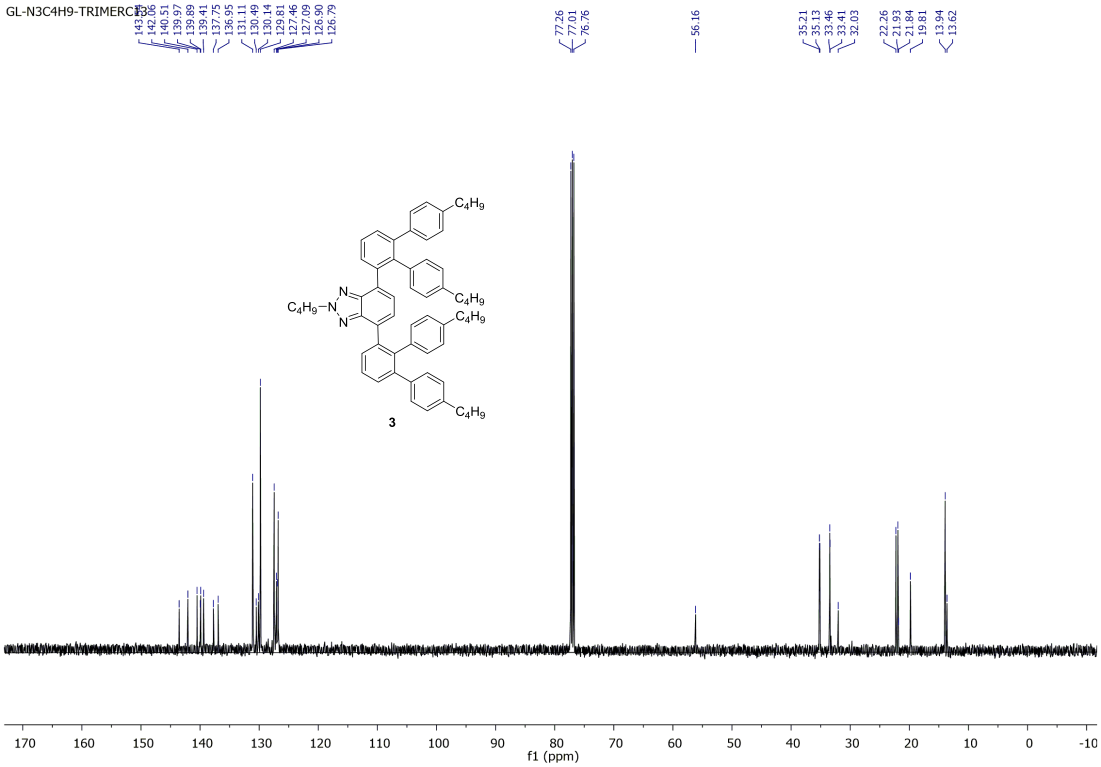

Supplementary Figure 36. ^{13}C NMR spectrum of **3** in $CDCl_3$.

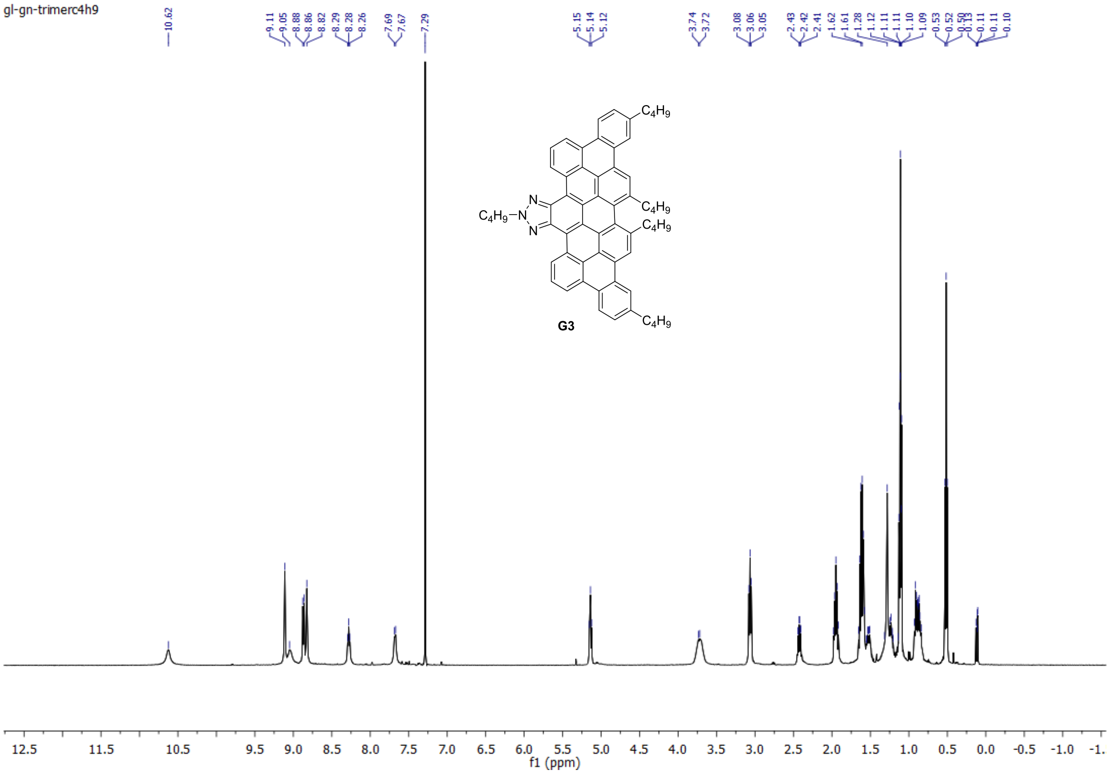

Supplementary Figure 37. ^{1}H NMR spectrum of **G3** in $CDCl_3$.

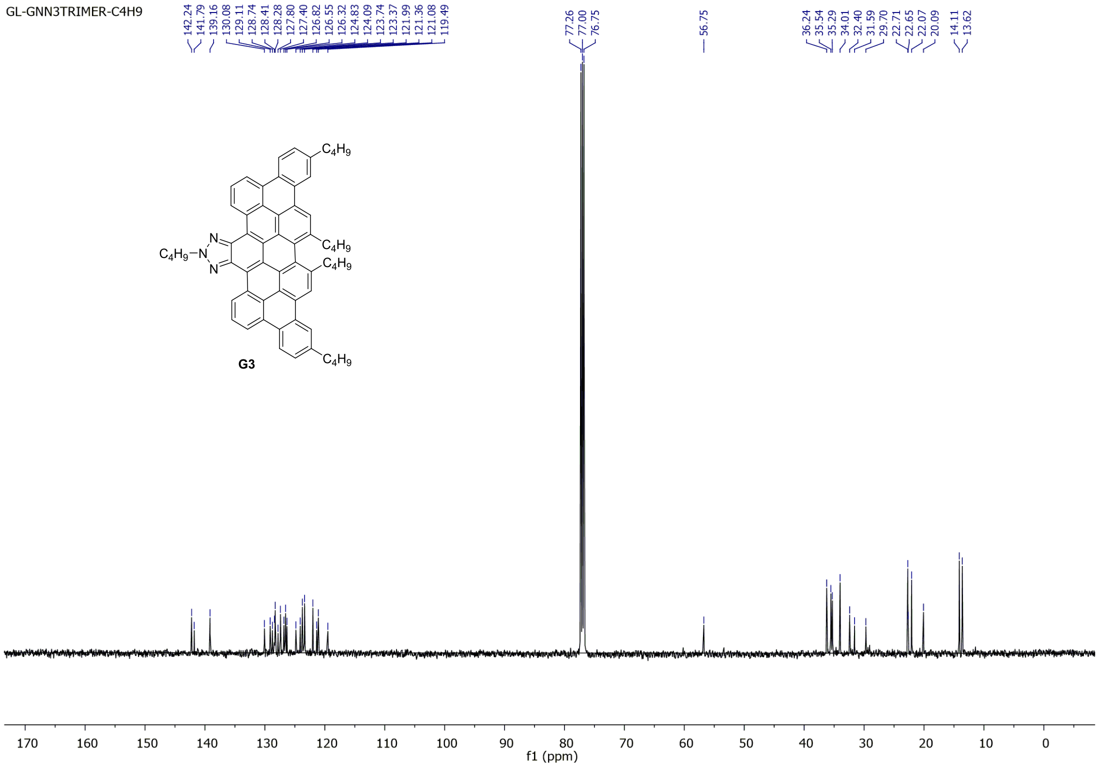

Supplementary Figure 38. ^{13}C NMR spectrum of **G3** in $CDCl_3$.

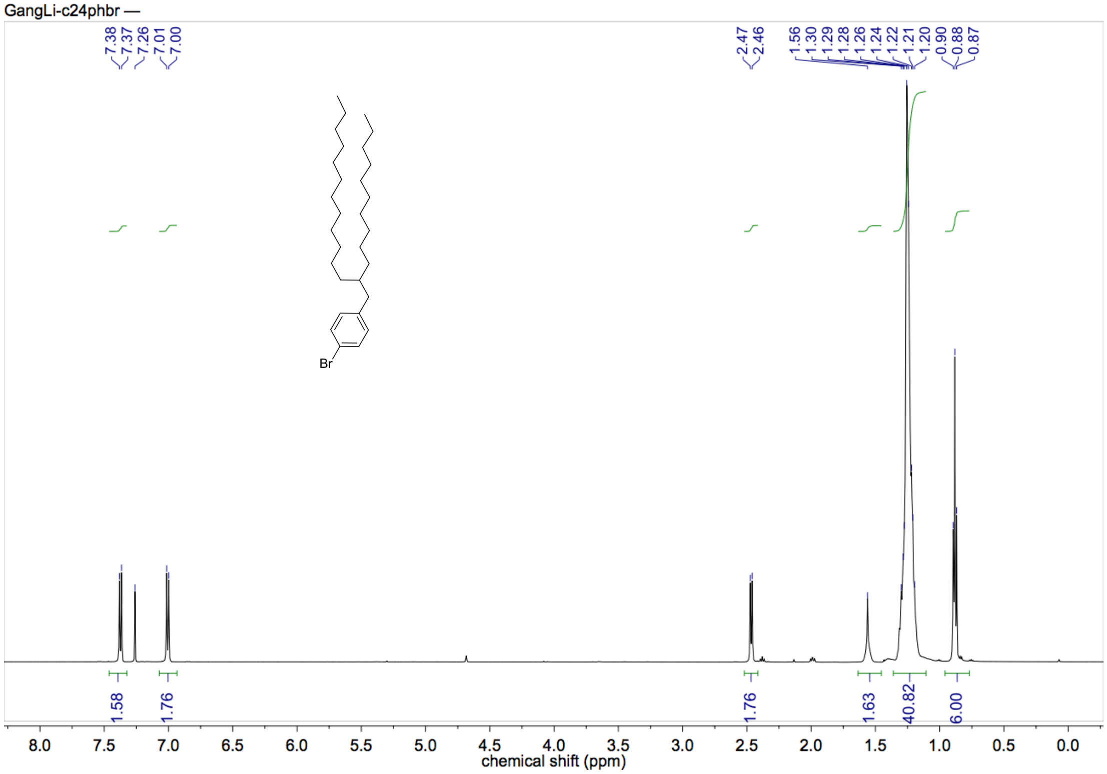

Supplementary Figure 39. ^{1}H NMR spectrum of **S6** in $CDCl_3$.

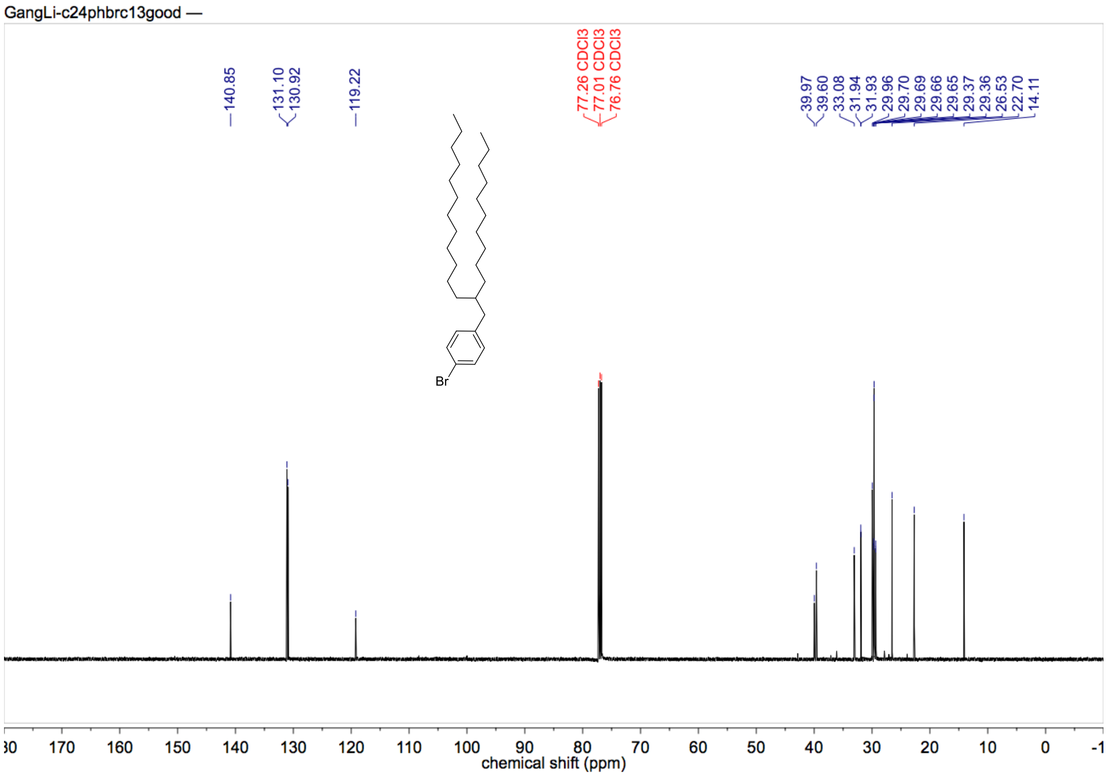

Supplementary Figure 40. ^{13}C NMR spectrum of **S6** in $CDCl_3$.

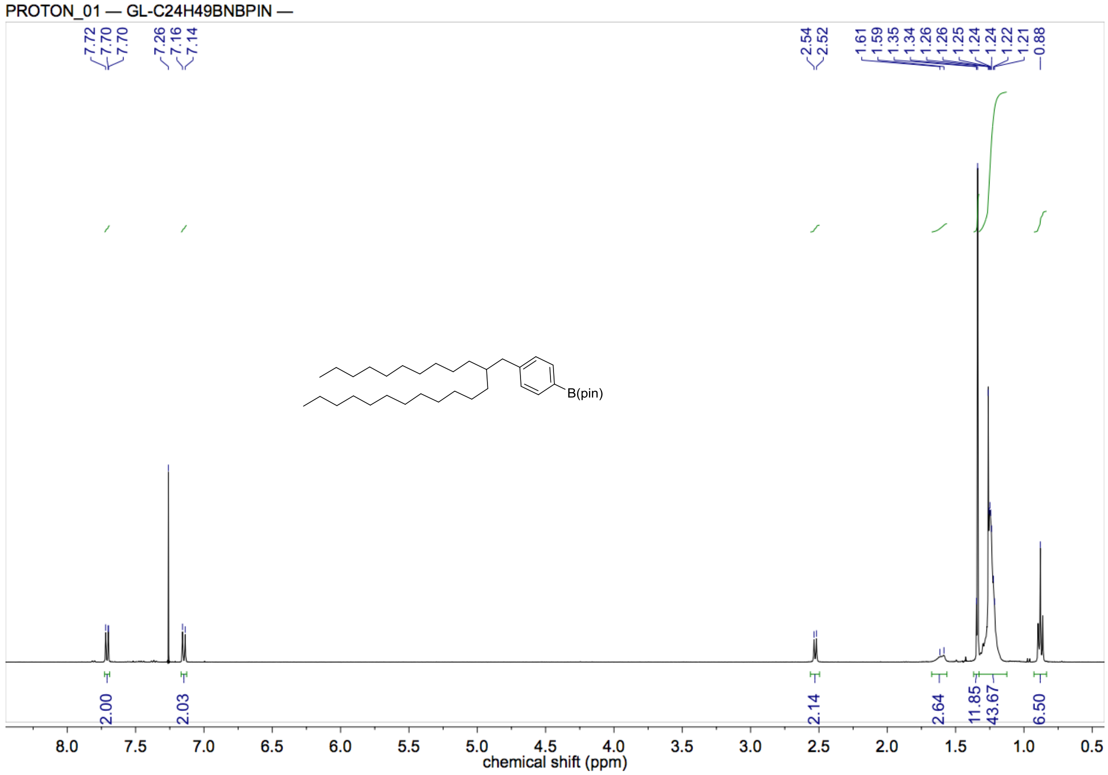

Supplementary Figure 41. ^{1}H NMR spectrum of **S7** in $CDCl_3$.

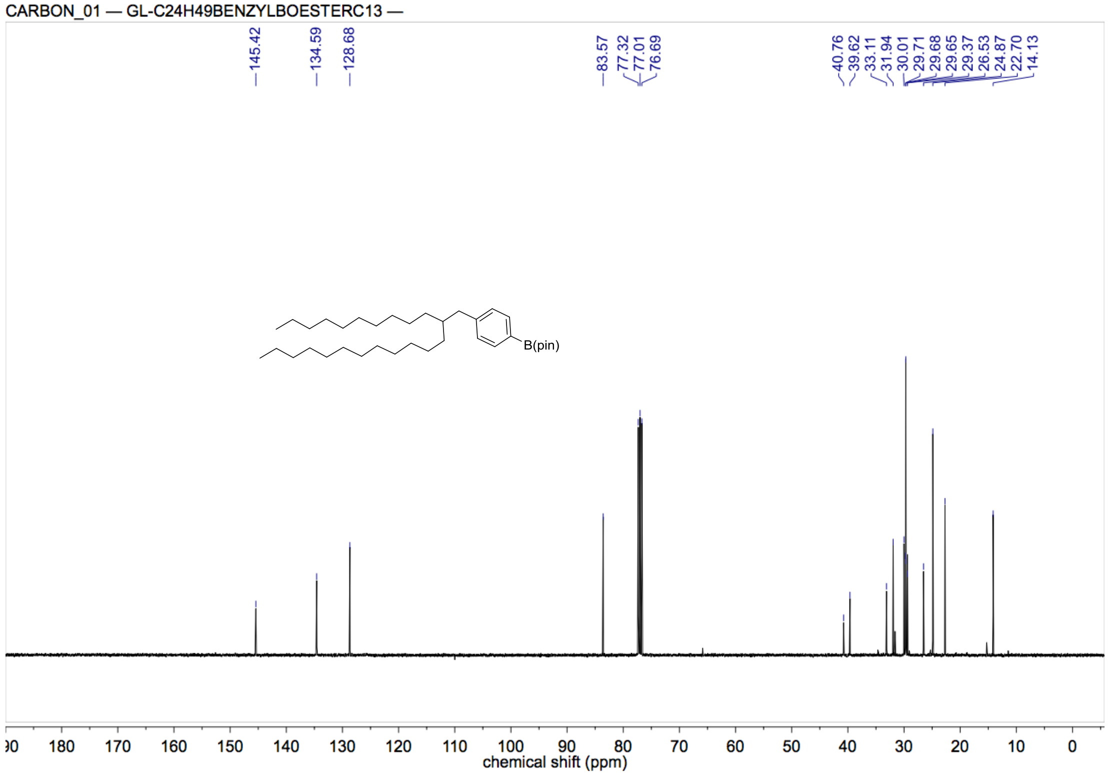

Supplementary Figure 42. ^{13}C NMR spectrum of **S7** in $CDCl_3$.

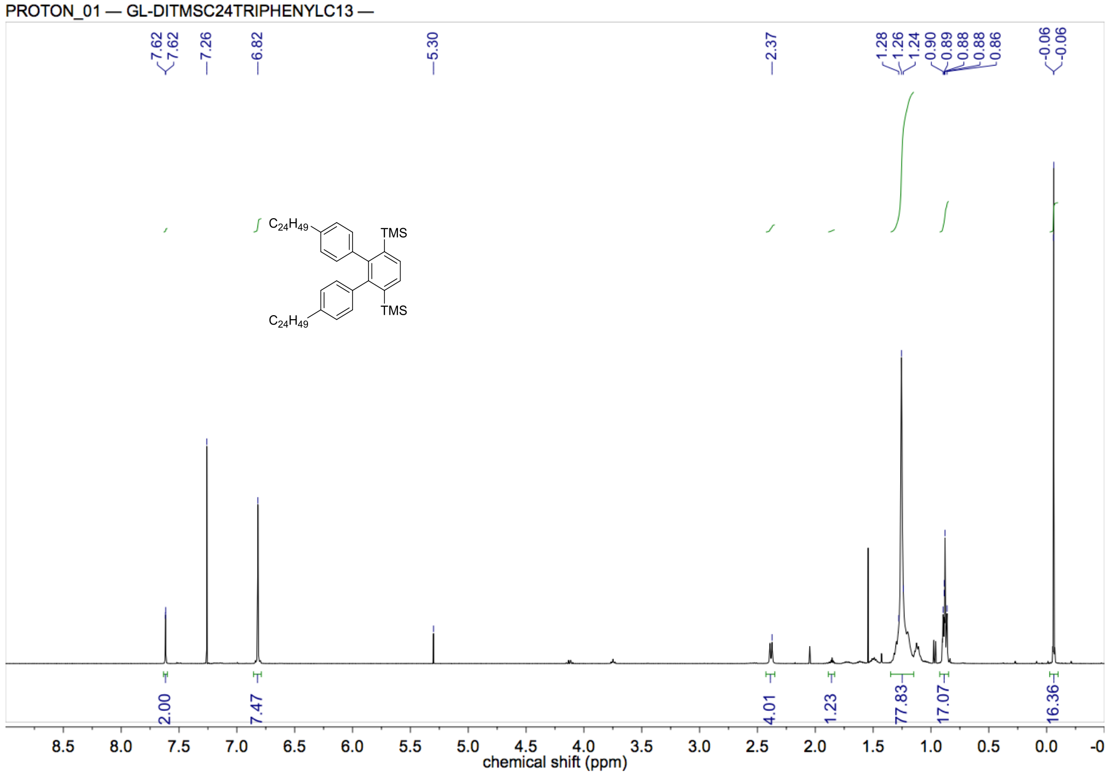

Supplementary Figure 43. ^{1}H NMR spectrum of **S9** in $CDCl_3$.

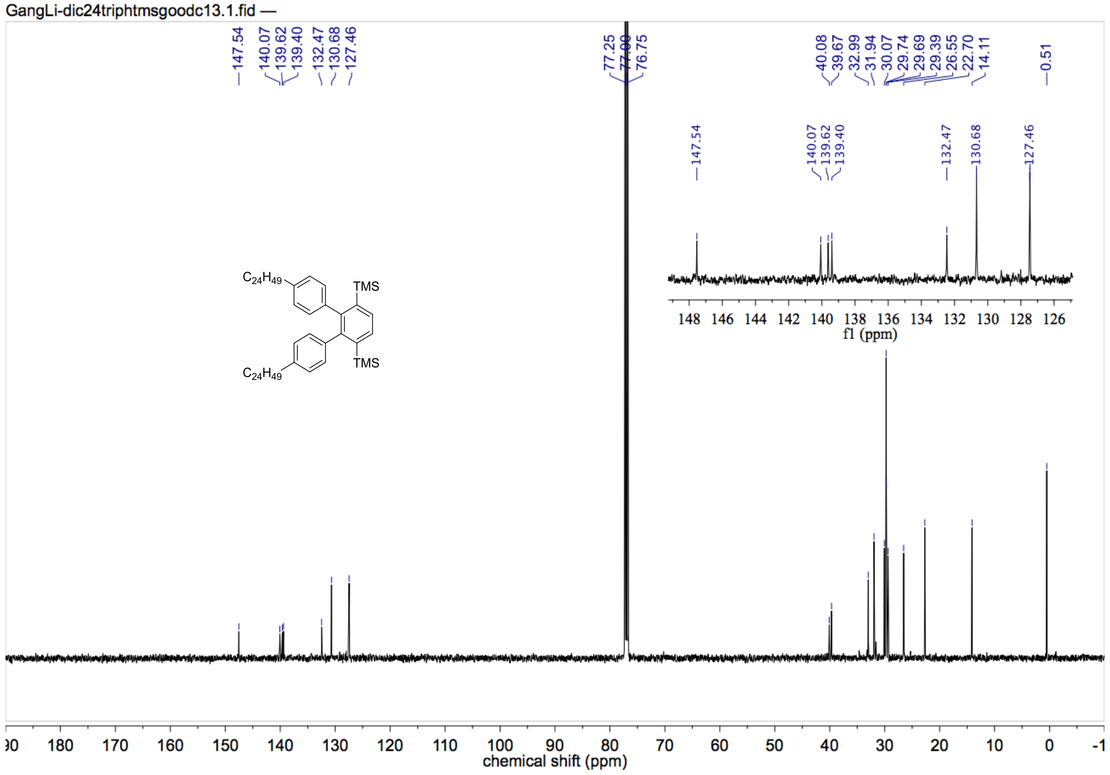

Supplementary Figure 44. ^{13}C NMR spectrum of **S9** in $CDCl_3$.

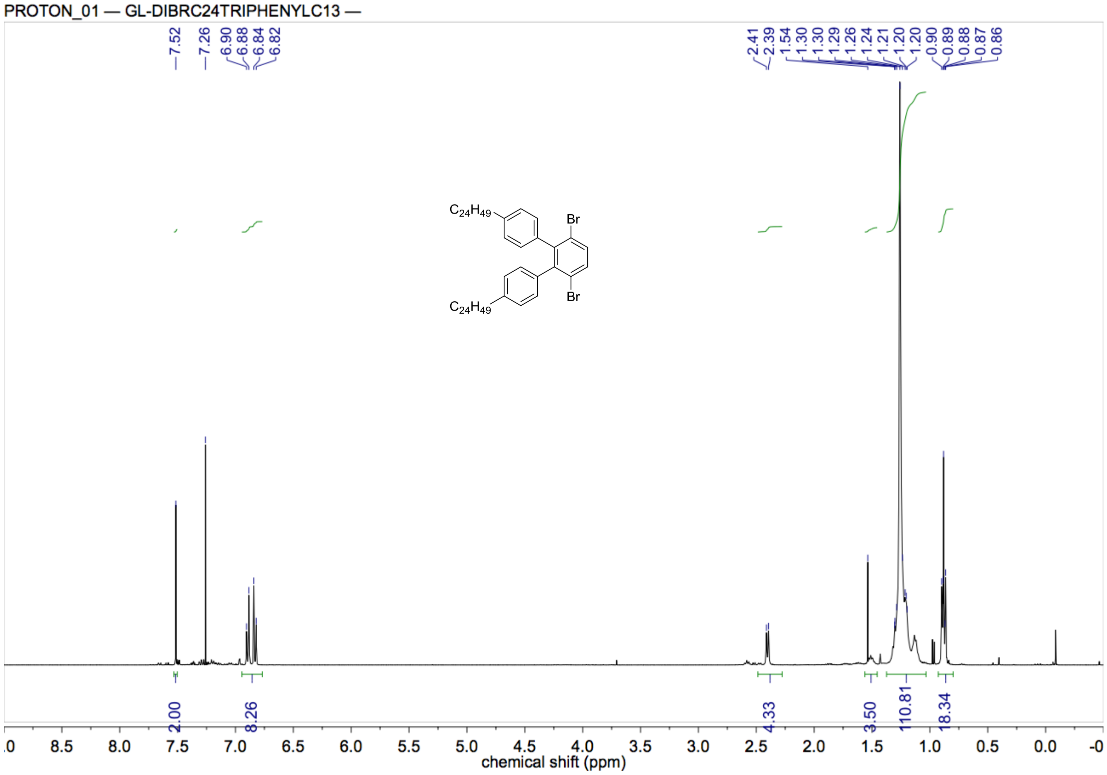

Supplementary Figure 45. ^{1}H NMR spectrum of **M4’** in $CDCl_3$.

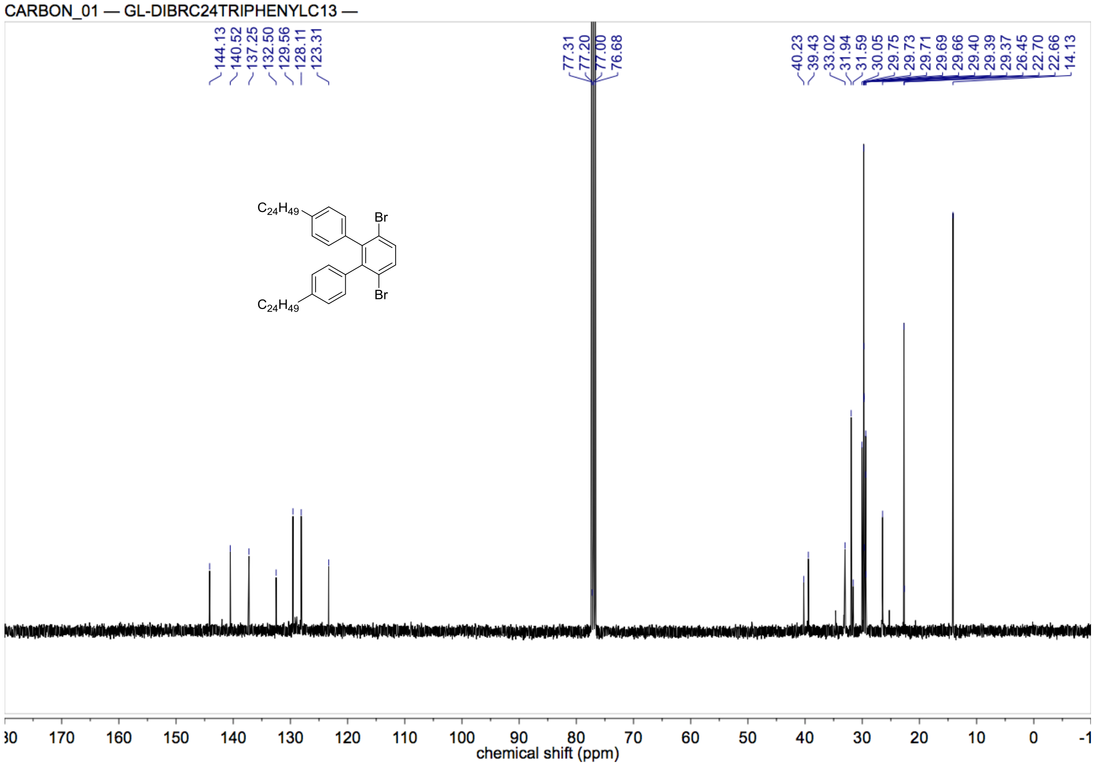

Supplementary Figure 46. ^{13}C NMR spectrum of **M4’** in $CDCl_3$.

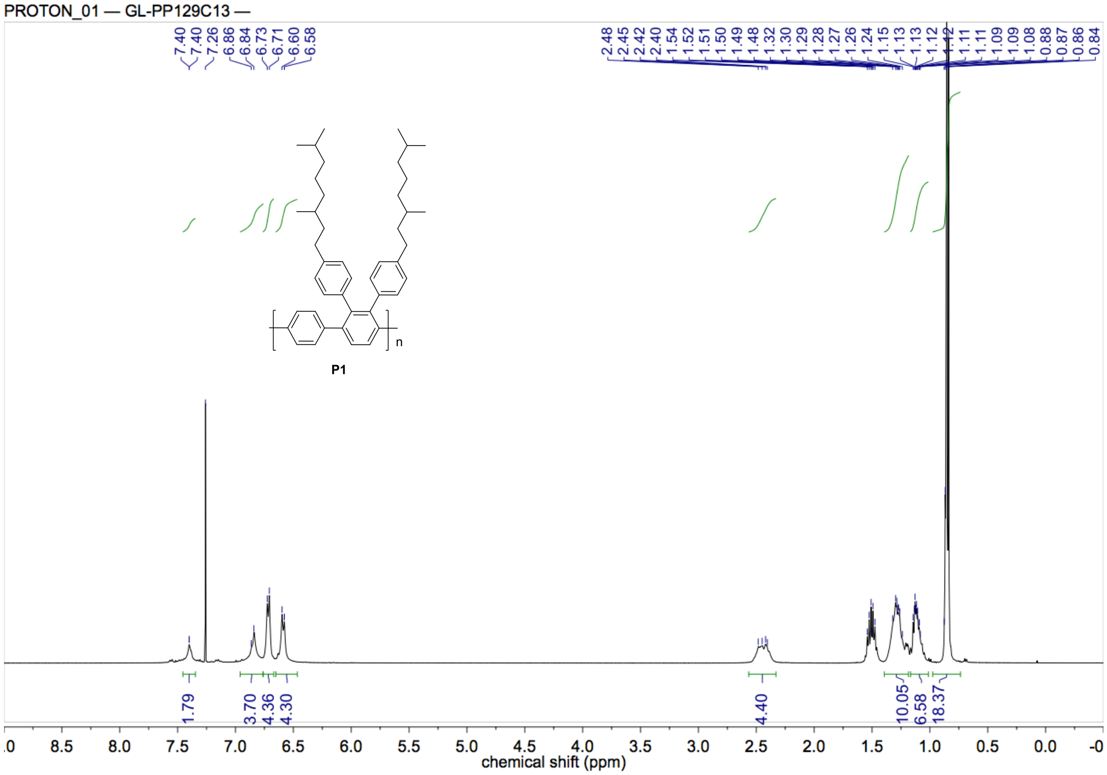

Supplementary Figure 47. ^{1}H NMR spectrum of **P1** in $CDCl_3$.

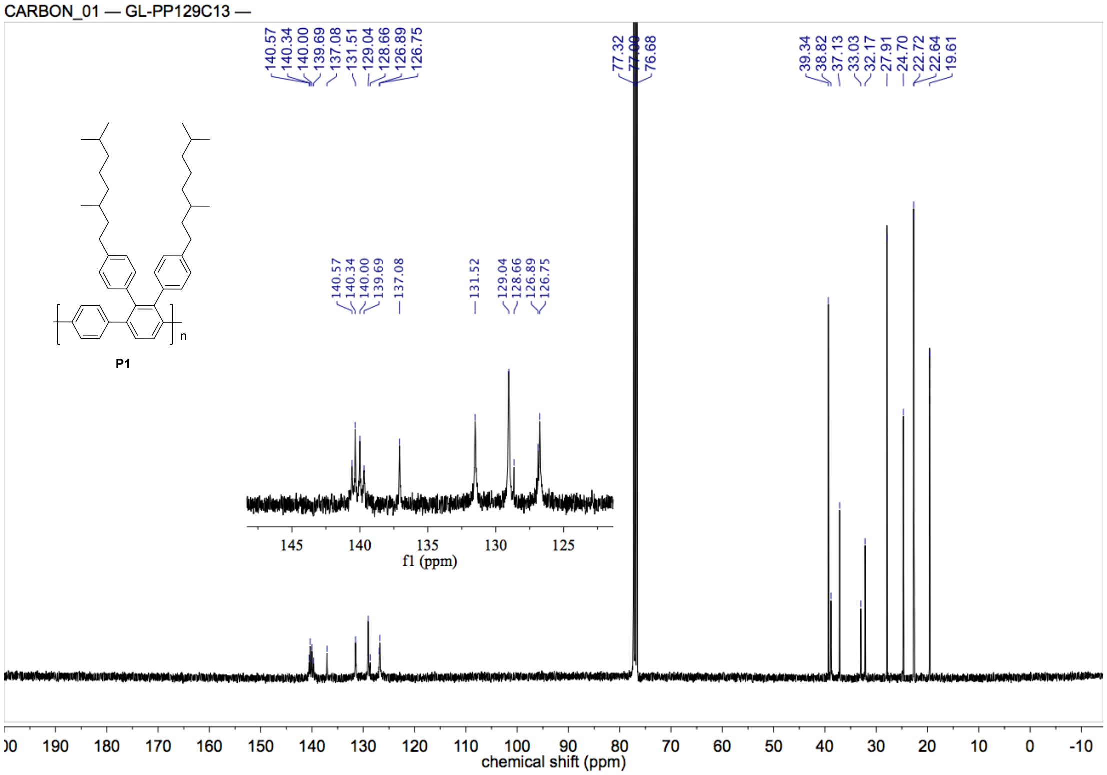

Supplementary Figure 48. ^{13}C NMR spectrum of **P1** in $CDCl_3$.

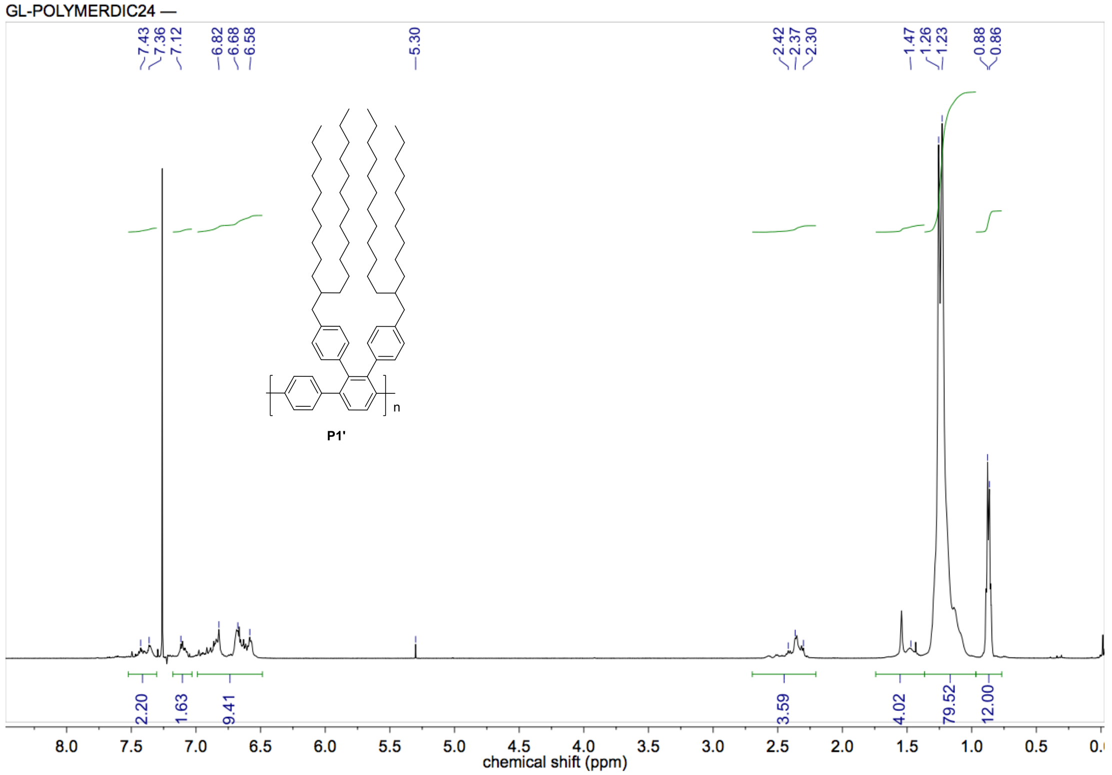

Supplementary Figure 49. ^{1}H NMR spectrum of **P1’** in $CDCl_3$.

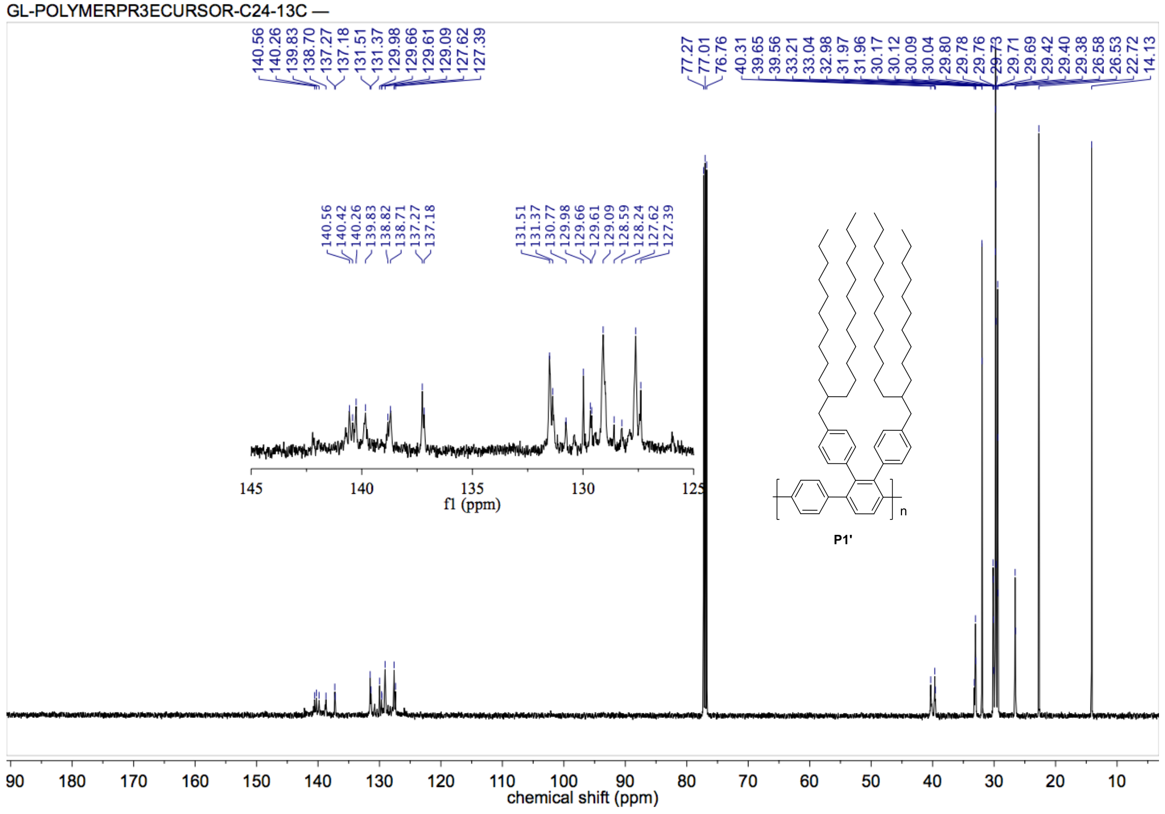

Supplementary Figure 50. ^{13}C NMR spectrum of **P1’** in $CDCl_3$.

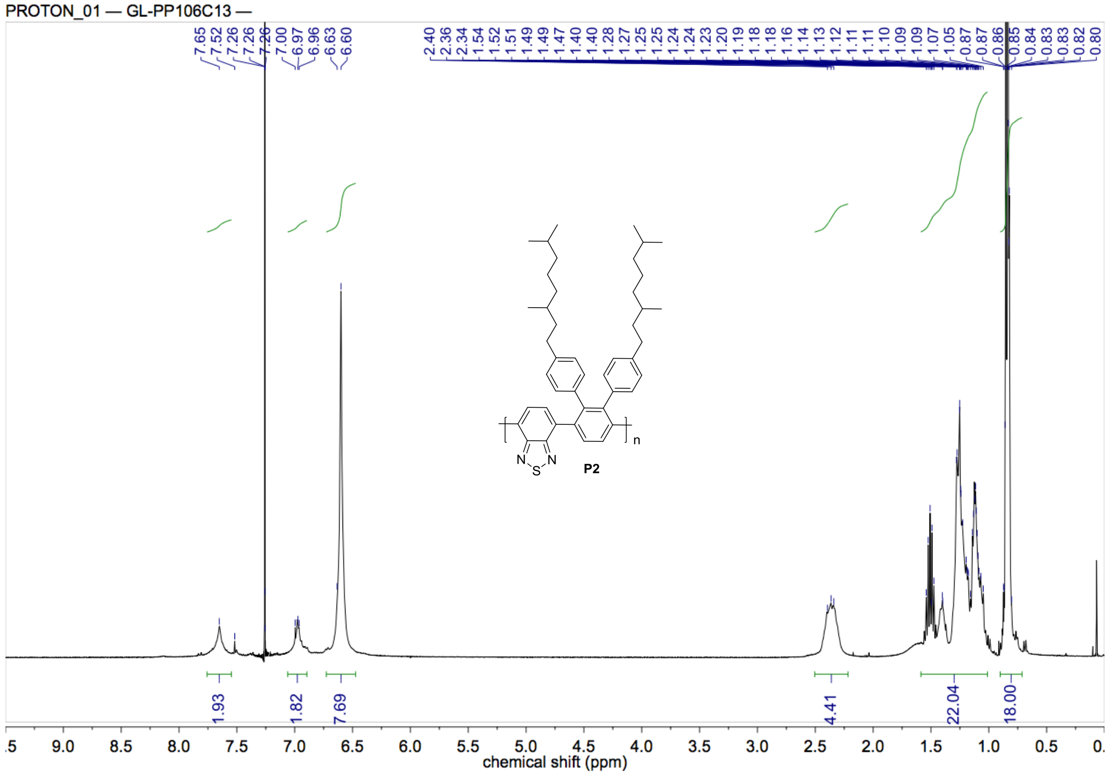

Supplementary Figure 51. ^{1}H NMR spectrum of **P2** in $CDCl_3$.

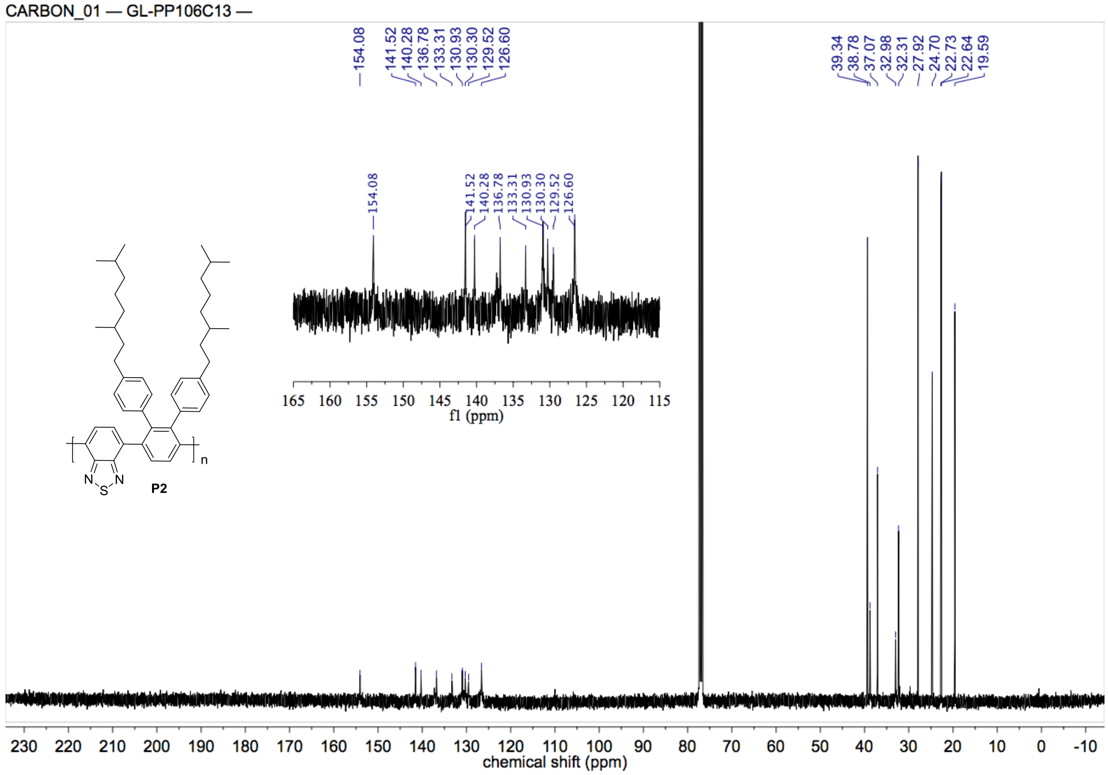

Supplementary Figure 52. ^{1}H NMR spectrum of **P2** in $CDCl_3$.

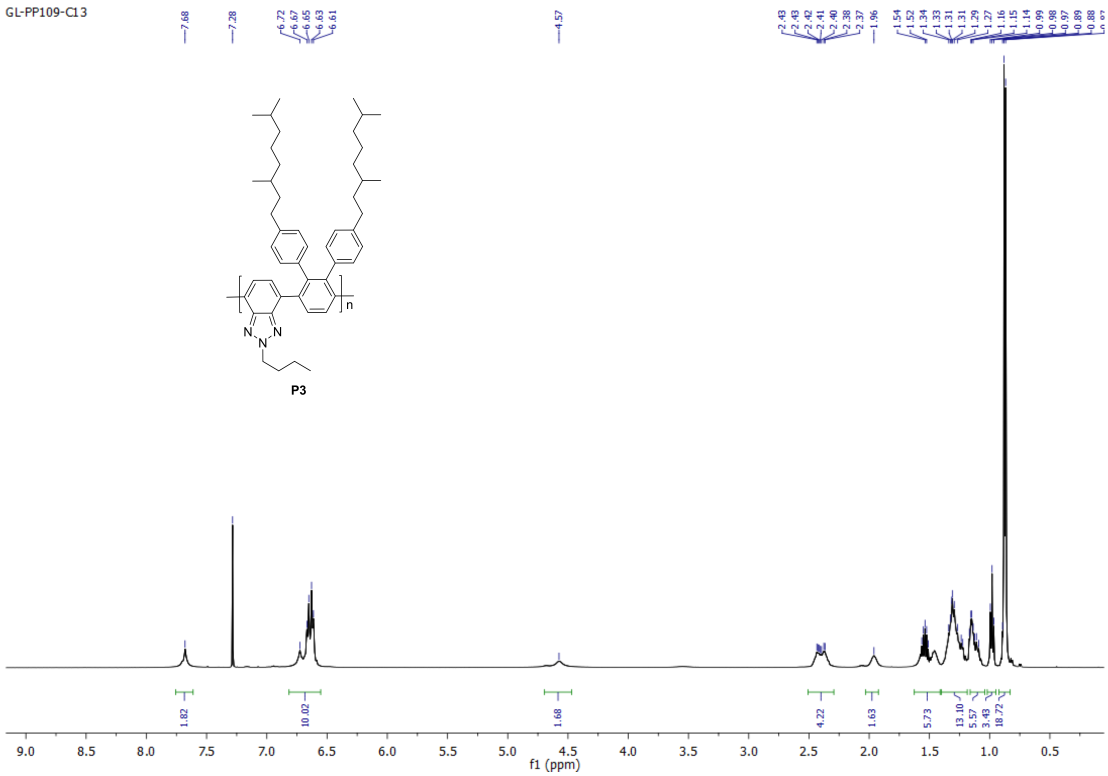

Supplementary Figure 53. ^{1}H NMR spectrum of **P3** in $CDCl_3$.

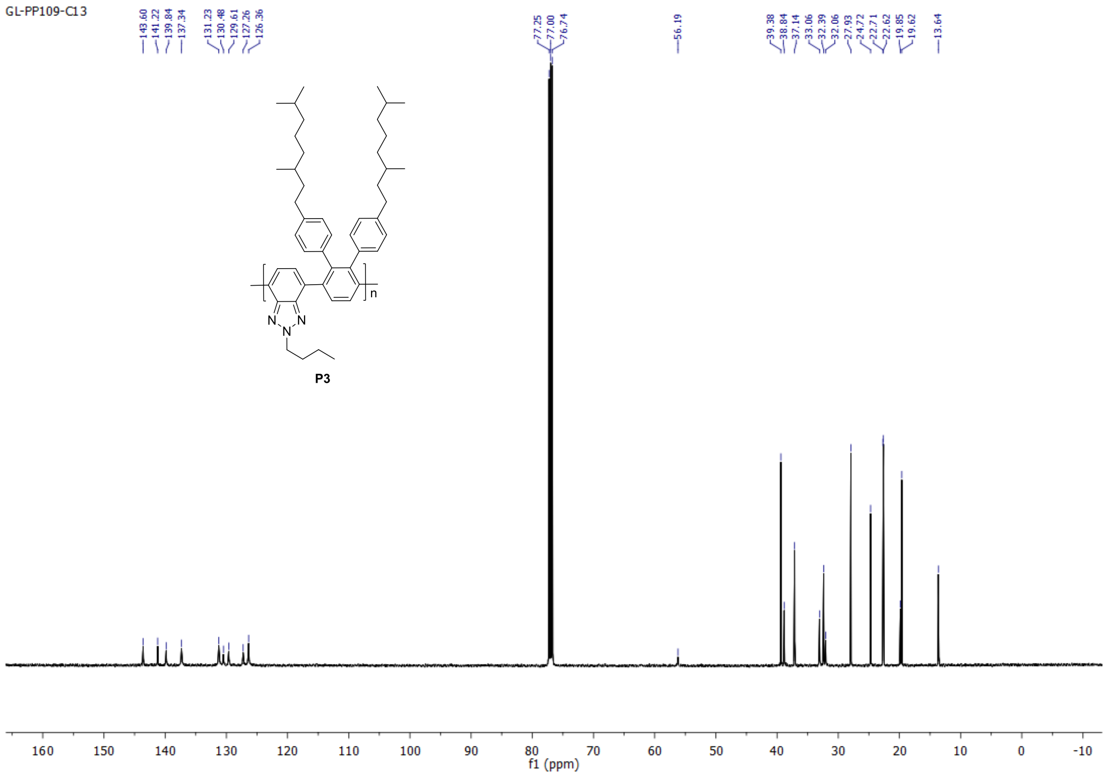

Supplementary Figure 54. ^{13}C NMR spectrum of **P3** in $CDCl_3$.

References

(1) Beams, R., Cançado, L. G., Novotny, L. Raman characterization of defects and dopants in graphene. *J. Phys.: Condens. Matter* **27**, 083002 (2015).

(2) Viezbicke, B. D., Patel, S., Davis, B. E. & Birnie III, B. P. Evaluation of the Tauc method for optical absorption edge determination: ZnO thin films as a model system. *Phys. Status Solidi B* **252**, 1700-1710 (2015).

(3) Sakaguchi, H. *et. al.* Width-controlled sub-nanometer graphene nanoribbons films synthesized by radical-polymerized chemical vapor deposition. *Adv. Mater.* **26**, 4134-4138 (2014).

(4) M. J. Frisch, G. W. Trucks, H. B. Schlegel, G. E. Scuseria, M. A. Robb, J. R. Cheeseman, G. Scalmani, V. Barone, B. Mennucci, G. A. Petersson, H. Nakatsuji, M. Caricato, X. Li, H. P. Hratchian, A. F. Izmaylov, J. Bloino, G. Zheng, J. L. Sonnenberg, M. Hada, M. Ehara, K. Toyota, R. Fukuda, J. Hasegawa, M. Ishida, T. Nakajima, Y. Honda, O. Kitao, H. Nakai, T. Vreven, J. A. Montgomery Jr., J. E. Peralta, F. Ogliaro, M. J. Bearpark, J. Heyd, E. N. Brothers, K. N. Kudin, V. N. Staroverov, R. Kobayashi, J. Normand, K. Raghavachari, A. P. Rendell, J. C. Burant, S. S. Iyengar, J. Tomasi, M. Cossi, N. Rega, N. J. Millam, M. Klene, J. E. Knox, J. B. Cross, V. Bakken, C. Adamo, J. Jaramillo, R. Gomperts, R. E. Stratmann, O. Yazyev, A. J. Austin, R. Cammi, C. Pomelli, J. W. Ochterski, R. L. Martin, K. Morokuma, V. G. Zakrzewski, G. A. Voth, P. Salvador, J. J. Dannenberg, S. Dapprich, A. D. Daniels, Ö. Farkas, J. B. Foresman, J. V. Ortiz, J. Cioslowski, D. J. Fox, Gaussian, Inc., Wallingford, CT, USA 2009.

(5) Li, G., Yoon, K.-Y., Zhong, X., Zhu, X.-Y. & Dong, G. Efficient bottom-up preparation of graphene nanoribbons by mild Suzuki-miyaura polymerization of simple triaryl monomers. *Chem. Eur. J.* **22**, 9116-9120 (2016).

(6) Bheemireddy, S. R., Hautzinger, M. P., Li, T., Lee, B., Plunkett, K. N. Conjugated Ladder Polymers by a Cyclopentannulation Polymerization. *J. Am. Chem. Soc.* **139**, 5801-5807 (2017).
