# Supplementary material for: A modular synthetic approach for band-gap engineering of armchair graphene nanoribbons
Source: Nat Commun. 2018 Apr 27;9:1687. doi: 10.1038/s41467-018-03747-2 (PMC5924368; doi:10.1038/s41467-018-03747-2)
Supplement: Supplementary file 2 — Peer Review File(PDF 245 kb) [file 41467_2018_3747_MOESM2_ESM.pdf]

Peer Review File for Transferred Manuscript

Editorial Note: This manuscript has been previously reviewed at another journal that is not operating a transparent peer review scheme. The manuscript was considered suitable for publication without further review at *Nature Communications.*
